# A chameleon AIEgen exhibiting six distinct yet tunable thermal and photoswitchable states

**DOI:** 10.1038/s41467-025-61717-x

**Published:** 2025-07-09

**Authors:** Xinyuan He, Baochuan Hu, Xin Wang, Xing Feng, Xinyuan Wang, Xinmeng Chen, Jianwei Sun, Jacky W. Y. Lam, Lianrui Hu, Ben Zhong Tang

**Affiliations:** 1 Department of Chemistry, and the Hong Kong Branch of Chinese National Engineering Research Center for Tissue Restoration and Reconstruction, The Hong Kong University of Science and Technology, Clear Water Bay, Kowloon, Hong Kong China; 2 Shanghai Key Laboratory of Green Chemistry and Chemical Processes, Shanghai Frontiers Science Center of Molecule Intelligent Syntheses, School of Chemistry and Molecular Engineering, East China Normal University, Shanghai, 200062 China; 3 College of Chemistry and Chemical Engineering, Inner Mongolia Key Laboratory of Fine Organic Synthesis, Institutes of Biomedical Sciences, Inner Mongolia University, Hohhot, 010021 China; 4 School of Material and Energy, Guangdong University of Technology, Guangzhou, 510006 China; 5 Center for Aggregation-Induced Emission, South China University of Technology, Guangzhou, 510640 China; 6 School of Science and Engineering, Shenzhen Institute of Aggregate Science and Technology, The Chinese University of Hong Kong, Shenzhen, Guangdong, 518172 China

**Keywords:** Optical materials, Information storage, Organic molecules in materials science

## Abstract

Seeking methods to realize multiple fluorescence changes in a single luminogenic system is of great importance for both chemistry and bionics research. Due to the lack of effective strategies and functional motifs, luminogens with multiple switching and controllable models are still scarce. Herein, we report a chromone-based aggregation-induced emission luminogen called *Z*-CDPM, which exhibit six distinct, tunable thermal and photoswitchable states, offering controllable thermochromic or photochromic behavior under varying conditions. Specifically, five different reactions are involved: reversible *Z*/*E* isomerization, irreversible cyclization and elimination under thermal treatment, and photoarrangement of *Z*-CDPM and its thermal cyclization product under UV irradiation. The relative independence of the switching states is effectively maintained. Experimental and theoretical analyses validate our design strategies and provide valuable insights into the detailed mechanisms of these reactions, and single crystals further confirm their structures. Additionally, practical applications, including multiple-colored images, quick response codes, and an advanced information encryption system, are developed to demonstrate the utility. This work thus provides effective strategies and structural motifs for the design of multiresponsive luminogens and multifunctional systems.

## Abstract (web summary)

Achieving multiple fluorescence changes in a single luminogenic system is desirable but challenging. Here, the authors report a chromone-based aggregation-induced emission luminogen, with six distinct thermal and photoswitchable states.

## Introduction

The excellent coloration in animals has long fascinated scientists^1–3^, which yet provides a serendipity model system for the development of bionic intelligence technology^4–7^. For instance, chameleons and cuttlefishes perform concealing coloration for camouflage^8,9^. Crested ibis shows a shiny coloration during mating season to maintain communication^10^. To pursue multiple functions in artificial systems, one promising approach is to utilize stimuli-responsive luminogens to imitate such colorful patterns of animals^11,12^. However, it usually requires a complex combination of luminogens since most reported molecules are mono-responsive^13–16^. With the advancement in synthetic organic chemistry, a single multiresponsive luminogenic system is desired for simplicity and repeatability. But currently reported multiresponsive luminogens often display sustained changes in response to a single stimulus, limiting the diversification of control modes and hindering their practical applications as intelligent materials^17–22^. Besides, the idea of constructing multifunctional luminogens by incorporating different types of functional units in one molecule remains challenging due to the lack of proper strategies and functional motifs^18,23–25^. Specifically, it would be difficult to maintain the activity of each functional unit and the corresponding spectral changes in such a multicomponent molecule^26,27^. Herein, seeking methods to realize multiple chemical switching and simultaneous color switching in a single molecule is of significant importance for both chemistry and bionics research.

In 1865, the German chemist August Kekulé proposed the structure of the benzene ring, which introduced the well-known property of aromaticity, as well as the cyclic configuration of organic molecules^28,29^. Subsequently, the ring-switching process between the ring-opening form and the ring-closing form has been reported. Classic molecules such as spiropyrans^30–32^, diarylethenes^33–35^, dihydroazulenes^36,37^ and stilbenes^18,19,38^ provide avenues for the design of stimuli-responsive molecules. Benefit from their fast, efficient, and catalyst-free switching processes in response to external stimuli, and the accompanying optical signal changes, their applications in artificial chameleon systems has also been demonstrated. However, the single switching model for each molecule inevitably limits the scope of their applications. In recent years, a series of works investigating the structure-activity relationship in these switchable systems have been done, including: (i) the positive influence of electron-withdrawing groups (EWGs) on cyclization efficience^17,18,37–39^, (ii) and the feasibility of achieving a combinational output via ring-flipping process^40,41^. With the growing abundance of the functional motif library and the success of on-demand generation of complex ring-closing state in molecules, luminogens with multiple ring-closing states are anticipated to achieve multiresponse. Yet, it would be challenging to ensure the independence of each switching process.

**Fig. 1:**
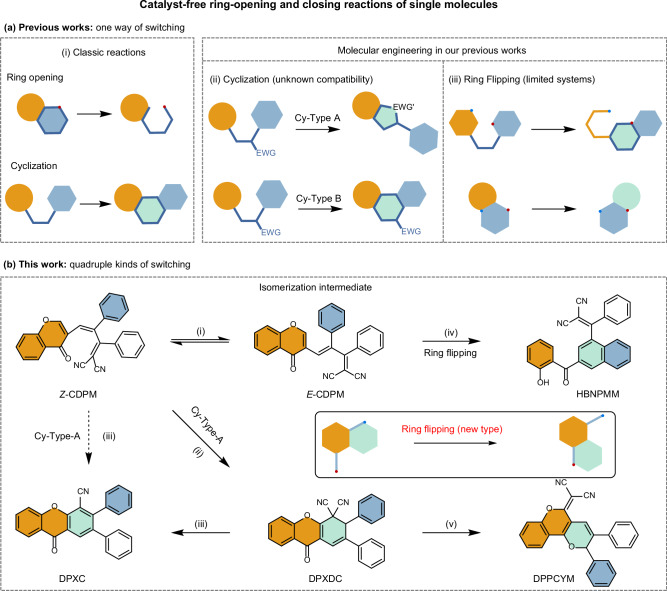
Design strategy of molecules with multiple responses and controls. **a** Conventional catalyst-free ring-opening and closing reactions in monomolecular systems and their characteristics. EWG electron-withdrawing group, Cy cyclization. **b** Schematic illustration of the thermal and photo reactions of *Z*-CDPM under different conditions: (i) annealing at 145 °C in C_2_D_2_Cl_4_ for 1 d; (ii) annealing at 200 °C in diphenyl ether for 2 h; (iii) annealing at 145 °C in silica gel for 1 d; (iv, v) UV irradiation under a 365 nm UV lamp (0.12 mW/cm^2^) at room temperature in C_2_D_2_Cl_4_ for 2 h.

Through analysis of classic reactions and current research works, an interesting story about compatibility and competitiveness in molecular design is revealed (Fig. 1a). For one thing, the cyclization pathway of polysubstituted ethylene derivatives with the EWG part can be divided into two types: (i) the Cy-Type A cyclization found in dihydroazulenes introduces the direct cyclization of the EWG substituent under annealing, showing a low energy barrier and stable cyclization products^36,37^; (ii) and the Cy-Type B cyclization process existing in stilbenes illustrates the cyclization of aromatic rings^18,19,38^, which usually require additional rearomatization process for stabilization and meanwhile UV irradiation to overcome the high energy barrier. Likely, the compatibility of these two stimulus patterns points to an important avenue in the pursuit of molecular versatility. For another thing, the ring-flipping processes involving both ring-closing and ring-opening reactions in molecules are reported^40,41^, which highlights the competitiveness among two ring-closing states obtained during the ring-reconstruction process. On the basis of extensive studies on EWG-substituted ethylene derivatives, two proper EWGs might be also incorporated into the molecule to achieve such a ring-flipping process. Therefore, if one molecule could undergo different types of cyclization and also ring-flipping processes to afford multiple ring-closing states, its multifunctionality could be anticipated. On the other hand, due to the excellent performance of aggregation-induced emission (AIE) luminogens in solid state^42–50^ and also *Z*/*E* isomerization-based responsive systems^51,52^, both the properties of AIE and stable *Z*/*E* isomeric intermediate state are further expected.

Herein, we designed and synthesized a simple AIEgen, namely (*Z*)−2-[3-(4-oxo-4H-chromen-3-yl)−1,2-diphenylallylidene]malononitrile and abbreviated as *Z*-CDPM (Fig. 1b), which exhibited six distinct switchable states. Under thermal treatment, *Z*-CDPM initially underwent *Z*/*E* isomerization to form *E*-CDPM, followed by Cy-Type A cyclizations to produce the isomerized product DPXDC and also its rearomatization product DPXC via an additional elimination process. While *Z*-CDPM displayed the ring-flipping process to give the photoarrangement product HBNPMM under UV irradiation, DPXDC followed another ring-flipping mechanism and generated DPPCYM with the assistance of the electron-withdrawing carbonyl group. The structures of all these six molecules were confirmed by the single-crystal X-ray diffraction technique. Their distinct photophysical properties in both solution and solid state were examined to confirm their potential in performing functional discolorations. Through experimental and theoretical analyses, the detailed mechanisms of these thermal and photo-switching processes were studied as well. Furthermore, dip-coated films of these multiresponsive luminogens (*Z*-CDPM and DPXDC) were fabricated to demonstrate the utility, including colored images and quick response (QR) codes, and an advanced encryption system. Thus, this work is of great significance for both fundemantal understanding and functional innovation in multifunctional systems.

## Results

### Photophysical properties and *Z*/*E* isomerization

**Fig. 2:**
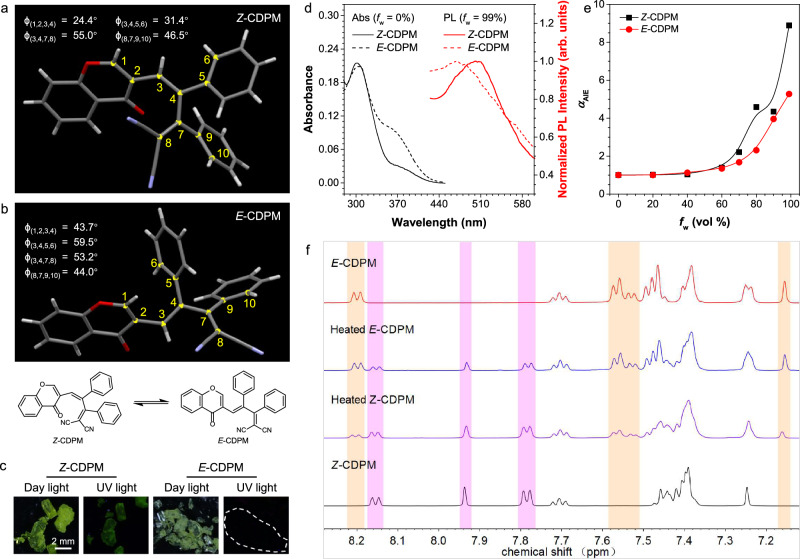
Photophysical properties and *Z*/*E* isomerization. Single crystal structures of **a** *Z-*CDPM and **b** *E-*CDPM. Insets: chemical structures and dihedral angles in the crystal structures. **c** Photographs of crystals of *Z-*CDPM and *E-*CDPM. Scale bar: 2 mm. **d** Absorption (Abs) and normalized photoluminescence (PL) spectra of *Z-*CDPM (solid line, 10 μM) and *E-*CDPM (dash line, 10 μM) in THF/water mixtures with different water fractions (*f*_w_). **e** Plots of *I*/*I*_0_ values (defined as α_AIE_) versus the *f*_w_ of *Z-*CDPM (black, 10 μM, *λ*_ex/em_ = 365/505 nm) and *E-*CDPM (red, 10 μM, *λ*_ex/em_ = 350/480 nm), where *I*_0_ = PL intensity in pure THF solution at the corresponding emission wavelength. **f** ^1^H NMR spectra of *Z-*CDPM and *E-*CDPM before and after heating at 145 °C in C_2_D_2_Cl_4_ for 1 d (500 MHz). *Z-*CDPM was highlighted in purple and *E-*CDPM was highlighted in orange.

*Z*-CDPM and *E*-CDPM were simultaneously synthesized via a facile two-step process (Supplementary Fig. 1), and successfully separated and purified one by one by column chromatography. Accordingly, the spectroscopical data of the intermediate and final products that conformed to their molecular structures were all acquired (Supplementary Figs. 2–10). By dissolving molecules in a mixed solvent of dichloromethane (DCM) and petroleum ether and followed by a slow evaporation process, the single crystals of *Z*-CDPM and *E*-CDPM were obtained as well. The results further revealed their twisted conformations (Fig. 2a and 2b and Supplementary Table 1). In particular, two dihedral angles (*ϕ*(1,2,3,4) and *ϕ*(3,4,5,6)) observed in *Z*-CDPM (24.4°, 31.4°) were found to be much smaller than those of *E*-CDPM (43.7°, 59.5°). Meanwhile, another two dihedral angles (*ϕ*(3,4,7,8) and *ϕ*(8,7,9,10)) appeared similar and large for both two molecules. Such staggered conformations indicated the effect of steric hindrance on molecular thermodynamic stability, as well as the simultaneous generation of two isomers (*Z*-CDPM and *E*-CDPM) in similar yields.

Following, the photophysical properties of *Z*-CDPM and *E*-CDPM were investigated. While the yellow crystals of *Z*-CDPM emitted dim greenish-yellow fluorescence, the pale-yellow crystals of *E*-CDPM were almost non-emissive (Fig. 2c and Supplementary Fig. 11). In solution state, a main absorption peak in the visible region of 310 nm was detected for both *Z*-CDPM and *E*-CDPM (Fig. 2d) owning to their poor conjugation^40,53,54^. Given the poor solubility of organic molecules in water, water was chosen as the poor solvent and added to their THF solutions to promote aggregate formation. Then, gradually enhanced fluorescence was detected for both *Z*-CDPM and *E*-CDPM (Fig. 2e and Supplementary Fig. 12). At a water fraction (*f*_w_) of 99%, the fluorescence intensity of *Z*-CDPM and *E*-CDPM respectively was nine-fold and five-fold higher than that in pure THF solution, which confirmed their AIE properties. But notably, *Z*-CDPM emitted weakly at 505 nm in the THF/water mixture (*f*_w_ = 99%), while the photoluminescence (PL) of *E*-CDPM peaked at 480 nm instead because of its more twisted conformation. Given the AIE and different emission properties of two isomers with twisted conformations, it might shed light on the design of isomerization-based responsive luminogens^51,52^.

To verify the *Z*/*E* isomerization process under annealing, a deuterium reagent C_2_D_2_Cl_4_ with a high boiling point (b.p.) was chosen to prepare the stock solutions for dynamic ^1^H NMR analysis. Because of their different conformations, different ^1^H NMR spectral patterns were detected for *Z*-CDPM and *E*-CDPM. Also, some peaks could be set as references for feasible investigation of the isomerization. As depicted in Fig. 2f and Supplementary Fig. 13, the specific peaks of *E*-CDPM at *δ* 8.21-8.19, 7.58-7.52, and 7.16 emerged in the heated solution of *Z*-CDPM. The ratio between the *Z*-isomer and the *E*-isomer was calculated to be 64:34 according to the related integral peak. On the other hand, a similar phenomenon reappeared in the stock solution of *E*-CDPM, and a ratio of 37:60 was obtained. The reaction rate constant for the isomerization of *Z*-CDPM and *E*-CDPM was measured respectively (0.151 and 0.181 mmol·L^-1^·h^-1^), which further illustrated the relatively high stability of *Z*-CDPM (Supplementary Fig. 14). To sum up, the similar thermodynamic stability of two isomers induced a thermal *Z*/*E* isomerization process and leads to a near-equilibrium distribution for CDPM, while its utility in modulating luminescent output is limited because of the inability to significantly accumulate one isomer.

### Thermal cyclization

**Fig. 3:**
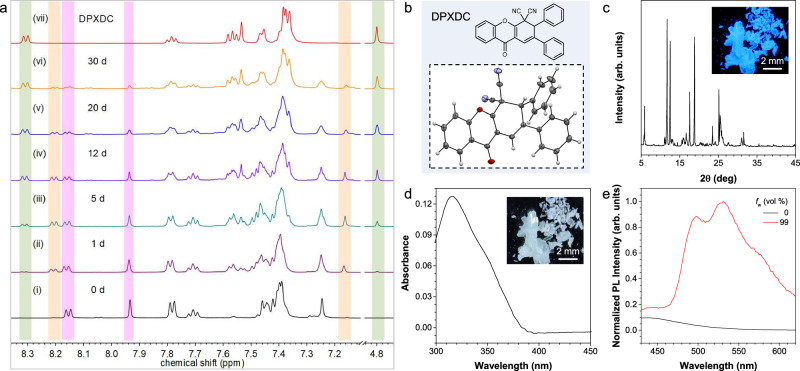
Thermal cyclization of *Z-*CDPM. **a** ^1^H NMR spectra of as prepared *Z-*CDPM before and after heating at 145 °C in C_2_D_2_Cl_4_ (500 MHz) for different times (1, 5, 12, 20, and 30 d). *Z-*CDPM was highlighted in purple, *E-*CDPM was highlighted in orange, and DPXDC was highlighted in green. **b** Chemical and single-crystal structures of DPXDC. **c** X-ray diffraction (XRD) diffractograms of the crystal powder of DPXDC. Inset: photograph of crystals of DPXDC taken under UV illumination. Scale bar: 2 mm. **d** Absorption spectra of DPXDC (10 μM) in THF. Inset: photograph of crystals of DPXDC taken under day light. Scale bar: 2 mm. **e** Photoluminescence (PL) spectra of DPXDC (10 μM) in THF/water mixtures with different water fractions (*f*_w_, 0 and 99%). *λ*_ex_ = 365 nm.

Then, the heating process was prolonged to test the hypothesis of Cy-Type A thermal cyclization in *Z*-CDPM. As depicted in Fig. 3a, not only similar NMR peak intensities were detected for *Z*-CDPM and *E*-CDPM on day 5, but also different peaks at *δ* 8.31-8.30 and 4.80 emerged. Followed, a quick test where *Z*-CDPM was dissolved in diphenyl ether (b.p. 258 °C) and heated at 200 °C for 2 h (Supplementary Fig. 15) was conducted. A cyclized product of DPXDC was later isolated in a reaction yield of 87% and identified by single-crystal X-ray diffraction (Fig. 3b and Supplementary Table 2). Satisfactory spectroscopical results corresponding to its molecular structure were obtained as well (Supplementary Figs. 16–18), validating the Cy-Type A thermal cyclization of *Z*-CDPM in solution.

The dynamic ^1^H NMR analysis depicted the slow but efficient conversion of *Z*-CDPM to DPXDC in C_2_D_2_Cl_4_. As depicted in Fig. 3a, the NMR peaks of DPXDC at δ 8.31-8.30 and 4.80 gradually intensified and eventually resulted in a ^1^H NMR spectrum compared with that of DPXDC after 30 days of heating. Since the peaks in the aromatic range were well matched with those of *Z*/*E*-CDPM or DPXDC, a controllable thermal cyclization process and the sole generation of DPXDC were demonstrated. However, a fast and efficient generation of DPXDC at 200 °C was observed in the synthetic procedure otherwise. Curiously, analysis by differential scanning calorimeter (DSC) in the range from 0 °C to 200 °C was conducted to get a deeper insight. The DSC curve of *Z*-CDPM exhibited a remarkable peak at 178 °C, suggesting an exothermic thermal cyclization process (Supplementary Fig. 19). As the reaction energy (∆G = 8.09 kcal·mol⁻¹) of *Z*-CDPM was similar to that of *E*-CDPM (∆G = 7.43 kcal·mol⁻¹), the comparable thermodynamic stability of these two isomers, *Z*- and *E*-CDPM, was confirmed again. Together with the similar results observed in the dynamic ^1^H NMR analysis of *E*-CDPM (Supplementary Figs. 20 and 21), the independence and robustness of the thermal cyclization of *Z*/*E* CDPM were validated.

Subsequently, the photophysical properties of DPXDC were investigated. As depicted in Figs. 3c, 3d, the white crystal of DPXDC showed moderate blue emission under UV irradiation. Meanwhile, a main absorption peak at 315 nm and a weak emission peaked at 450 nm were observed in its THF solution (Figs. 3d, 3e). When water was chosen as a poor solvent and added to the THF solution to promote aggregate formation, an enhancement in the fluorescence intensity (22 times at *f*_w_ = 99%, 495 nm) was then observed for DPXDC, as well as the gradually red-shifted emission peak (Supplementary Fig. 22). This indicated the AIE and specific emission properties of DPXDC. Taken together the photophysical properties of DPXDC and the high efficiency of the thermal cyclization in solution, a thermochromic motif of *Z*-CDPM could be coined.

### Thermal elimination

**Fig. 4:**
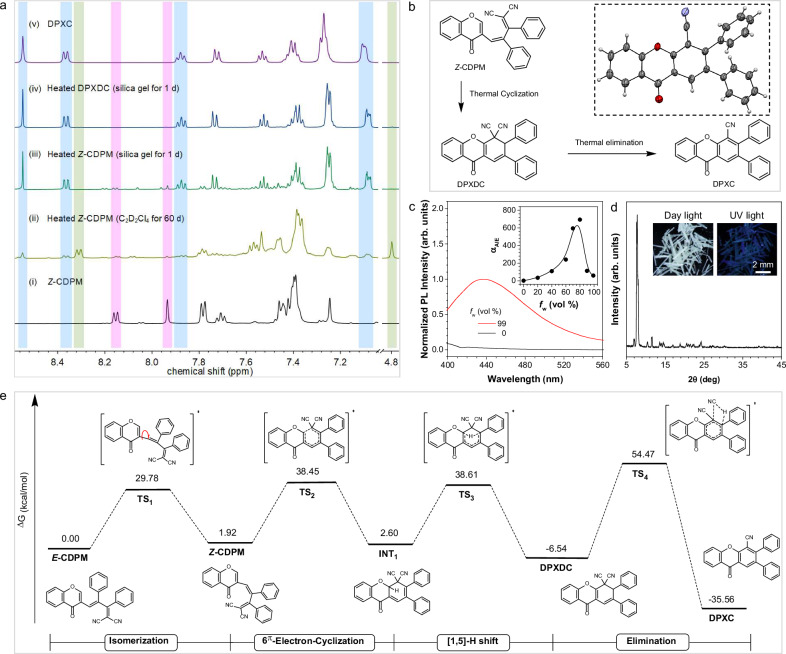
Thermal elimination of *Z-*CDPM and theoretical calculations. **a** ^1^H NMR spectra of *Z-*CDPM before and after heating at 145 °C under different conditions (500 MHz). *Z-*CDPM was highlighted in purple, DPXDC was highlighted in green, and DPXC was highlighted in blue. **b** Proposed reaction routes for the formation of DPXC from *Z*-CDPM and DPXDC. **c** Photoluminescence (PL) spectra of *Z*-DPXC in THF/water mixtures (10 μM). Inset: plots of *I*/*I*_0_ value versus the water fractions (*f*_w_) of THF/water mixtures of DPXC, where *I*_0_ = PL intensity in pure THF solution. *λ*_ex/em_ = 355/440 nm. **d** X-ray diffraction diffractograms of the crystal powder of DPXC. Insets: photographs of crystals of DPXC. Scale bar: 2 mm. **e** Energy profile for the thermal reactions of *Z-*CDPM calculated at the M06-2X/6-31 G(d,p) level with solvent correction. TS transition state, INT intermediate.

Unexpectedly, when silica gel was initially added to the DCM solution of *Z*-CDPM and then heated to test its thermochromic behavior in the solid state, a completely different ^1^H NMR spectrum was obtained (Fig. 4a). Eventually, no signal of DPXDC or *Z*-CDPM could be detected at all. To unlock this mystery, the newly formed compound was followed isolated and purified by column chromatography. A molecular structure of DPXC was confirmed according to the single-crystal data (Fig. 4b, Supplementary Fig. 23 and Table 3). Satisfactory spectroscopical results corresponding to its molecular structure (Supplementary Figs. 24–26), including the ^1^H NMR spectrum compared with that of the final heated sample of *Z*-CDPM were obtained as well (Fig. 4a). Considering the appearance of characteristic NMR peaks of DPXC at δ 8.53, 8.35-8.34, 7.87-7.84 and 7.09-7.08 in the dynamic ^1^H NMR analysis (Supplementary Fig. 27), a feasible Cy-Type A cyclization process of *Z*-CDPM to produce DPXC via thermal elimination when dispersed in silica gel could be demonstrated. On the other hand, DPXC absorbed at 355 nm and emitted weakly in THF, and the obviously enhanced emission at 440 nm in a THF/water mixture (60 times, *f*_w_ = 99%) validated its AIE property (Fig. 4c and Supplementary Figs. 28 and 29). Besides, a weak blue emission was detected in the white crystal of DPXC (Fig. 4d). Combined with the thermochromic behavior of *Z*-CDPM in the TLC plate (Supplementary Fig. 30), thus *Z*-CDPM was capable of performing triple chemical switching under thermal treatment but only one obvious functional discoloration in the solid state through the elimination process. But notably, it also validated the relative independent maintenance of the thermal switching states.

Under similar reaction conditions, DPXC was generated from DPXDC in the solid state (Fig. 4a) as well. Also, the enrichment of DPXDC after 30 days of heating appeared to accelerate the formation of DPXC in solution (Supplementary Fig. 31), making DPXDC a promising intermediate for thermal elimination. On the other hand, however, the thermal elimination process of *Z*-CDPM remained negligible even after 60 days of heating in solution. Also, no significant changes could be found in the ^1^H NMR spectrum of the heated crystal powder of *Z*-CDPM (Supplementary Fig. 32). According to the difference in molecular structure, a volatile molecule HCN should be generated in the thermal elimination, just as the elimination process of dihydroazulenes^36^. Possibly, the volatilization of HCN was effectively accelerated by dispersing *Z*-CDPM or DPXDC into silica gel, which eventually promoted the thermal elimination process. In combination with the results obtained in the acidulated solution and the alumina medium (Supplementary Figs. 32 and 33), a possible reaction pathway for the formation of DPXC was thus proposed and shown in Fig. 4b.

Subsequently, thermogravimetric analysis were performed to investigate the thermal stability of these molecules. As shown in supplementary Fig. 34, the thermogravimetric differential (DTG) curves of *Z*-CDPM and DPXDC were found both stable at temperatures below 200 °C, which was consistent with the results of DSC and NMR titration experiment showing the generation and enrichment of DPXDC at low temperatures. Meanwhile, a maximum peak at about 390 °C was detected for *Z*-CDPM, DPXDC and DPXC, indicating their high decomposition temperature. As *Z*-CDPM and DPXDC both showed a small peak around 300 °C, and similar results were found in the DTG curve of the former isomer *E*-CDPM as well, a high transition temperature for the thermal elimination process could be demonstrated. Herein, the thermal elimination process of the solid powder of *Z*/*E*-CDPM and DPXDC required an high temperature and occurred simultaneously with the thermal degradation process.

Later, density functional theory (DFT) calculation was carried out to illustrate the reaction pathways. As depicted in Fig. 4e, the initial isomerization of *E*-CDPM formed the metastable product *Z*-CDPM (1.92 kcal/mol). Although DFT calculations at the M06-2X/6-31 G(d,p) level without solvent correction suggest a 1.92 kcal/mol free energy difference between *Z*- and *E*-CDPM (Supplementary Fig. 35), this value falls within the typical error margin of DFT methods and may not reliably reflect the experimental isomeric distribution, especially under non-equilibrium conditions. Following, the 6π-electron-cyclization led to the formation of another metastable ring-closed intermediate called INT1 (2.60 kcal/mol), which further underwent [1,5]-H shift to give the stable thermal cyclization product DPXDC with a low energy of −6.54 kcal/mol. While the relatively high energy barrier (36.53 and 36.01 Kcal/mol) confirmed the requirement of intense heating conditions for the Cy-Type A cyclization of *Z*/*E*-CDPM, the low energy of INT1 further validated its feasibility. Finally, the elimination process took place and resulted in the generation of a stable aromatization product of DPXC (-35.56 Kcal/mol) and a by-product of HCN. Since INT1 needed to overcome a higher energy barrier of 80.56 Kcal/mol than that of DPXDC (61.01 Kcal/mol) to generate DPXC, theoretically it would get over a lower energy barrier of [1,5]-H shift (36.01 Kcal/mol) to form DPXDC first (Supplementary Fig. 36). Therefore, a cyclization product DPXDC-mediated elimination for *Z*/*E*-CDPM could be proposed.

It is worth noting that while the partial interconversion between *Z*- and *E*-CDPM was observed experimentally, the presence of subsequent thermal reactions (e.g., formation of DPXDC) complicated the accurate extraction of an equilibrium Z/E ratio. Therefore, a reliable experimental estimation of ΔG was currently not feasible without kinetic deconvolution. Also, our data suggested that E-CDPM is a key intermediate in route to DPXDC, but the thermal conversion of Z-CDPM may proceed via both sequential and branching pathways (Supplementary Figs. 35–38). Moreover, the solvent-corrected energy profiles at the M06-2X/6-31 G(d,p) level show consistent trends with the original gas-phase results (Supplementary Figs. 35 and 36), as well as the energy profiles calculated at the TPSS/6-31 G(d,p) level without solvent correction (Supplementary Fig. 37). Herein, it reinforced the robustness of the proposed mechanisms. Conceivably, this mechanistic insight of the thermal reactions not only pointed out the transformation pathways of Z-CDPM between different thermal-switchable states but also provided possibilities for molecular engineering.

### Photoarrangement of *Z*-CDPM

**Fig. 5:**
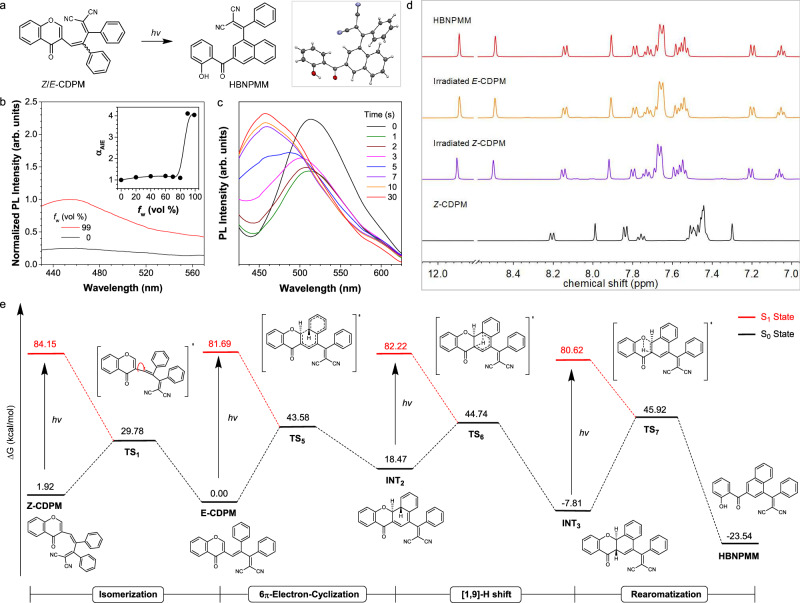
Photoarrangement of Z-CDPM. **a** Photoarrangement of *Z*/*E*-CDPM and single crystal structures of HBNPMM. **b** Photoluminescence (PL) spectra of HBNPMM (10 μM) in THF/water mixtures. Inset: plots of *I*/*I*_0_ value versus water fractions (*f*_w_) of THF/water mixtures of HBNPMM, where *I*_0_ = PL intensity in pure THF solution. λ_ex/em_ = 365/460 nm. **c** PL spectral change of *Z-*CDPM before and after UV irradiation at 365 nm for different times (1, 2, 3, 5, 7, 10, and 30 s). λ_ex_ = 365 nm **d** ^1^H NMR spectra of the photoarrangement of *Z/E-*CDPM in C_2_D_2_Cl_4_ (500 MHz). *Z-*CDPM (red line) and *E*-CDPM (orange line) were irradiated by 365 nm UV irradiation from a 365 hand-hold UV lamp for 30 min, and the black line was HBNPMM. **e** Energy profile for the photoarrangement of *Z-*CDPM calculated at the M06-2X/6-31 G(d,p) level with solvent correction. TS transition state, INT intermediate.

Photochromic materials has been attractive in recent years owning to the benign nature, availability and spatial and temporal control of light^55–61^, including numerous research on functional motifs^62–64^, control models^65–69^ and applications^70–79^. Due to the presence of the moiety of arylvinyl-substituted chromone, *Z/E*-CDPM should undergo a ring-flipping process under UV irradiation to form an isomer called HBNPMM (Fig. 5a)^40,80^. Then, the chloroform solution of *Z*-CDPM was prepared and irradiated using a 365 nm UV lamp. As expected, the reaction product HBNPMM was successfully isolated and well characterized (Supplementary Figs. 39–42 and Table 4), including the single crystal data to support its molecular structure (Fig. 5a). HBNPMM absorbed at 303 nm in THF, and exhibited AIE property given the gradually enhanced fluorescence at 460 nm upon water addition (4 times at *f*_w_ = 99%, Fig. 5b and Supplementary Figs. 43 and 44). Also, a faint blue emission was observed for its light-yellow crystal (Supplementary Fig. 45), illustrating the photophysical properties of HBNPMM.

Upon UV irradiation, the emission of *Z*-CDPM in solution was blue-shifted and reached a terminal point of 460 nm after 10 s, resulting in a spectrum similar to that of HBNPMM (Fig. 5b and 5c). As the fluorescence intensity almost reached the plateau at the same time, a fast and traceable photoarrangement process was confirmed. Besides, a continuous enhancement in fluorescence intensity was detected for the weakly emissive *E*-CDPM during UV irradiation at 365 nm (Supplementary Fig. 46). Taken together of the photochromic behavior of *Z*/*E*-CDPM in the solution and TLC plate under UV irradiation (Supplementary Figs. 47 and 48), the capability of *Z*/*E*-CDPM in performing on-demand discoloration in real applications through photoarrangement was confirmed.

Subsequently, the dynamic NMR analysis (Fig. 5d) demonstrated a highly efficient photoarrangement of *Z*/*E*-CDPM and the formation of solely HBNPMM in solution. According to DFT calculation results (Fig. 5e), UV irradiation of *Z*-CDPM initially led to the formation of *E*-CDPM. Then, a 6π-electron-cyclization took place to generate the unstable cyclized intermediate (INT2) with a high energy of 18.47 kcal/mol, attributed to the destroyed aromatic conjugation in the benzene ring. Herein, a [1,9]-H shift process was further followed to generate a relatively stable intermediate (INT3) with a low energy of −7.81 kcal/mol. As previously reported, a decrease in fluorescence intensity was also detected in the initial irradiation period (Fig. 5c), indicating the enrichment of the non-emissive metastable intermediate product INT3^38^. Eventually, the rearomatization led to the generation of the stable product HBNPMM (-23.54 Kcal/mol) with a better molecular conjugation. Considering the high energy barriers for these processes, UV irradiation was necessary to excite the molecule to overcome these energy barriers. The solvent-corrected energy profiles show consistent trends with the original gas-phase results, thereby reinforcing the robustness of the proposed mechanisms (Supplementary Fig. 49). Thus, the feasibility of arylvinyl substituted chromone as a photoactivatable motif and also the mechanism differentiation strategy to maintain the switching models of *Z*-CDPM was demonstrated.

### Photoarrangement of DPXDC

**Fig. 6:**
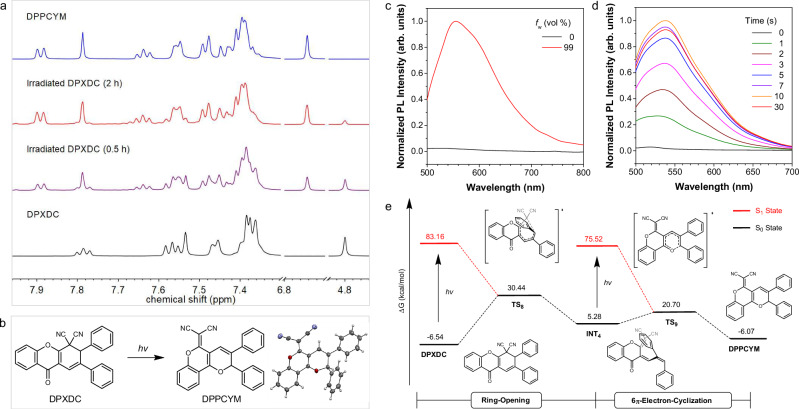
Photoarrangement of DPXDC. **a** Changes in ^1^H NMR spectrum of DPXDC in C_2_D_2_Cl_4_ during the photoarrangement process (500 MHz). **b** Photoarrangement of DPXDC and single crystal structure of HBNPMM. **c** Photoluminescence (PL) spectra of HBNPMM (10 μM) in THF/water mixtures with different water fractions (*f*_w_). *λ*_ex_ = 440 nm. **d** PL spectral of DPXDC before and after UV irradiation at 365 nm for different times (1, 2, 3, 5, 7,10, and 30 s). *λ*_ex_ = 440 nm. **e** Energy profile for the photoarrangement of DPXDC calculated at the M06-2X/6-31 G(d,p) level with solvent correction. TS transition state, INT intermediate.

To expand the types of photoactivatable motifs and verify the ring-flipping hypothesis in the molecule with double EWGs, the C_2_D_2_Cl_4_ solution of DPXDC was prepared and irradiated using a 365 nm UV lamp. As shown in Fig. 6a, different peaks at δ 7.90-7.88, 7.65-7.62, and 6.74 emerged and some original peaks of DPXDC disappeared 2 h later. Under similar conditions, the molecular DPPCYM was synthesized and identified according to the spectroscopical results and single-crystal X-ray diffraction data (Fig. 6b, Supplementary Figs. 50–53 and Table 5). Due to the similarity in the spectral pattern of the irradiated solution of DPXDC and DPPCYM (Fig. 6a), a photoarrangement of DPXDC and the sole generation of DPPCYM under UV irradiation could be verified.

Following, the photophysical properties of DPPCYM and the fluorescence response of DPXDC to UV irradiation were investigated. DPPCYM displayed two main absorption peaks at 347 nm and 440 nm in THF, and its orange single crystal emitted strong yellow-green fluorescence (Supplementary Figs. 54 and 55). Upon water addition, an enhancement in fluorescence intensity at around 550 nm was observed (47 times at *f*_w_ = 99%, Fig. 6c and Supplementary Fig. 56), confirming the AIE property of DPPCYM. Considering the different photophysical properties of DPXDC and DPPCYM, the feasibility of performing visualized ring-flipping process in a single-luminogen system was demonstrated^40,41^. On the other hand, a gradually enhanced emission was detected for the UV irradiated sample of DPXDC in THF/water mixture with a *f*_w_ of 99% (Fig. 6d). While DPXDC is AIE-active, the fluorescence intensity increased 68 times at 550 nm and almost reached a plateau after 10 s of irradiation, illustrating a fast and traceable photoarrangement process. Previously, a long-wavelength peak at around 535 nm was detected for DPXDC at *f*_w_ = 90% and 99% under 365 nm excitation (Fig. 3e), which was similar to that of DPPCYM. Combined with the fast photoarrangement of DPXDC in solution, this signal at 535 nm would be attributed to the generation of DPPCYM upon UV excitation. Furthermore, the photochromic behavior of DPXDC in the TLC plate was demonstrated (Supplementary Fig. 57), validating the potential of DPXDC for conducting fast and UV light-controlled coloration in real applications.

Later, DFT calculations were carried out to illustrate the reaction pathway of the photoarrangement of DPXDC. As depicted in Fig. 6e, two processes were involved in the photoarrangement of DPXDC: one was a ring-opening process with the formation of an unstable intermediate product INT4 (5.28 kcal/mol); the other was a followed 6π-electron-cyclization to generate the final product DPPCYM with a low energy of -6.07 kcal/mol. For one thing, the energy barriers for these processes were high and thus UV irradiation was required to trigger their occurrence. For another thing, the initial ring-opening process (Fig. 6e) resulted in an unstable intermediate (INT4). Thus, the signals of INT4 were almost missing in the dynamic ^1^H NMR spectra (Fig. 6a). In addition, the consistent trends in solvent-corrected energy profiles further validated the robustness of the proposed mechanisms (Supplementary Fig. 58), suggesting a photoactivatable motif of DPXDC through the ring-flipping process. While direct excited-state dynamics simulations were not performed due to system size and computational cost, the mechanistic plausibility was assessed via TD-DFT and potential energy surface analysis, which captured the key trends and enabled qualitative rationalization of the photoresponses. Notably, compared with classic stilbene-based, diarylethene, or spiropyran systems, the high switching efficiency and irreversibility of the photoarrangement and the multiresponsiveness of *Z*-CDPM would be desirable for functional innovation in photoactivatable systems, which complement classical systems by enabling multi-output logic and high-level data encoding applications.

### Applications for information encryption

**Fig. 7:**
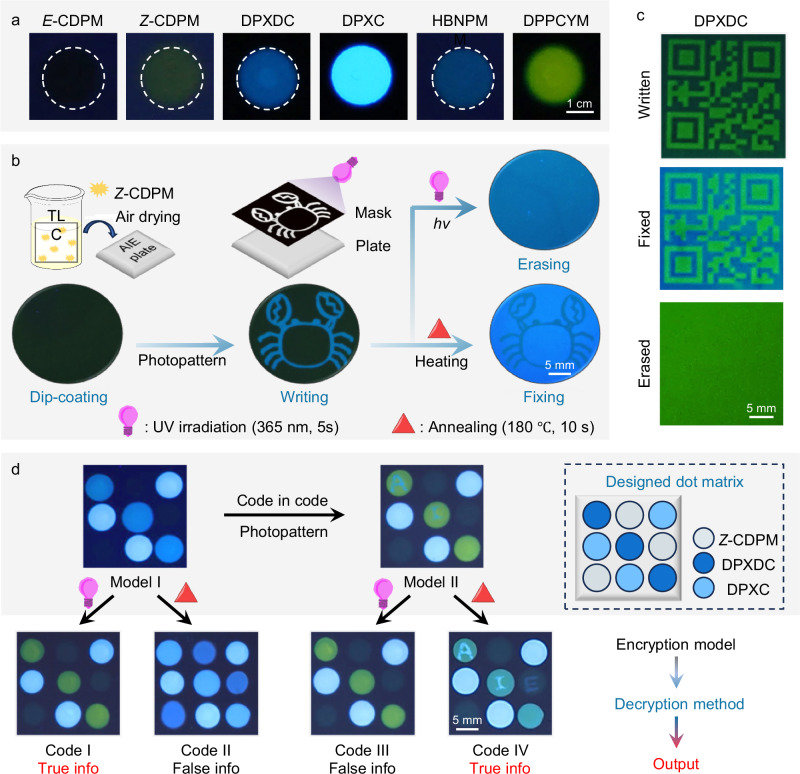
Applications for information encryption. **a** Fluorescent images of different AIEgens on a thin-layer chromatography (TLC) plate. Scale bar: 1 cm. **b** Schematic illustration of the dip-coating of *Z*-CDPM on a TLC plate and the following writing, fixing, and erasing process through UV irradiation (10 s) or heating (200 °C for 10 s), and the corresponding fluorescent images. Scale bar: 5 mm. **c** Photographs of the developed colored “quick response code” of DPXDC. Scale bar: 5 mm. **d** Demonstration of a multicolor encryption system dual encryption models. The light irradiation was conducted under a UV lamp (0.12 mW/cm^2^) at room temperature to trigger the photoarrangement of *Z*-CDPM and/or DPXDC, and the annealing procedure was performed to generate DPXC from *Z*-CDPM and/or DPXDC. Scale bar: 5 mm.

Due to the multiresponsive behaviors of *Z*-CDPM, a general strategy for constructing multifunctional systems was proposed. Initially, the different emission characteristics of these six AIEgens on the TLC plate were checked (Fig. 7a). It was worth noting that although the quantum yield of these compounds was not particularly high as compared with other reported AIEgens, the high conversion efficience and multiple switchable states would make them ideal candidates for functional systems (Supplementary Figs. 59, 60 and Tables 6, 7). Then, an plate of *Z*-CDPM on solid support (AIE plate) was obtained by dip-coating the thin-layer chromatography (TLC) plate into its DCM solution and followed by air-drying process (Fig. 7b). Based on the photochromic behavior of *Z*-CDPM, a photopattern with a homemade mask of a “crab” image was carried out and realized the noncontact “writing” process^37,40,41,81^. As expected, the blue-green-emissive “crab” image was clearly displayed in the plate owning to the photoconversion of *Z*-CDPM into HBNPMM, while the surroundings appeared non-emissive as the initial template. Later, when the “fixing” process was performed by heating the plate at 200 °C for 30 s, strong blue emission emerged in the surrounding area of the “crab” image, indicating the thermal elimination process of *Z*-CDPM and the formation of DPXC. Prior to this, the good thermal stability of HBNPMM and DPPCYM was also checked and confirmed (Supplementary Fig. 61). Eventually, the erasure process was realized by simply irradiating the written plate, and the “crab” was totally erased and resulted in the blue-emissive plate. In a word, *Z*-CDPM realized in-situ fabricating multicolor images in a single AIEgen system.

Multiple coloration, on the other hand, has great potential for applications like information encryption^82,83^. Since the thermal elimination and photoarrangement of *Z*-CDPM occurred at the same position of the chromone ring, obviously, it was possible to manipulate these two processes according to the sequence. Then, a written QR code was manufactured through photopatterning on an plate on solid support. For one thing, the written QR code could be read under UV illumination, but disappeared when increasing the power of the UV light source (Supplementary Fig. 62). Such a traceless and easy-operation erasure process derived from the fast photoarrangement of *Z*-CDPM was undoubtedly conducive to information security. For another thing, a colored fixed QR code with blue and blue-green fluorescence could be subsequently obtained through heating and further read repeatedly under UV irradiation (Supplementary Figs. 62 and 63), demonstrating its high potential for long-term information preservation. Likewise, the thermal elimination and photoarrangement of DPXDC also occurred at the same site of the newly-formed six-membered ring, showing similar results for the multicolored QR code (Fig. 7c). Because of the high conversion efficiency and irreversibility of both the thermal and photo switching process, DPXDC could conduct one-way discoloration in information encryption systems with long-term stability (Supplementary Fig. 64).

At last, an advanced information encryption system with the “dual encryption-decryption model” was proposed. As a preliminary proof-of-concept, a dot matrix was designed to illustrate the information storage procedure. In general, the different photoreactivity of *Z*-CDPM and DPXDC would be utilized to conduct on-demand discoloration in artificial systems, while their similar thermal elimination supported the erasure process. Based on this spot, we manufactured the initial matrix (mode I) to support the conventional encryption model. As shown in Fig. 7d, the true information (code I) with good differentiation and the right color array could be obtained through the UV irradiation process, while erased and false information was found in the heated matrix (code II). On the other hand, due to the remote or noncontact coloration model of photochromic materials, another matrix (model II) was further developed through a photopattern process for realizing the “code in code” encryption model. Specifically, three letters “A”, “I” and “E” were chosen as another kind of secret information and stored in the matrix (model II). Then, the true information with both letters and the right color array was observed in the heated matrix (code IV) instead, while UV irradiation resulted in an erased matrix (code III) with a piece of false information. Notably, “opposite decryption and erasure processes” existed in these two encryption-decryption models. Together with the specificity of thermal elimination and photoarrangement process, the high value of this information encryption system could be demonstrated. Thus, these AIEgens (*Z*-CDPM and DPXDC) not only demonstrated their great potential in the development of multifunctional systems but also paved a good approach to achieve high-level security information encryption systems.

## Discussion

In this work, a quintuple responsive and controllable chromone-based AIEgen called *Z*-CDPM which showed six distinct switching states was designed and synthesized. This molecule exhibited efficient, quintuple, and controllable thermal/photo reaction operated in the mechanisms of (i) reversible *Z*/*E* isomerization under thermal treatment; (ii) thermal cyclization in solution; (iii) thermal elimination when dispersed in silica gel; (iv) photoarrangement; (v) thermal cyclization product DPXDC-mediated photoarrangement. Subsequently, single crystals for these six molecules were all obtained to verify the chemical structures, while dynamic NMR analysis and theoretical calculations were conducted to further elucidate the detailed reaction mechanisms. Moreover, experimental results demonstrated the self-reporting capability of each reaction. Leveraging the multiresponsiveness, self-reporting properties, and controllability of *Z*-CDPM and DPXDC, a quick response code with erasable and rewriteable capabilities, and an advanced multicolor information encryption system were developed. To our knowledge, *Z*-CDPM represents a rare example of a molecule exhibiting quintuple intelligent dynamic coloration properties. This work not only provides effective strategies for constructing multifunctional luminogens but also introduces quintuple-responsive luminogen motifs to advance information encryption technology.

## Methods

### Apparatus and reagents

^1^H NMR (500 MHz) and ^13^C NMR (125 MHz) spectra were recorded on a Bruker Avance 500 MHz NMR spectrometer. High-resolution mass spectra (HRMS) were recorded on a GCT premier CAB048 mass spectrometer operated in a MALDI-TOF mode. Ultraviolet-visible (UV-vis) spectra were measured on a Lambda 365 spectrophotometer. Fluorescence spectra were measured on an Edinburgh FLS1000 spectrofluorometer. Single crystal data was collected on a Bruker Smart APEXII CCD diffractometer using graphite monochromated Cu Kα radiation (*λ* = 1.54178 Å). The differential scanning calorimetry (DSC) measurements were conducted on a TA Instruments DSC Q1000 at a heating rate of 10 °C/min under nitrogen. The thermogravimetric analysis (TGA) was conducted on a Mettler Toledo TGA2 Thermogravimetric Analyzer at a heating rate of 10 °C/min under nitrogen. A UV lamp (20 mW/cm^2^) was used as light irradiation source for all investigations.

All the chemicals were used as received without further purification unless otherwise specified. All the chemicals were purchased from Sigma-Aldrich Corp. and J&K Chemical Ltd. All the solvents were purchased from VWR Chemicals Corp. Anhydrous THF was used for fluorescence property investigation. Deionized water was used throughout this study.

### Computational details

All structures were optimized using the M06-2X functional and the 6–31 G(d,p) basis set for all atoms, including Grimme’s DFT-D3 empirical dispersion correction with the original damping function. Analytical frequency calculations at the same level of theory identified all stationary points as either intermediates (no imaginary frequency) or transition states (one imaginary frequency) and provided thermal corrections to the free energy. To refine the calculated energies, single-point calculations with the larger 6–311 + + G(d,p) basis set were performed on these optimized structures. The reported free energies include the electronic energy from single-point calculations, the Gibbs free energy correction obtained from vibrational analysis in the gas phase at the experimental reaction temperature (145 °C), and the DFT-D3 empirical dispersion correction. The energy of the excited state S_1_ was obtained from single-point TD-DFT calculations on the ground-state structures at the M06-2X/6-311 + + G(d,p) level. All DFT geometry optimizations and DFT/TD-DFT single-point calculations were performed using the Gaussian 16, Revision A.03.

## Supplementary information

Supplementary Information

Description of Additional Supplementary Files

Supplementary Data 1

Transparent Peer Review file

## Data Availability

All the data supporting the findings in this work are available within the manuscript and Supplementary Information file, and available from the corresponding authors upon request. Coordinates of the optimized structures are provided in the supplementary data file. The X-ray crystallographic coordinates for structures reported in this work have been deposited at the Cambridge Crystallographic Data Centre (CCDC) under deposition numbers of 2357788 (*Z*-CDPM), 2357790 (*E*-CDPM), 2357744 (DPXDC), 2357785 (DPXC), 2357787 (HBNPMM) and 2357792 (DPPCYM). These data can be obtained free of charge from The Cambridge Crystallographic Data Centre via www.ccdc.cam.ac.uk/data_request/cif.
