# Supplementary material for: A chameleon AIEgen exhibiting six distinct yet tunable thermal and photoswitchable states
Source: Nat Commun. 2025 Jul 9;16:6312. doi: 10.1038/s41467-025-61717-x (PMC12238559; doi:10.1038/s41467-025-61717-x)
Supplement: Supplementary file 1 — Supplementary Information [file 41467_2025_61717_MOESM1_ESM.pdf]

Supplementary Information

A chameleon AIEgen exhibiting six distinct yet tunable thermal and photoswitchable states

Xinyuan He,[1] Baochuan Hu,[2] Xin Wang,[3] Xing Feng,[4] Xinyuan Wang,[1] Xinmeng Chen,[1] Jianwei Sun,[1] Jacky W. Y. Lam,[1,*] Lianrui Hu,[2,*] and Ben Zhong Tang[1,5,6,*]

[1] Department of Chemistry, and the Hong Kong Branch of Chinese National Engineering Research Center for Tissue Restoration and Reconstruction, The Hong Kong University of Science and Technology, Clear Water Bay, Kowloon, Hong Kong, China

[2] Shanghai Key Laboratory of Green Chemistry and Chemical Processes, Shanghai Frontiers Science Center of Molecule Intelligent Syntheses, School of Chemistry and Molecular Engineering, East China Normal University, Shanghai, 200062, China

[3] College of Chemistry and Chemical Engineering, Inner Mongolia Key Laboratory of Fine Organic Synthesis, Institutes of Biomedical Sciences, Inner Mongolia University, Hohhot, 010021, China

[4] School of Material and Energy, Guangdong University of Technology, Guangzhou 510006, China

[5] Center for Aggregation-Induced Emission, South China University of Technology, Guangzhou, 510640, China

[6] School of Science and Engineering, Shenzhen Institute of Aggregate Science and Technology, The Chinese University of Hong Kong, Shenzhen, Guangdong, 518172, China

Corresponding emails: chjacky@ust.hk (J. W. Y. Lam); lrhu@chem.ecnu.edu.cn (L. Hu); tangbenz@ust.hk (B. Z. Tang).

Table of Contents

Figures and Tables ……………………………………………………………….……5

Supplementary Fig. 1…...………………………………………………….………........5

Supplementary Fig. 2.…………………………………..…………….………........6

Supplementary Fig. 3………………………………………………….......................…6

Supplementary Fig. 4…………………………………………….……..........………6

Supplementary Fig. 5…………………………………………………...........………7

Supplementary Fig. 6…………………………………………………...........………7

Supplementary Fig. 7……………………………..…………………...........………7

Supplementary Fig. 8…………………………………………………...........………8

Supplementary Fig. 9……………………………..…………………...........……...8

Supplementary Fig. 10…………………………………………………..........………8

Supplementary Table 1…..……...…………………………………….…………......9

Supplementary Fig. 11…………………………………………………..........………9

Supplementary Fig. 12…………………………………………………..........………10

Supplementary Fig. 13…………………………………………………..........………10

Supplementary Fig. 14…………………………………………………..........………10

Supplementary Fig. 15…………………………………………………..........………11

Supplementary Table 2…………………………………………………..........………11

Supplementary Fig. 16…………………………………………………..........………12

Supplementary Fig. 17…………………………………………………..........………12

Supplementary Fig. 18…………………………………………………..........………12

Supplementary Fig. 19…………………………………………………..........………13

Supplementary Fig. 20……………………………………………….............………13

Supplementary Fig. 21…………………………………………………..........………13

Supplementary Fig. 22…………………………………………………..........………14

Supplementary Fig. 23…………………………………………………..........………14

Supplementary Table 3…………………………………………………..........………15

Supplementary Fig. 24…………………………………………………..........………15

Supplementary Fig. 25…………………………………………………...........………16

Supplementary Fig. 26……16
Supplementary Fig. 27……16
Supplementary Fig. 28……17
Supplementary Fig. 29……17
Supplementary Fig. 30……17
Supplementary Fig. 31……18
Supplementary Fig. 32……18
Supplementary Fig. 33……19
Supplementary Fig. 34……19
Supplementary Fig. 35……20
Supplementary Fig. 36……20
Supplementary Fig. 37……20
Supplementary Fig. 38……21
Supplementary Fig. 39……21
Supplementary Fig. 40……22
Supplementary Fig. 41……22
Supplementary Fig. 42……22
Supplementary Table 4……23
Supplementary Fig. 43……23
Supplementary Fig. 44……24
Supplementary Fig. 45……24
Supplementary Fig. 46……24
Supplementary Fig. 47……25
Supplementary Fig. 48……25
Supplementary Fig. 49……25
Supplementary Fig. 50……26
Supplementary Fig. 51……26
Supplementary Fig. 52……26
Supplementary Fig. 53……27
Supplementary Table 5……27

Supplementary Fig. 54……………………………………………………..........………28
Supplementary Fig. 55……………………………………………………..........………28
Supplementary Fig. 56……………………………………………………..........………28
Supplementary Fig. 57……………………………………………………..........………29
Supplementary Fig. 58……………………………………………………..........………29
Supplementary Fig. 59……………………………………………………..........………29
Supplementary Fig. 60……………………………………………………..........………30
Supplementary Table 6…..……...…………………………………………….………............30
Supplementary Table 7…..……...…………………………………………….………............31
Supplementary Fig. 61……………………………………………………..........………31
Supplementary Fig. 62……………………………………………………..........………31
Supplementary Fig. 63……………………………………………………..........………32
Supplementary Fig. 64……………………………………………………..........………32

Figures and tables

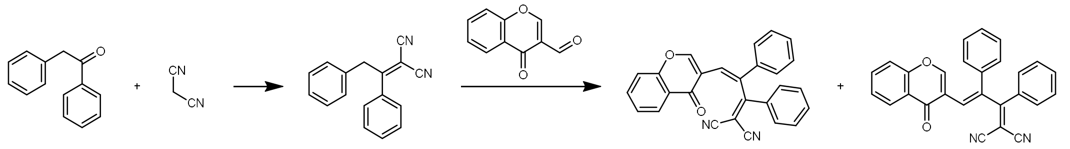

DPM *Z*-CDPM *E*-CDPM

Supplementary Fig. 1 Synthetic route for *Z*/*E*-CDPM.

Synthesis of *Z*-CDPM and *E*-CDPM. 1,2-Diphenylethan-1-one (1.96 g, 10 mmol), malononitrile (1.32 g, 20 mmol), and sodium ethoxide (510 mg, 15 mmol) were dissolved in 50 mL ethanol, and the mixture was heated to 50 °C for overnight reaction. Then it was cooled, concentrated and purified by silica gel chromatography (1:1 PE/DCM) to afford compound DPM as a white solid (Yield: 1.76 g, 72%). DPM was well characterized by ^{1}H NMR, ^{13}C NMR, and mass spectroscopy (Supplementary Fig. 2-4). ^{1}H NMR (500 MHz, $CDCl_3$), δ (ppm): 7.51-7.49 (m, 1H), 7.46-7.43 (m, 2H), 7.39-7.38 (m, 2H), 7.25-7.23 (m, 3H), 7.06-7.05 (m, 2H), 4.26 (s, 2H). ^{13}C NMR (125 MHz, $CDCl_3$), δ (ppm): 177.6, 134.9, 134.3, 132.1, 129.2, 128.9, 128.0, 127.9, 113.0, 112.7, 85.6, 43.4. HRMS (ESI): calcd. for $C_{17}H_{12}N_2$ $[M-H]^-$: 243.0928; found: 243.0923.

Compound DPM (1.22 g, 5 mmol) and 4-oxo-4H-chromene-3-carbaldehyde (1.04 g, 6 mmol) were dissolved in pyridine (4 mL) under argon, and the mixture was refluxed for 2 h. Then it was cooled, concentrated and purified by silica gel chromatography (1:2 PE/DCM) to afford *Z*-CDPM as a yellow solid (Yield: 766 mg, 36%) and *E*-CDPM as a light-yellow solid (Yield: 613 mg, 29%). *Z*-CDPM was well characterized by ^{1}H NMR, ^{13}C NMR, and mass spectroscopy (Supplementary Fig. 5-7). ^{1}H NMR (500 MHz, $C_2D_2Cl_4$), δ (ppm): 8.16-8.15 (d, J = 5.0 Hz, 1H), 7.94 (s, 1H), 7.79-7.78 (d, J = 5.0 Hz, 2H), 7.72-7.69 (t, J = 7.5 Hz, 1H), 7.46-7.39 (m, 10 H), 7.25 (s, 1H). ^{13}C NMR (125 MHz, $C_2D_2Cl_4$), δ (ppm): 175.4, 174.1, 155.9, 154.6, 140.2, 138.8, 134.6, 134.0, 133.6, 130.2, 129.5, 129.5, 129.4, 127.4, 126.9, 126.3, 126.1, 123.7, 120.9, 118.5, 113.9, 113.4, 84.7. HRMS (ESI): calcd. for $C_{27}H_{16}N_2O_2$ $[M+Na]^+$: 423.1104; found: 423.1110. *E*-CDPM was also well characterized by ^{1}H NMR, ^{13}C NMR, and mass spectroscopy (Supplementary Fig. 8-10). ^{1}H NMR (500 MHz, $C_2D_2Cl_4$), δ (ppm): 8.21-8.19 (d, J = 10.0 Hz, 1H), 7.72-7.69 (t, J = 7.5 Hz, 1H), 7.58-7.56 (d, J = 10.0 Hz, 2H), 7.54-7.52 (d, J = 10.0 Hz, 1H), 7.48-7.45 (m, 4H), 7.41-7.38 (m, 4H), 7.25-7.24 (d, J = 5 Hz, 2H), 7.16 (s, 1 H). ^{13}C NMR (125 MHz, $C_2D_2Cl_4$), δ (ppm): 176.4, 175.9, 156.8, 155.8, 139.8, 135.6, 134.6, 134.2, 132.5, 132.1, 130.0, 129.7, 129.6, 129.5, 129.2, 126.2, 126.1, 123.6, 119.8, 118.5, 114.1, 112.9, 83.7. HRMS (ESI): calcd. for $C_{27}H_{16}N_2O_2$ $[M+Na]^+$: 423.1104; found: 423.1106.

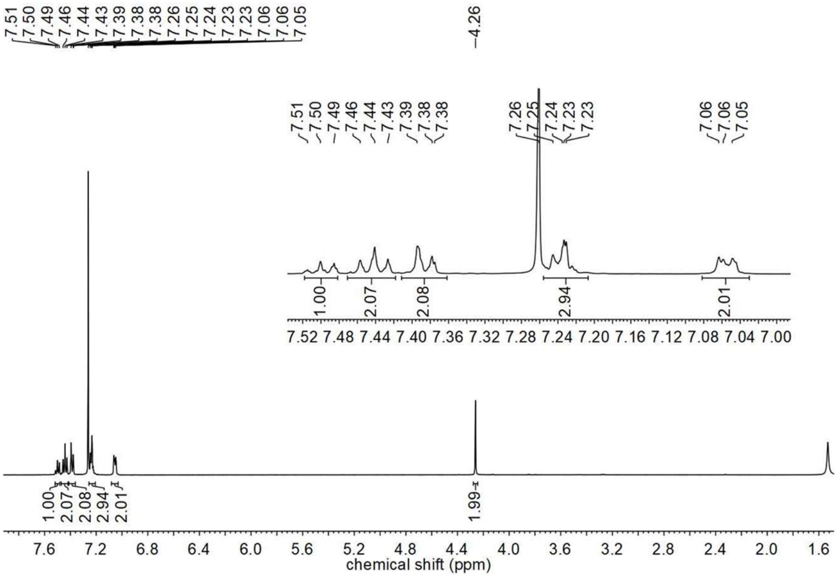

Supplementary Fig. 2 ^{1}H NMR spectrum of DPM in $CDCl_3$ (500 MHz).

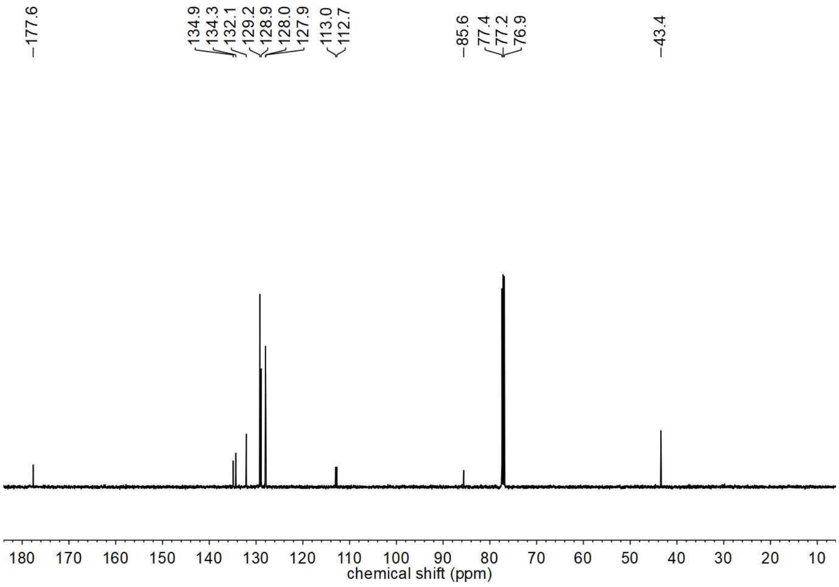

Supplementary Fig. 3 ^{13}C NMR spectrum of DPM in $CDCl_3$ (125 MHz).

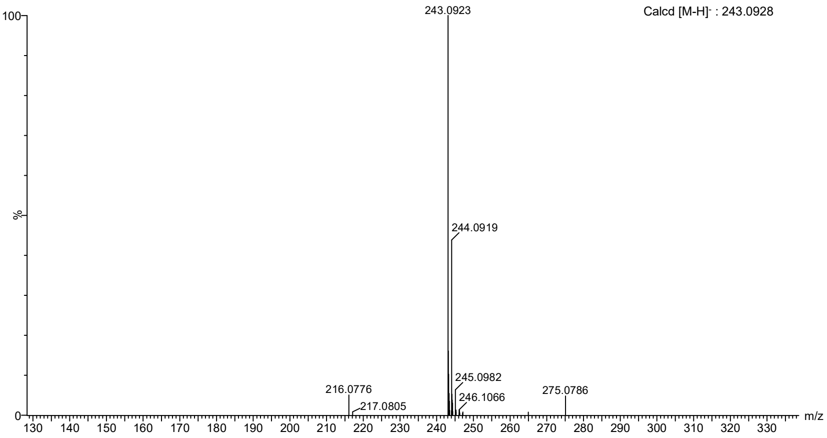

Supplementary Fig. 4 High-resolution mass spectrum of DPM.

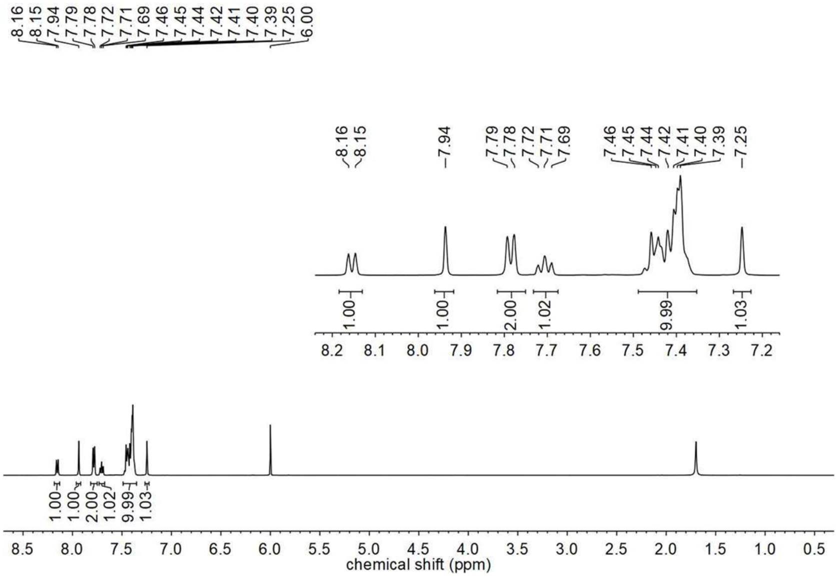

Supplementary Fig. 5 ^{1}H NMR spectrum of *Z*-CDPM in $C_2D_2Cl_4$ (500 MHz).

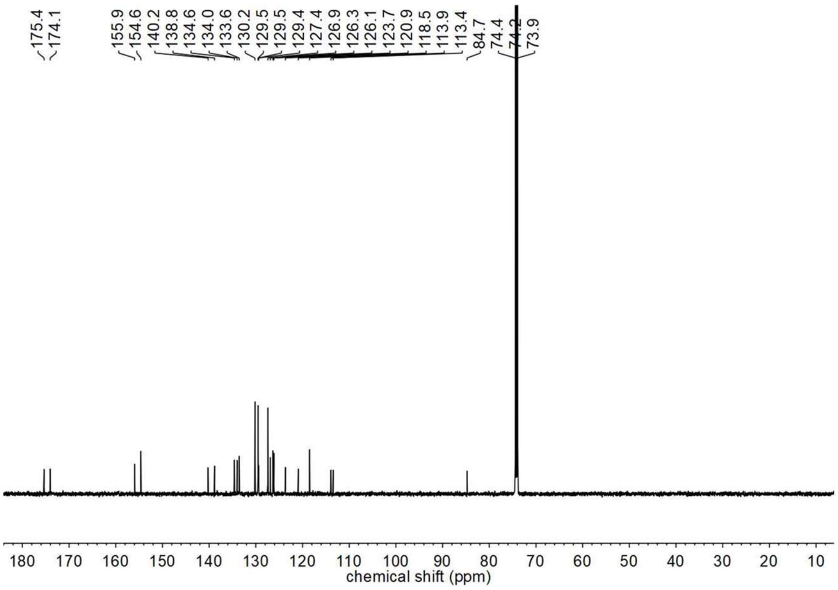

Supplementary Fig. 6 ^{13}C NMR spectrum of *Z*-CDPM in $C_2D_2Cl_4$ (125 MHz).

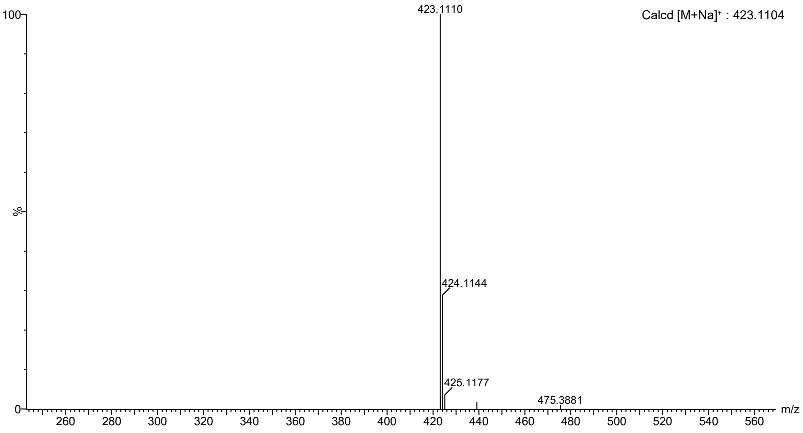

Supplementary Fig. 7 High-resolution mass spectrum of *Z*-CDPM.

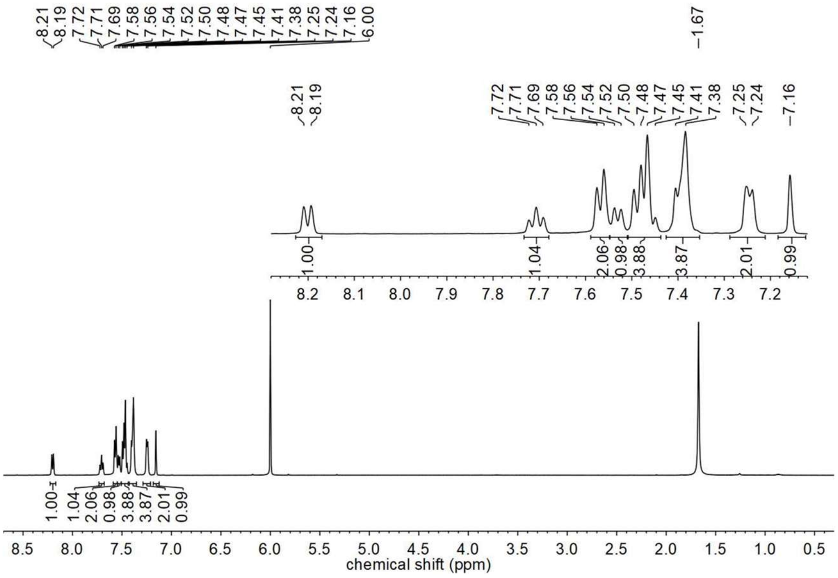

Supplementary Fig. 8 ^{1}H NMR spectrum of *E*-CDPM in $C_2D_2Cl_4$ (500 MHz).

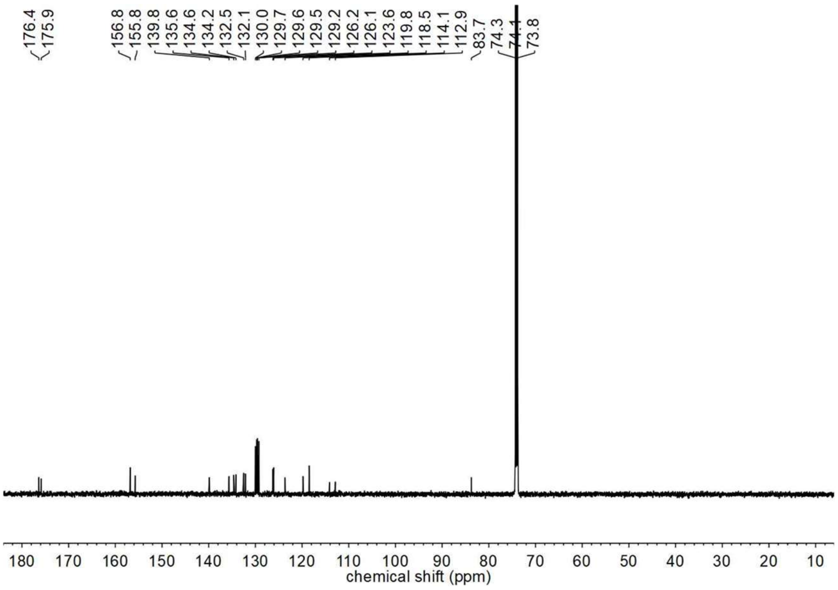

Supplementary Fig. 9 ^{13}C NMR spectrum of *E*-CDPM in $C_2D_2Cl_4$ (125 MHz).

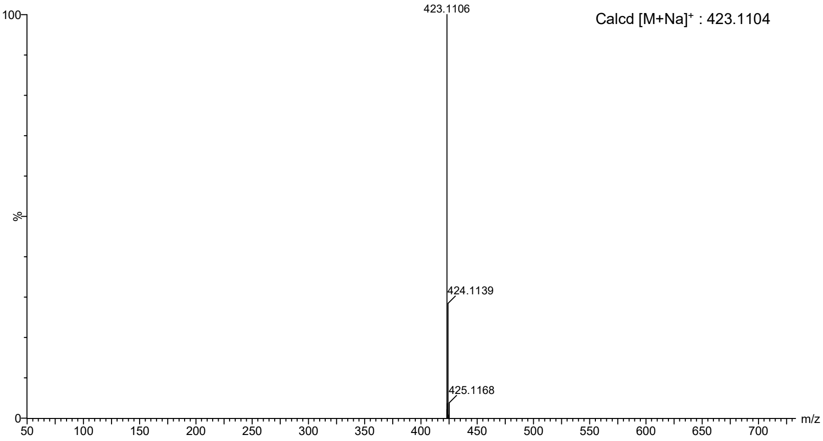

Supplementary Fig. 10 High-resolution mass spectrum of *E*-CDPM.

Supplementary Table 1. Crystal data for *Z*-CDPM and *E*-CDPM.

Identification code	*Z*-CDPM	*E*-CDPM
Empirical formula	$C_{27}H_{16}N_2O_2$	$C_{27}H_{16}N_2O_2$
Formula weight	400.42	400.42
Crystal system	Monoclinic	Triclinic
Space group	$P2_1/c$	P-1
a [Å]	8.7094(2)	9.8583(5)
b [Å]	13.5833(2)	10.3353(6)
c [Å]	17.8228(4)	23.2802(14)
a [°]	90	84.734(2)
b [°]	91.5760(10)	79.118(2)
g [°]	90	66.179(2)°
Volume [$Å^3$]	2107.68(7)	2130.6(2)
F(000)	832	834
Z	4	43
Density (calculated) [Mg/m^3]	1.262	1.251
Temperature	270(2)	300.00
Theta range for data collection [°]	2.286 to 30.529	2.447 to 30.651
Index ranges	-12<=h<=10, -19<=k<=18, -24<=l<=25	-14<=h<=14, -14<=k<=14, -33<=l<=33
Reflections collected	21362	44499
Independent reflections	6365 [R(int) = 0.0494]	12897 [R(int) = 0.0569]
Data / restraints / parameters	6365 / 0 / 280	12897 / 0 / 559
Goodness-of-fit on F^2	1.428	1.277
CCDC	2357788	2357790

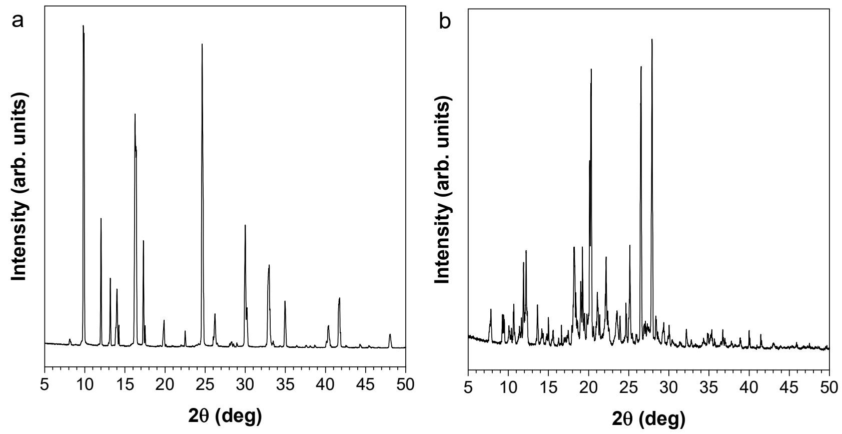

Supplementary Fig. 11 X-ray diffraction diagrams of the crystal powders of *Z*-CDPM (a) and *E*-CDPM (b).

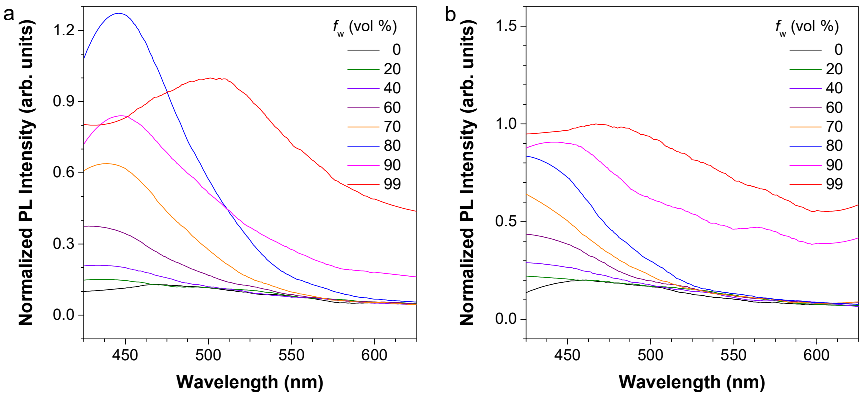

Supplementary Fig. 12 Photoluminescence (PL) spectra of *Z*-CDPM (a, 10 μM) and *E*-CDPM (a, 10 μM) in THF/water mixtures with different water fractions (f_w). λ_{ex} = 365 nm.

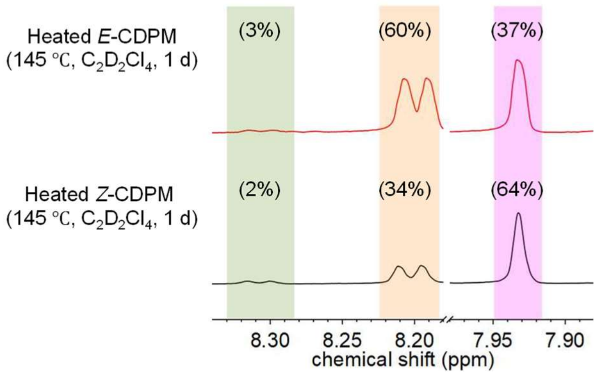

Supplementary Fig. 13 Indicative sections of ^{1}H NMR spectra of *Z*-CDPM and *E*-CDPM after heating at 145 °C in $C_2D_2Cl_4$ for 1 d (500 MHz). *Z*-CDPM was highlighted in purple, *E*-CDPM was highlighted in orange, and DPXDC was highlighted in green.

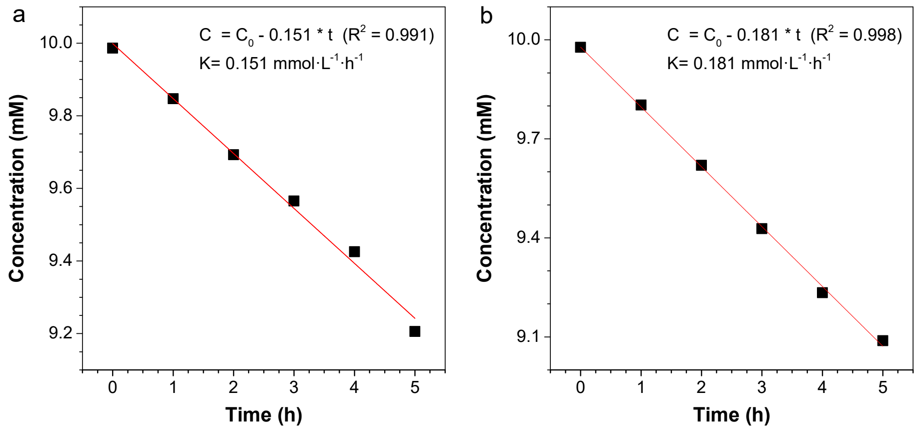

Supplementary Fig. 14 Linear fitting curve of the concentration of *Z*-CDPM (a) and *E*-CDPM (b) towards heating time. The $C_2D_2Cl_4$ solution of *Z*-CDPM (10 mM) and *E*-CDPM (mM) was heat at 145 °C in $C_2D_2Cl_4$.for different time (0, 1, 2, 3, 4, 5 h) and then the concentration was determined by ^{1}H NMR data.

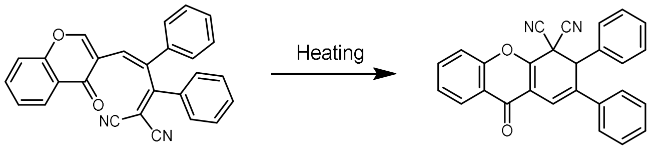

Supplementary Fig. 15 Synthetic route for DPXDC.

Synthesis of DPXDC. Compound *Z*-CDPM (240 mg, 0.6 mmol) was dissolved in diphenyl ether (10 mL), and the mixture was heated at 200 °C for 2 h. Then it was cooled and purified by silica gel chromatography (1:1 PE/DCM) to afford DPXDC as a white solid (Yield: 208 mg, 87%). DPXDC was well characterized by ^{1}H NMR, ^{13}C NMR, and mass spectroscopy (Supplementary Fig. 16-18). ^{1}H NMR (500 MHz, $C_2D_2Cl_4$), δ (ppm): 8.31-8.30 (d, J = 5 Hz, 1H), 7.80-7.77 (t, J = 7.5 Hz, 1H), 7.58-7.53 (m, 3H), 7.46-7.45 (m, 2H), 7.38-7.36 (m, 8H), 4.80 (s, 1H). ^{13}C NMR (125 MHz, $C_2D_2Cl_4$), δ (ppm): 173.9, 156.0, 146.9, 136.8, 135.5, 135.3, 130.5, 130.2, 129.9, 129.6, 129.5, 129.4, 127.1, 126.7, 126.3, 123.6, 119.0, 118.3, 115.8, 112.8, 110.2, 52.7, 42.8. HRMS (ESI): calcd. for $C_{27}H_{16}N_2O_2$ $[M+Na]^+$: 423.1104; found: 423.1109.

Supplementary Table 2. Crystal data for DPXDC.

Identification code	DPXDC
Empirical formula	$C_{27}H_{16}N_2O_2$
Formula weight	400.42
Crystal system	Monoclinic
Space group	$P2_1/c$
a [Å]	15.0244(5)
b [Å]	15.1694(5)
c [Å]	8.8412(3)
a [°]	90
b [°]	94.083(3)
g [°]	90
Volume [Å^3]	2009.89(12)
F(000)	832
Z	4
Density (calculated) [Mg/m^3]	1.323
Temperature	170(2)
Theta range for data collection [°]	1.910 to 31.001
Index ranges	-20<=h<=16, -14<=k<=21, -11<=l<=12
Reflections collected	16175
Independent reflections	5483 [R(int) = 0.0306]
Data / restraints / parameters	5483 / 0 / 280
Goodness-of-fit on F^2	1.042
CCDC	2357744

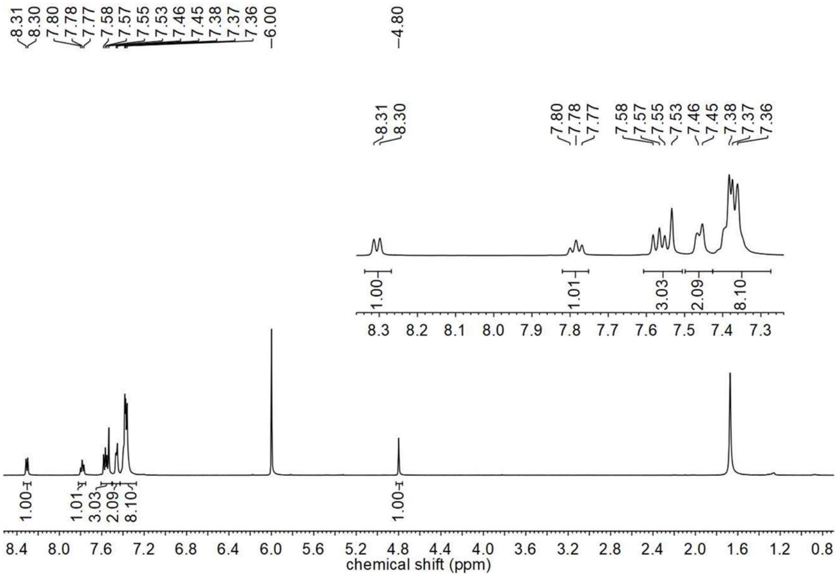

Supplementary Fig. 16 ^{1}H NMR spectrum of DPXDC in $C_2D_2Cl_4$ (500 MHz).

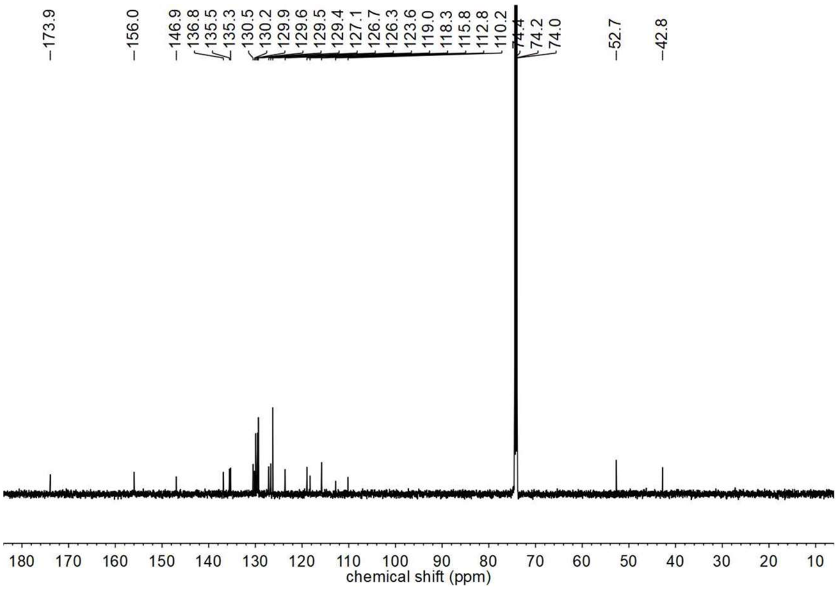

Supplementary Fig. 17 ^{13}C NMR spectrum of DPXDC in $C_2D_2Cl_4$ (125 MHz).

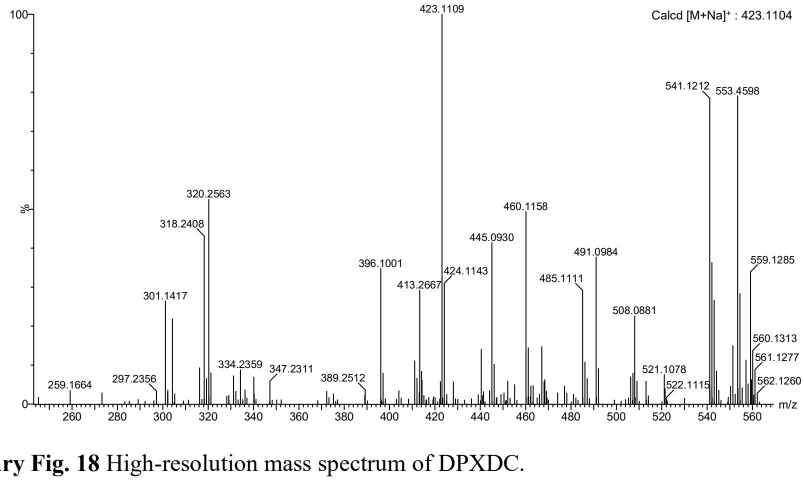

Supplementary Fig. 18 High-resolution mass spectrum of DPXDC.

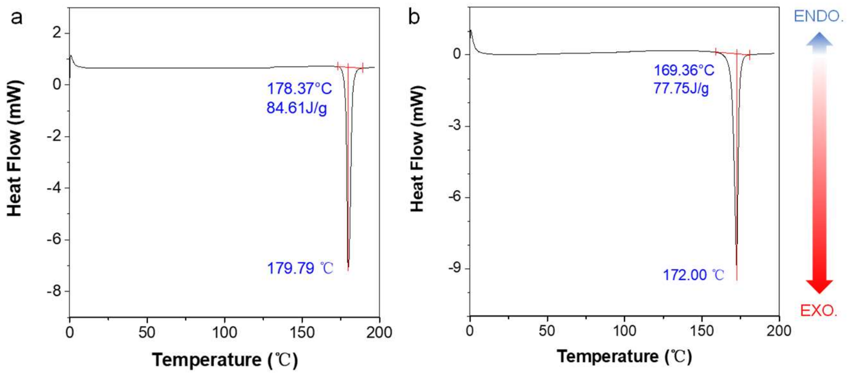

Supplementary Fig. 19 The differential scanning calorimeter curves of *Z*-CDPM (a) and *E*-CDPM (b). EXO. exothermic, ENDO. endothermic.

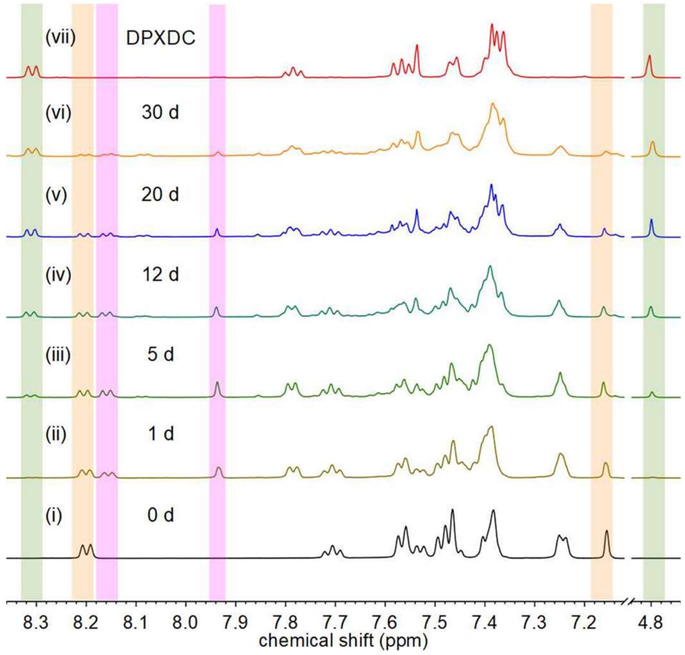

Supplementary Fig. 20 ^{1}H NMR spectra of as prepared *E*-CDPM before and after heating at 145 °C in $C_2D_2Cl_4$ (500 MHz) for different time (1, 5, 12, 20, 30 d). *Z*-CDPM was highlighted in purple, *E*-CDPM was highlighted in orange, and DPXDC was highlighted in green.

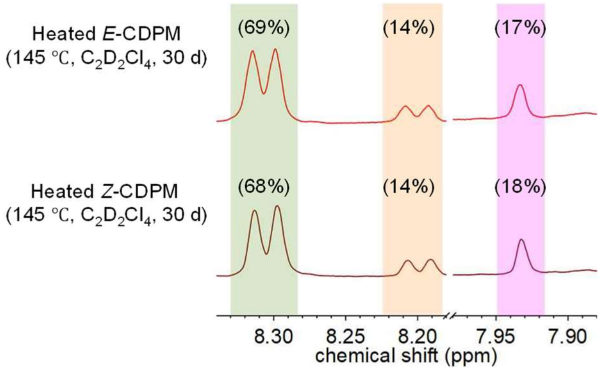

Supplementary Fig. 21 Indicative sections of ^{1}H NMR spectra of *Z*-CDPM and *E*-CDPM after heating at 145 °C in $C_2D_2Cl_4$ for 30 d (500 MHz). *Z*-CDPM was highlighted in purple, *E*-CDPM was highlighted in orange, and DPXDC was highlighted in green.

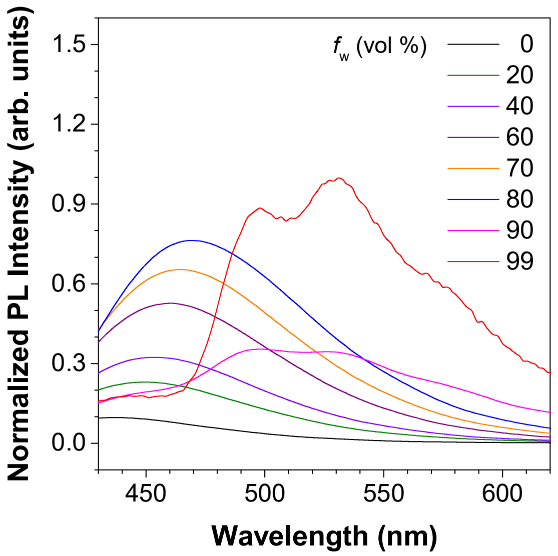

Supplementary Fig. 22 Photoluminescence (PL) spectra of DPXDC (10 μM) in THF/water mixtures. λ_{ex} = 365 nm.

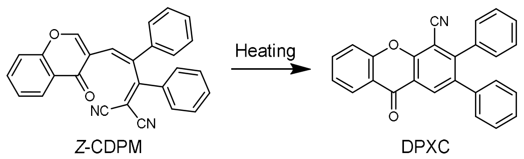

Supplementary Fig. 23 Synthetic route of DPXC.

Synthesis of DPXC. Compound *Z*-CDPM (200 mg, 0.5 mmol) was dissolved in DCM (10 mL), followed by addition of silica gel (15 g). The mixture was heated at 145 °C for 6 h, and then cooled and purified by silica gel chromatography (1:1 PE/DCM) to afford DPXC as a white solid (Yield: 163 mg, 82%). DPXC was well characterized by ^{1}H NMR, ^{13}C NMR, and mass spectroscopy (Supplementary Fig. 24-26). ^{1}H NMR (500 MHz, $C_2D_2Cl_4$), δ (ppm): 8.53 (s, 1H), 8.35-8.34 (d, J = 5 Hz, 1H), 7.87-7.84 (t, J = 7.5 Hz, 1H), 7.71-7.70 (d, J = 5 Hz, 1H), 7.53-7.50 (t, J = 7.5 Hz, 1H), 7.41-7.36 (m, 3H), 7.27-7.24 (m, 5H), 7.09-7.08 (m, 2H). ^{13}C NMR (125 MHz, $C_2D_2Cl_4$), δ (ppm): 175.8, 156.4, 155.9, 151.6, 138.3, 138.3, 136.2, 136.2, 132.7, 130.1, 130.0, 129.3, 128.7, 128.5, 127.9, 127.0, 125.6, 121.8, 121.3, 118.7, 114.5, 104.0. HRMS (ESI): calcd. for $C_{26}H_{15}NO_2$ [M+Na]$^{+}$: 396.0995; found: 396.0999.

Supplementary Table 3. Crystal data for DPXC.

Identification code	DPXC
Empirical formula	$C_{26}H_{15}NO_2$
Formula weight	396.10
Crystal system	Monoclinic
Space group	$P2_1/c$
a [Å]	6.8317(2)
b [Å]	21.7155(7)
c [Å]	26.5027(8)
a [°]	90
b [°]	91.5180(10)
g [°]	90
Volume [$Å^3$]	3930.4(2)
F(000)	1738
Z	79
Density (calculated) [Mg/m^3]	1.436
Temperature	300(2)
Theta range for data collection [°]	2.425 to 29.697
Index ranges	-9<=h<=9, -30<=k<=25, -28<=l<=36
Reflections collected	32554
Independent reflections	10865 [R(int) = 0.1463]
Data / restraints / parameters	10865 / 0 / 487
Goodness-of-fit on F^2	2.393
CCDC	2357785

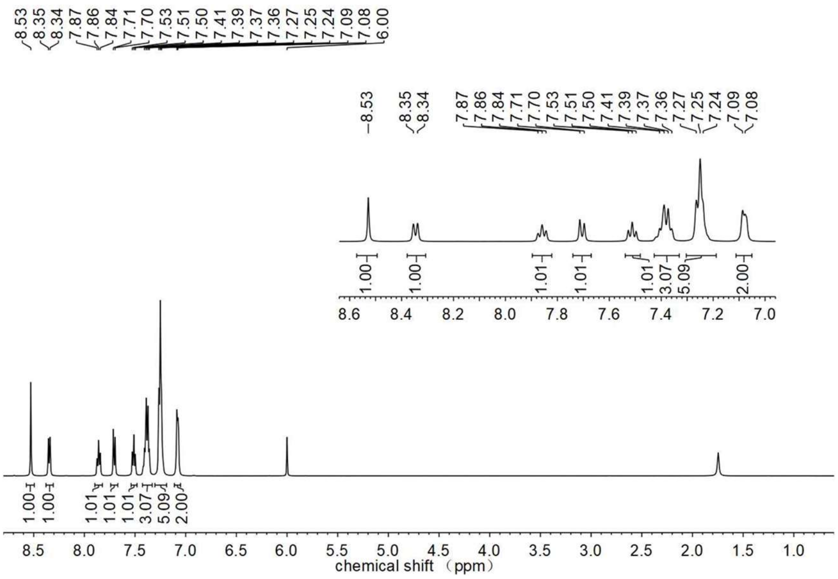

Supplementary Fig. 24 ^1H NMR spectrum of DPXC in $C_2D_2Cl_4$ (500 MHz).

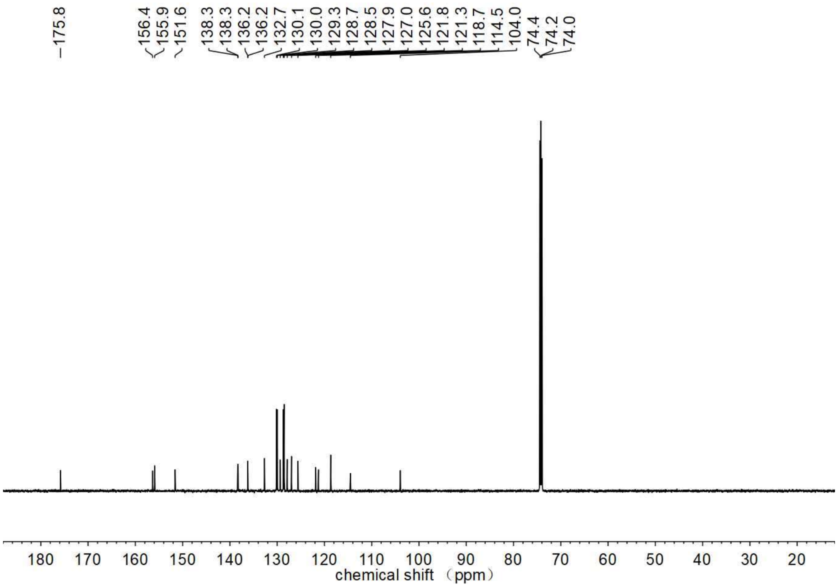

Supplementary Fig. 25 ^{13}C NMR spectrum of DPXC in $C_2D_2Cl_4$ (125 MHz).

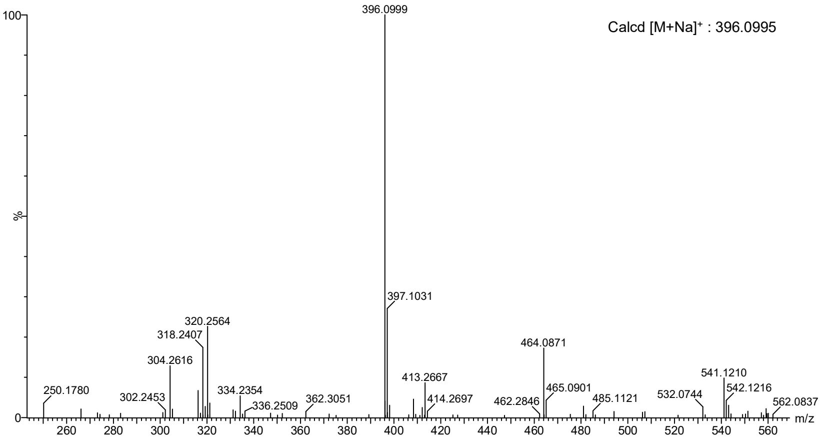

Supplementary Fig. 26 High-resolution mass spectrum of DPXC.

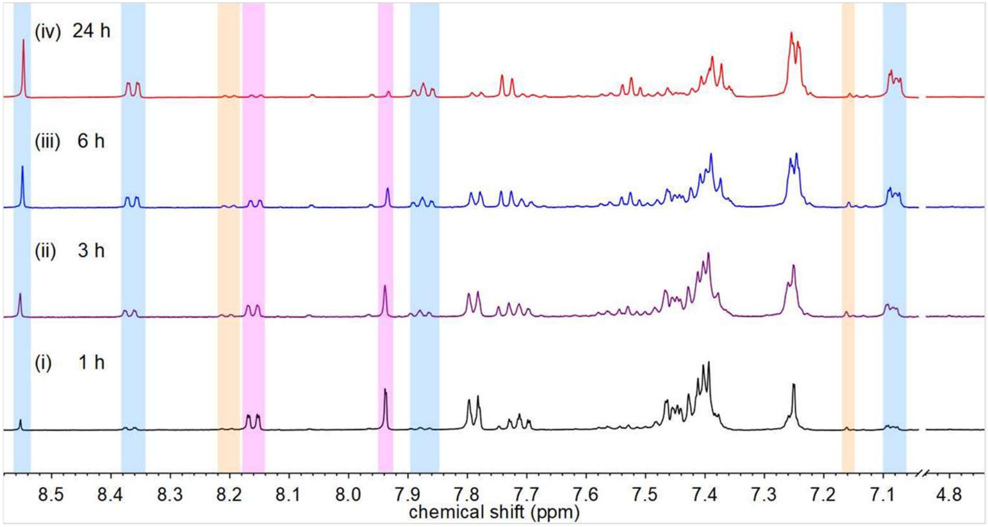

Supplementary Fig. 27 ^1H NMR spectra of *Z*-CDPM ($C_2D_2Cl_4$, 500 MHz) after heating at 145 °C in silica gel for different time (1, 3, 6, 24 h). *Z*-CDPM was highlighted in purple, *E*-CDPM was highlighted in orange, DPXDC is highlighted in green, and DPXC was highlighted in blue.

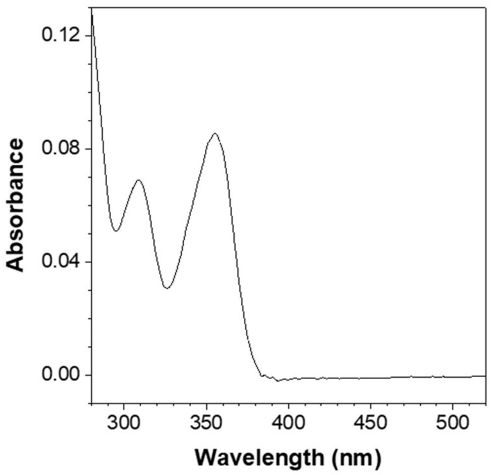

Supplementary Fig. 28 Absorption spectra of DPXC (10 μM) in THF.

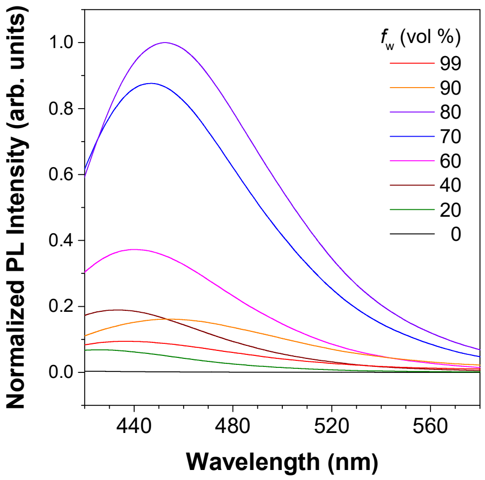

Supplementary Fig. 29 Photoluminescence (PL) spectra of DPXC in THF/water mixtures (10 μM). λ_{ex} = 355 nm.

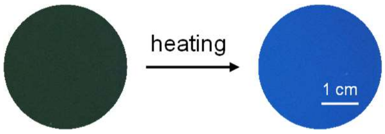

Supplementary Fig. 30 Fluorescent images of Z-CDPM before and after heating at 200 °C for 10 s in thin-layer chromatography plate. Scale bar: 1 cm.

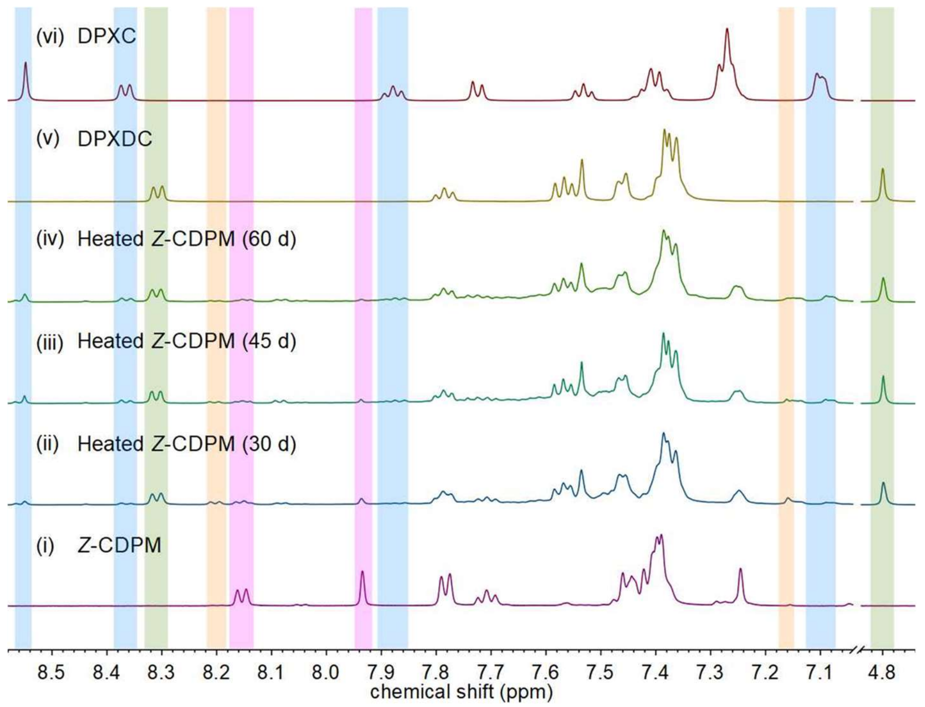

Supplementary Fig. 31 ^{1}H NMR spectra of *Z*-CDPM (500 MHz) after heating at 145 °C in $C_2D_2Cl_4$ for different time (30, 45, 60 d). *Z*-CDPM was highlighted in purple, *E*-CDPM was highlighted in orange, DPXDC was highlighted in green, and DPXC was highlighted in blue.

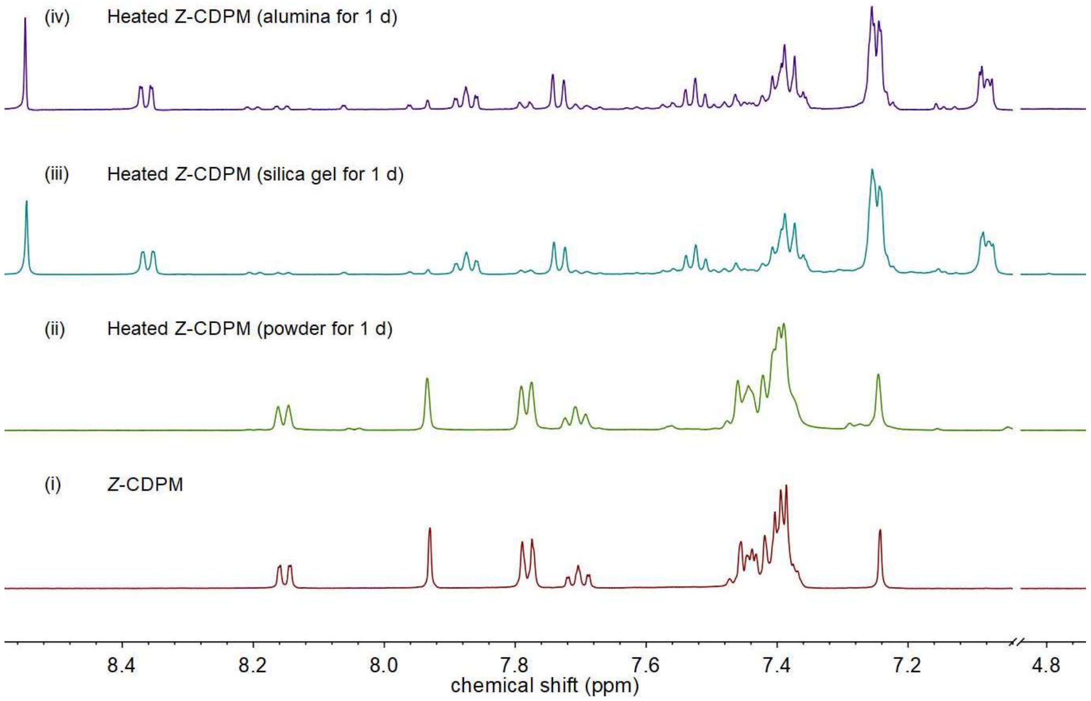

Supplementary Fig. 32 ^{1}H NMR spectra of *Z*-CDPM powder before and after heating at 145 °C in different media for 1 d ($C_2D_2Cl_4$, 500 MHz).

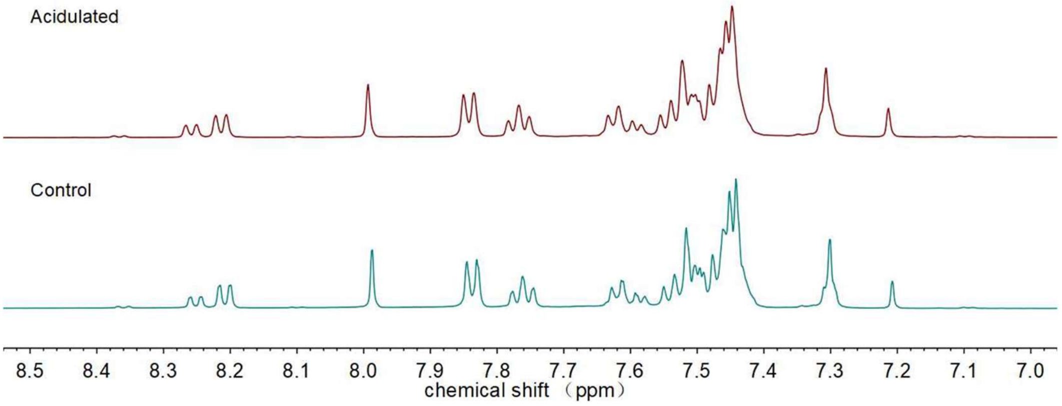

Supplementary Fig. 33. ^{1}H NMR spectra of *Z*-CDPM before and after acetic acid (0.5%, v/v) acidification in $C_2D_2Cl_4$ (500 MHz). The sample was heated at 145 °C for 1d.

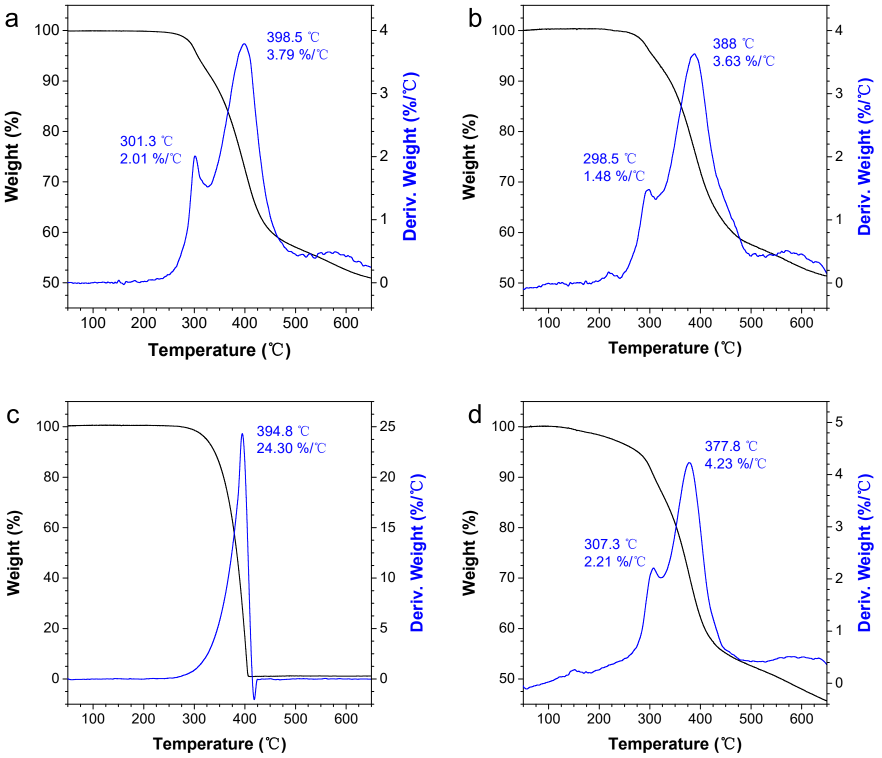

Supplementary Fig. 34 Thermogravimetric analysis diagrams of *Z*-CDPM (a), DPXDC (b), DPXC (c) and *E*-CDPM (d) recorded under nitrogen at a heating rate of 10 °C/min.

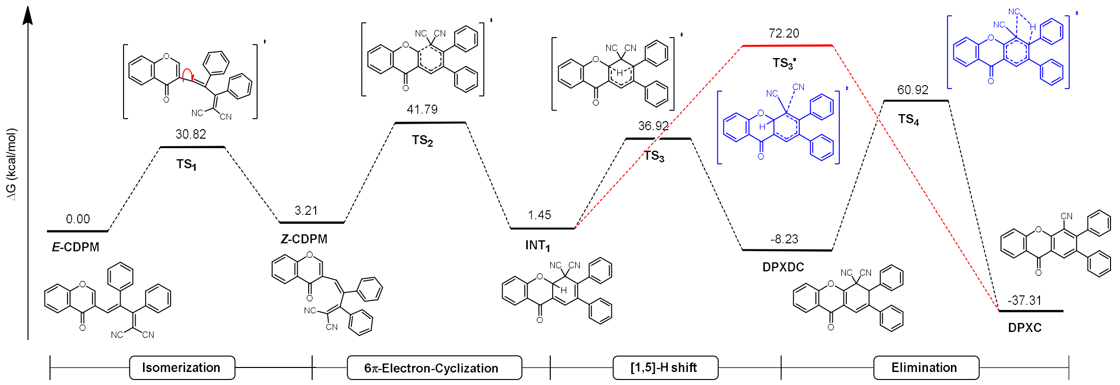

Supplementary Fig. 35 Energy profile for the thermal reactions of *Z*-CDPM calculated at the M06-2X/6-31G(d,p) level without solvent correction. TS transition state, INT intermediate.

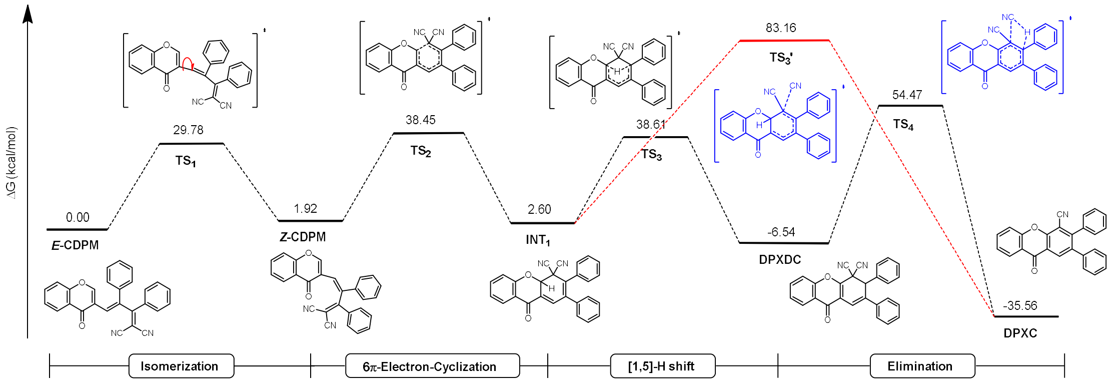

Supplementary Fig. 36 Energy profile for the thermal reactions of *Z*-CDPM calculated at the M06-2X/6-31G(d,p) level with solvent correction. TS transition state, INT intermediate.

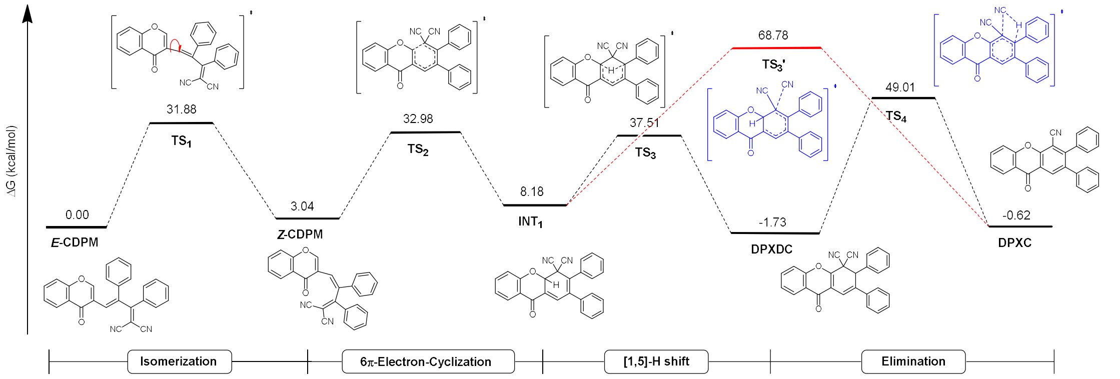

Supplementary Fig. 37 Energy profile for the thermal reactions of *Z*-CDPM calculated at the TPSS/6-31G(d,p) level without solvent correction. TS transition state, INT intermediate.

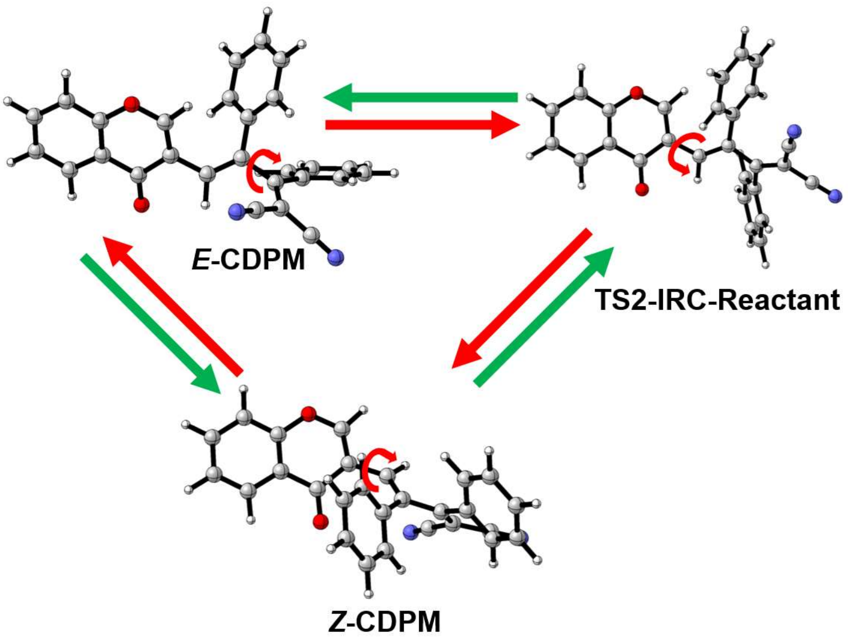

Supplementary Fig. 38 Comparison of the optimized structures of *E*-CDPM and *Z*-CDPM with the reactant-side structure obtained from the IRC pathway of TS2. The TS2-IRC-Reactant can interconvert with both *E*-CDPM and *Z*-CDPM via single-bond rotations, indicating that these species share a flexible conformational landscape. TS transition state, IRC intrinsic reaction coordinate.

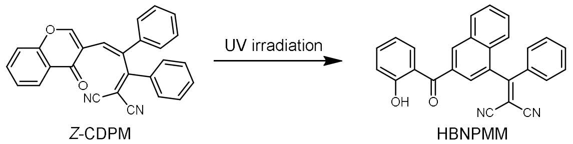

Supplementary Fig. 39 Synthetic route for HBNPMM.

Synthesis of HBNPMM. Compound *Z*-CDPM (100 mg, 0.25 mmol) was dissolved in chloroform (10 mL) and stirred under a high-pressure mercury lamp (100 W) for 2 h. Then it was cooled, concentrated and purified by silica gel chromatography (1:2 PE/DCM) to afford HBNPMM as a white solid (Yield: 87 mg, 87%). HBNPMM was well characterized by ^{1}H NMR, ^{13}C NMR, and mass spectroscopy (Supplementary Fig. 40-42). ^{1}H NMR (500 MHz, $C_2D_2Cl_4$), δ (ppm): 11.84 (s, 1H), 8.44 (s, 1H), 8.09-8.08 (d, J = 5 Hz, 1H), 7.85 (s, 1H), 7.74-7.73 (d, J = 5 Hz, 1H), 7.68-7.65 (t, J = 7.5 Hz, 1H), 7.63-7.59 (m, 5H), 7.53-7.47 (m, 3H), 7.15-7.14 (d, J = 5 Hz, 1H)), 7.01-6.98 (t, J = 7.5 Hz, 1H). ^{13}C NMR (125 MHz, $C_2D_2Cl_4$), δ (ppm): 200.2, 173.8, 163.3, 137.4, 135.2, 134.4, 134.3, 134.3, 134.0, 133.9, 133.1, 131.9, 130.6, 130.5, 129.9, 129.7, 128.4, 127.7, 125.1, 119.8, 119.2, 118.9, 113.8, 113.6, 85.4. HRMS (ESI): calcd. for $C_{27}H_{16}N_2O_2$ $[M+Na]^+$: 423.1104; found: 423.1112.

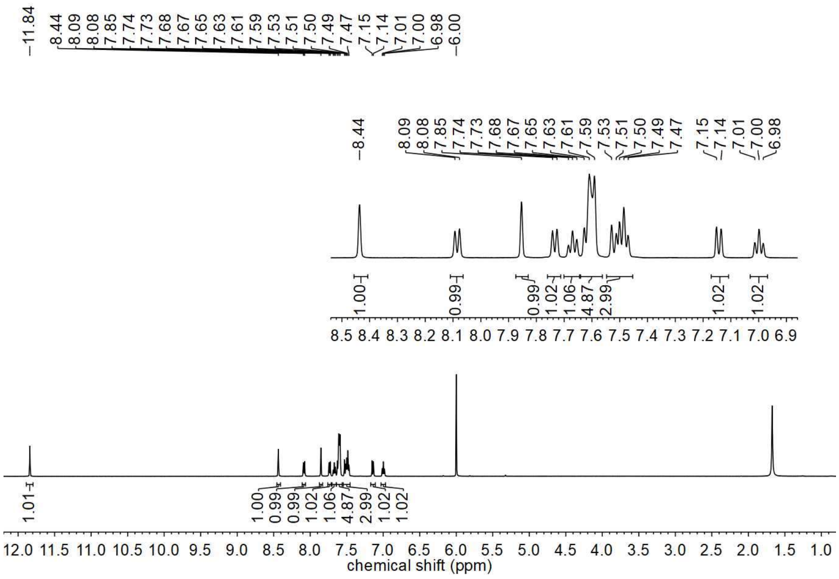

Supplementary Fig. 40 ^{1}H NMR spectrum of HBNPMM in $C_2D_2Cl_4$ (500 MHz).

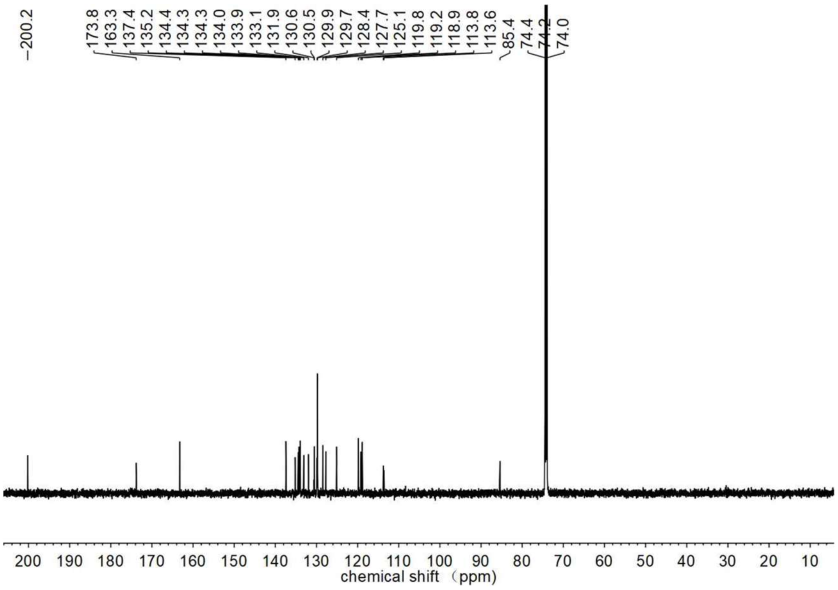

Supplementary Fig. 41 ^{13}C NMR spectrum of HBNPMM in $C_2D_2Cl_4$ (125 MHz).

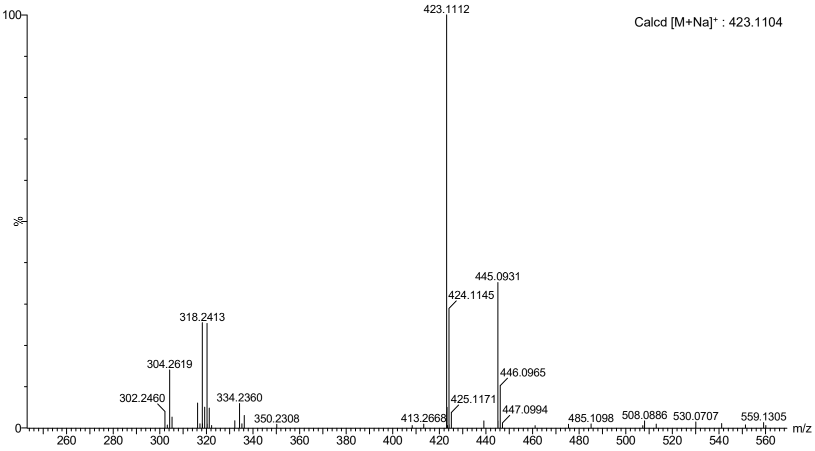

Supplementary Fig. 42 High-resolution mass spectrum of HBNPMM.

Supplementary Table 4. Crystal data for HBNPMM.

Identification code	HBNPMM
Empirical formula	$C_{27}H_{16}N_2O_2$
Formula weight	400.42
Crystal system	Monoclinic
Space group	$P2_1/c$
a [Å]	13.3799(3)
b [Å]	11.9865(2)
c [Å]	14.1652(3)
a [°]	90
b [°]	116.8740(10)°
g [°]	90
Volume [$Å^3$]	2026.44(7)
F(000)	902
Z	41
Density (calculated) [Mg/m^3]	1.446
Temperature	270(2)
Theta range for data collection [°]	2.342 to 30.518
Index ranges	-19<=h<=17, -16<=k<=17, -20<=l<=20
Reflections collected	23022
Independent reflections	6121 [R(int) = 0.0481]
Data / restraints / parameters	6121 / 0 / 281
Goodness-of-fit on F^2	1.165
CCDC	2357787

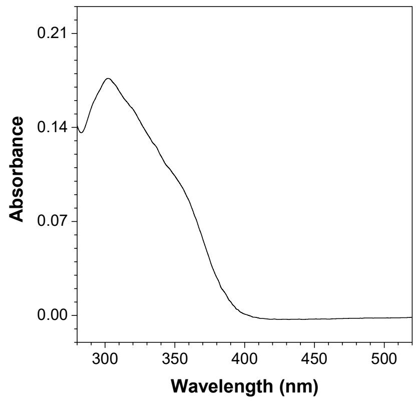

Supplementary Fig. 43 Absorption spectrum of HBNPMM (10 μM) in THF.

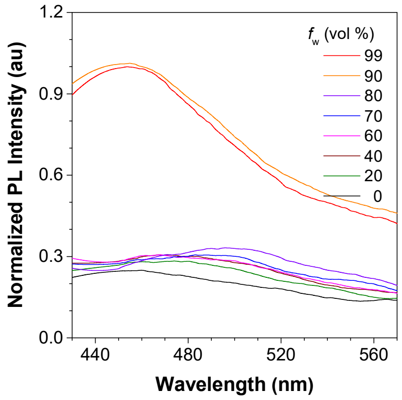

Supplementary Fig. 44 Photoluminescence (PL) spectra of HBNPMM (10 μM) in THF/water mixtures. λ_{ex} = 365 nm.

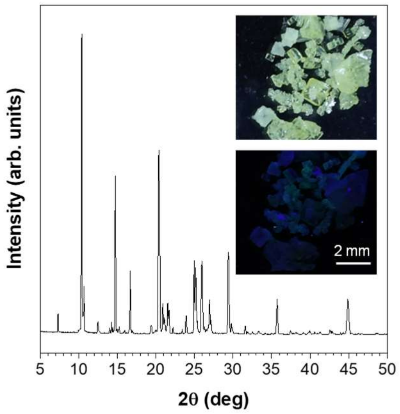

Supplementary Fig. 45 X-ray diffraction diffractograms of the crystal powder of HBNPMM. Insets: photographs of crystals of HBNPMM taken under day light (upper) and UV illumination (down). Scale bar: 2 mm.

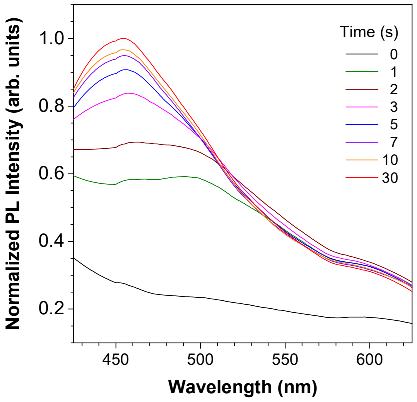

Supplementary Fig. 46 Changes in photoluminescence (PL) spectrum of *E*-CDPM before and after UV irradiation at 365 nm for different time (1, 2, 3, 5, 7,10 and 30 s). λ_{ex} = 365 nm.

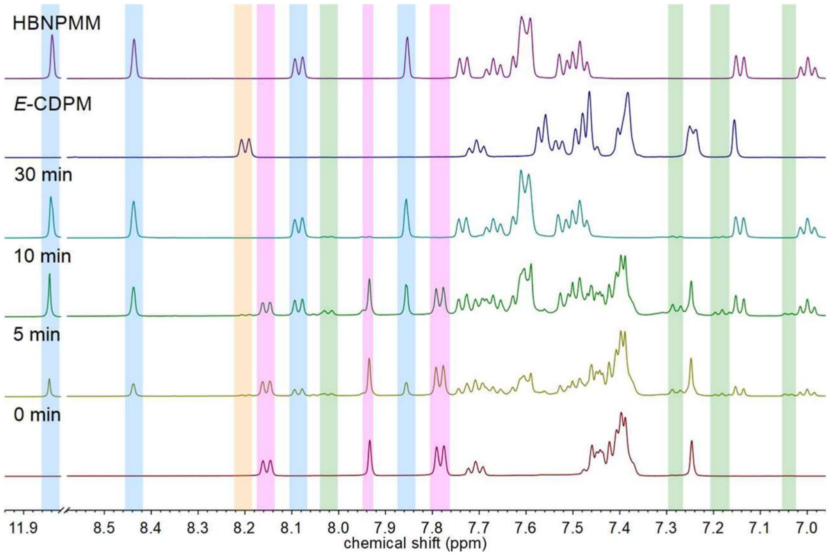

Supplementary Fig. 47 ^{1}H NMR spectra of *Z*-CDPM after UV irradiation in $C_2D_2Cl_4$ (500 MHz) for different time (0, 5, 10, 30 min). *Z*-CDPM was highlighted in purple, *E*-CDPM was highlighted in orange, HBNPMM was highlighted in blue, and a possible intermediate(s) was highlighted in green.

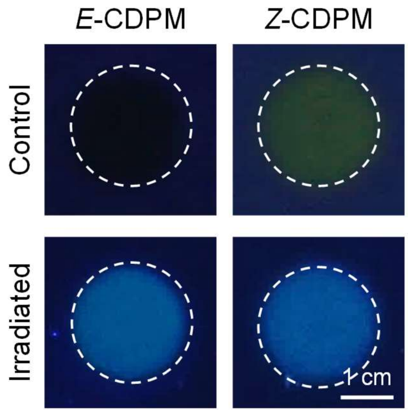

Supplementary Fig. 48 Photographs of *Z*/*E*-CDPM before and after UV irradiation at 365 nm for 10 s. Scale bar: 1 cm.

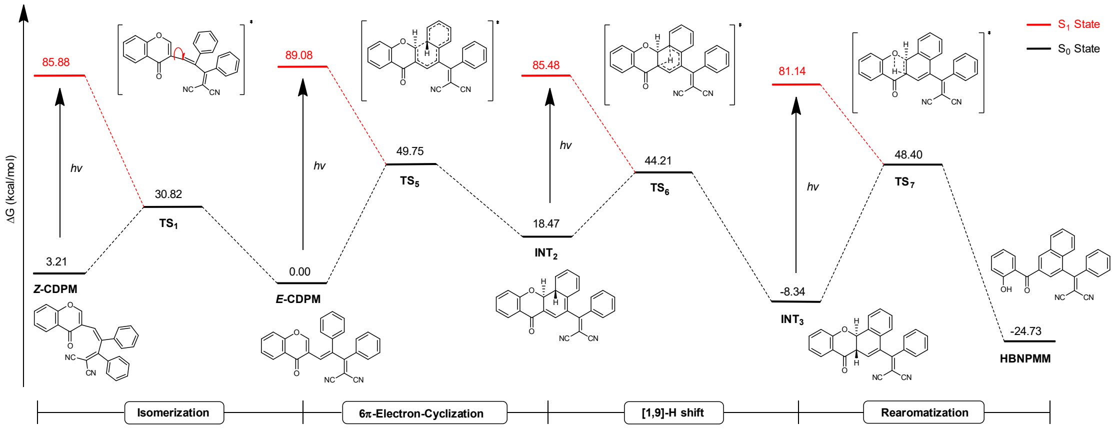

Supplementary Fig. 49 Energy profile for the photoarrangement of *Z*-CDPM calculated at the M06-2X/6-31G(d,p) level without solvent correction. TS transition state, INT intermediate.

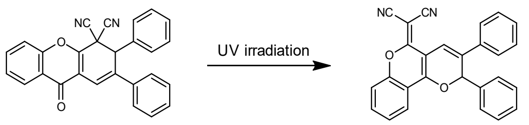

DPXDC DPPCYM

Supplementary Fig. 50 Synthetic route for DPPCYM.

Synthesis of DPPCYM. Compound DPXDC (100 mg, 0.25 mmol) was dissolved in chloroform (10 mL) and stirred under a high-pressure mercury lamp (100 W) for 2 h. Then it was cooled, concentrated and purified by silica gel chromatography (1:2 PE/DCM) to afford DPPCYM as a white solid (Yield: 88 mg, 88%). DPPCYM was well characterized by ^{1}H NMR, ^{13}C NMR, and mass spectroscopy (Supplementary Fig. 51-53). ^{1}H NMR (500 MHz, $C_2D_2Cl_4$), δ (ppm): 7.90-7.88 (d, J = 10 Hz, 1H), 7.79 (s, 1H), 7.65-7.62 (t, J = 7.5 Hz, 1H), 7.55-7.54 (m, 2H), 7.49-7.48 (m, 2H), 7.45-7.37 (m, 8H), 6.74 (s, 1H). ^{13}C NMR (125 MHz, $C_2D_2Cl_4$), δ (ppm): 169.6, 156.8, 153.4, 135.9, 135.5, 134.5, 131.1, 130.4, 129.6, 129.6, 128.4, 126.4, 125.9, 123.4, 117.1, 116.6, 115.6, 114.8, 113.6, 104.9, 80.1, 54.0. HRMS (ESI): calcd. for $C_{27}H_{16}N_2O_2$ $[M+Na]^+$: 423.1104; found: 423.1111.

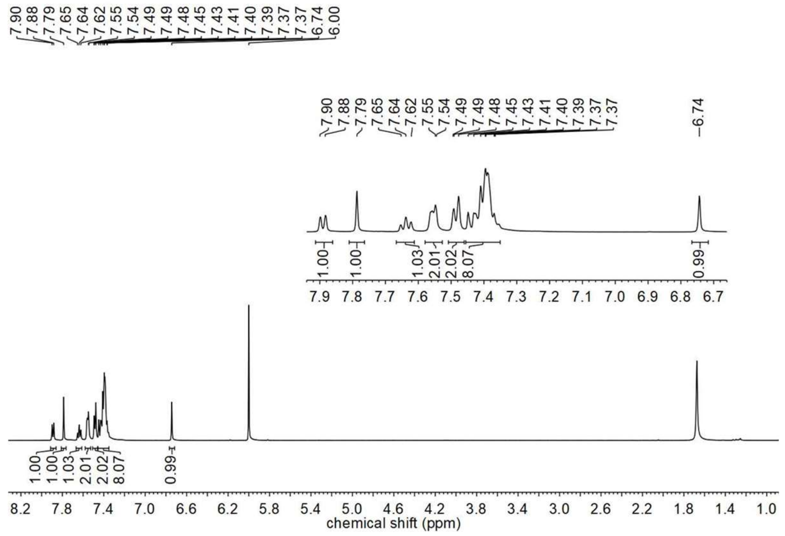

Supplementary Fig. 51 ^{1}H NMR spectrum of DPPCYM in $C_2D_2Cl_4$ (500 MHz).

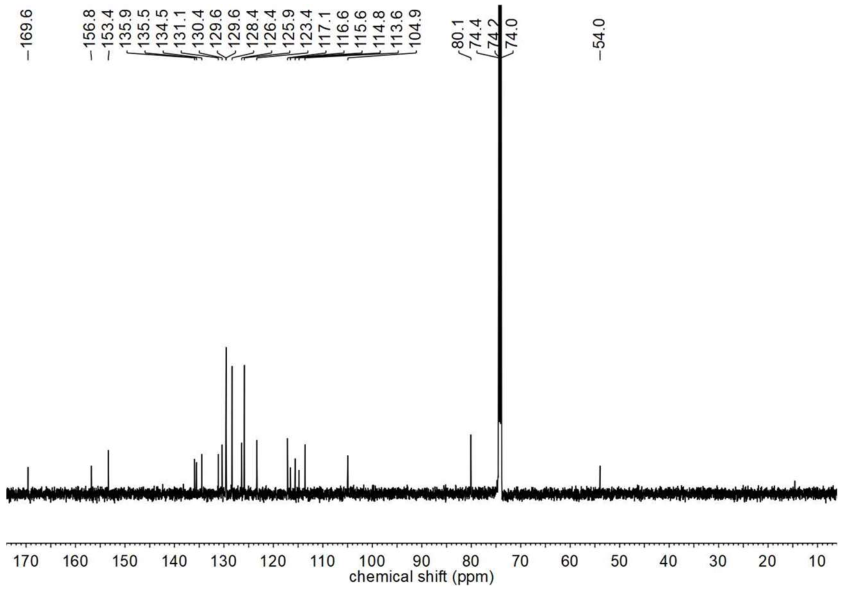

Supplementary Fig. 52 ^{13}C NMR spectrum of DPPCYM in $C_2D_2Cl_4$ (125 MHz).

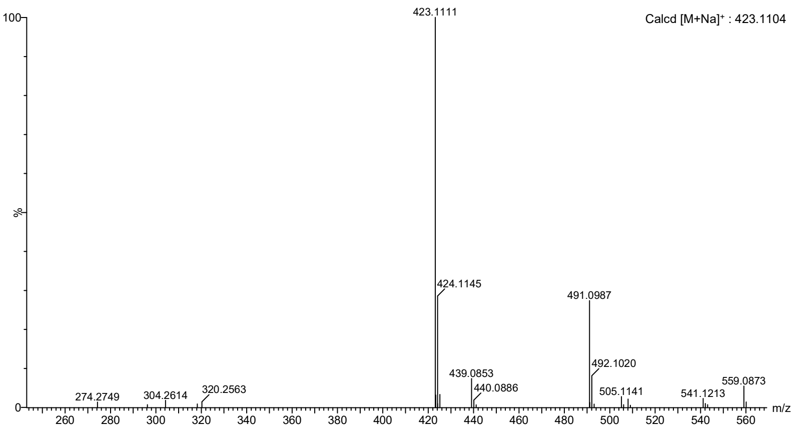

Supplementary Fig. 53 High-resolution mass spectrum of DPPCYM.

Supplementary Table 5. Crystal data for DPPCYM.

Identification code	DPPCYM
Empirical formula	$C_{27}H_{16}N_2O_2$
Formula weight	400.42
Crystal system	Triclinic
Space group	P-1
a [Å]	10.5042(7)
b [Å]	10.6272(7)
c [Å]	20.8526(15)
a [°]	90.039(3)
b [°]	95.897(2)
g [°]	116.979(2)
Volume [$Å^3$]	2060.5(2)
F(000)	832
Z	4
Density (calculated) [Mg/m^3]	1.291
Temperature	293(2)
Theta range for data collection [°]	2.190 to 29.109
Index ranges	-14<=h<=14, -14<=k<=14, -28<=l<=28
Reflections collected	32335
Independent reflections	10831 [R(int) = 0.0569]
Data / restraints / parameters	10831 / 0 / 559
Goodness-of-fit on F^2	1.119
CCDC	2357792

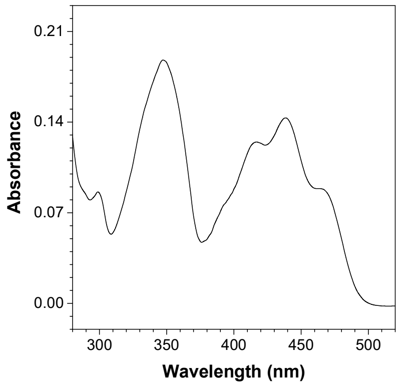

Supplementary Fig. 54 Absorption spectrum of DPPCYM (10 μM).

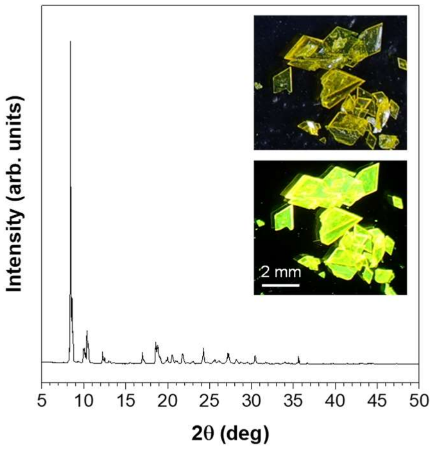

Supplementary Fig. 55 X-ray diffraction diffractogram of the crystal powder of DPPCYM. Insets: photographs of crystals of DPPCYM taken under day light (upper) and UV illumination (down). Scale bar: 2 mm.

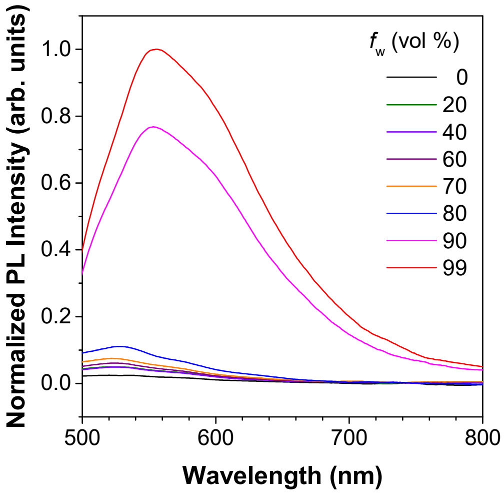

Supplementary Fig. 56 Photoluminescence (PL) spectra of DPPCYM (a, 10 μM) in THF/water mixtures with different water fractions (f_w). λ_{ex} = 440 nm.

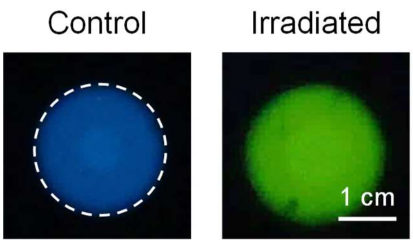

Supplementary Fig. 57 Photographs of DPXDC before and after UV irradiation at 365 nm for 10 s. Scale bar: 1 cm.

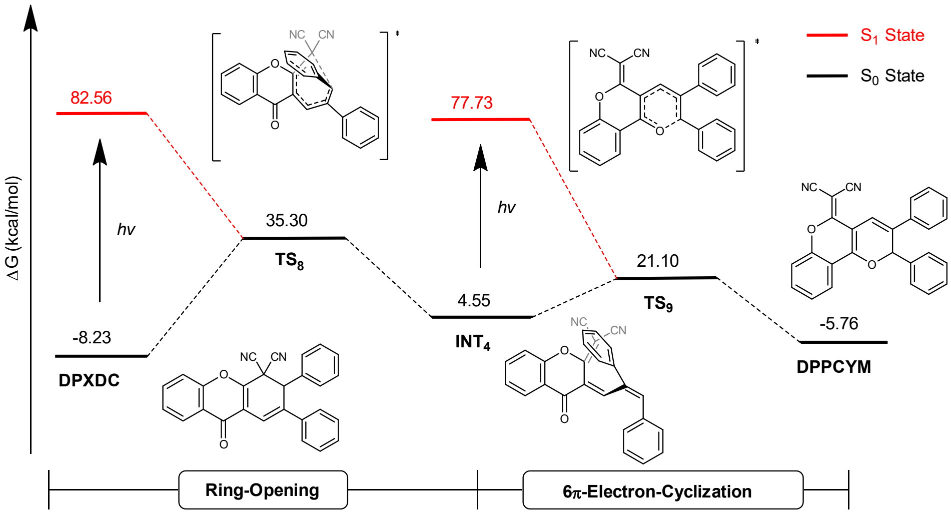

Supplementary Fig. 58 Energy profile for the photoarrangement of DPXDC calculated at the M06-2X/6-31G(d,p) level without solvent correction. TS transition state, INT intermediate.

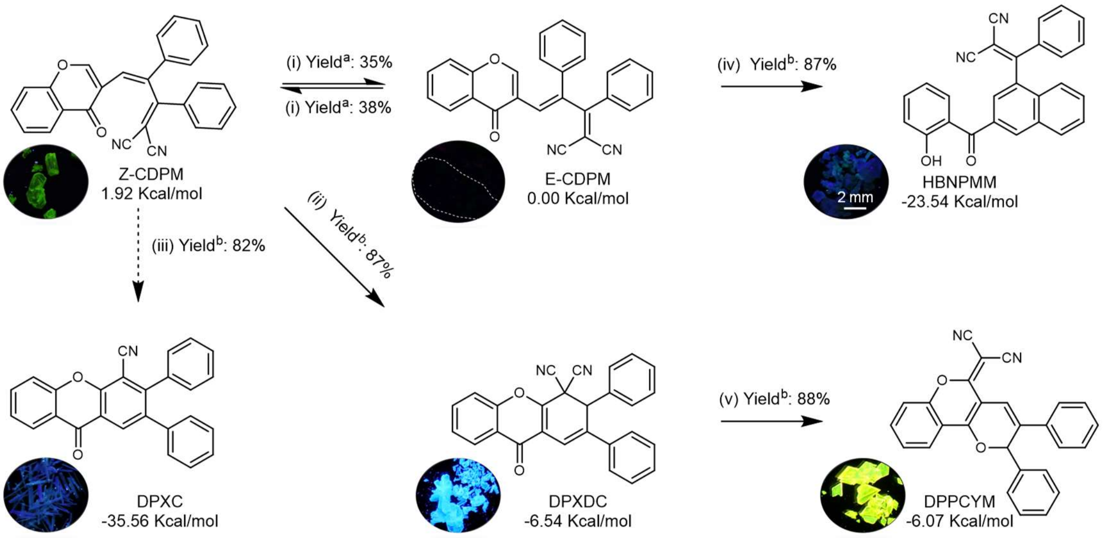

Supplementary Fig. 59 The thermal and photo reactions of *Z*-CDPM under different conditions: (i) annealing at 145 °C in $C_2D_2Cl_4$ for 1 d; (ii) annealing at 200°C in diphenyl ether for 2 h; (iii) annealing at 145 °C in silica gel for 1 d; (iv, v) UV irradiation under a 365 nm UV lamp (0.12 mW/cm^2) at room temperature in $C_2D_2Cl_4$ for 2 h. Insets: photographs of crystals taken under UV irradiation and the relative Gibbs free energies of all six ground-state isomers. [a] Conversion yield determined by ^{1}H NMR data. [b] Conversion yield determined by isolation of products. Scale bar: 2 mm.

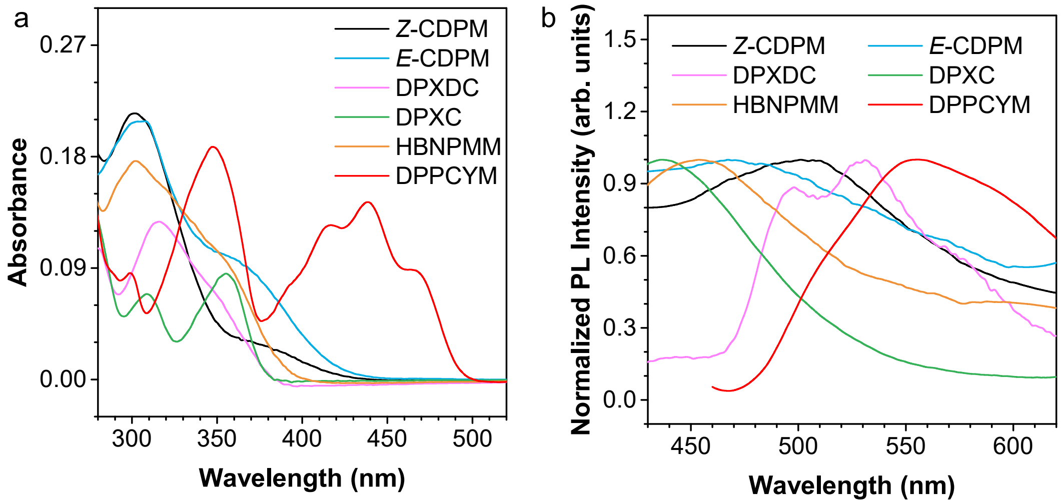

Supplementary Fig. 60 (a) Absorption of AIEgens (10 μM) in THF solution. (b) Normalized Photoluminescence (PL) spectra of AIEgens (10 μM) in THF/water mixtures (f_w = 99%).

Supplementary Table 6. Summary of photophysical properties of Z-CDPM and its isomers.

Compound	λ_{ab} [nm][a]	ε [$M^{-1}\cdot cm^{-1}$]	λ_{em} [nm][b]	Quantum yield [%]	
				Aggregate[c]	Crystal
Z-CDPM	302 (π→π*)	21497	505	1.0	2.1
E-CDPM	308 (π→π*)	20867	480	0.2	0.5
DPXDC	315 (π→π*)	12730	495	8.2	14.9
DPXC	355 (π→π*)	8560	440	3.3	2.8
HBNPMM	303 (π→π*)	17634	460	1.0	0.9
DPPCYM	440 (π→π*)	14269	550	4.5	37.8

[a] Absorption maximum was measured in THF solution. Concentration= 10 μM. [b] Emission maximum was measured in THF/water mixture (f_w =99%). Concentration= 10 μM. [c] Quantum yield was measured in in THF/water mixture (f_w =99%).

Supplementary Table 7. Summary of the photochromic and thermochromic parameters of luminogens reported in this work and other classic systems.

Source	Luminogen name: initial state	Luminogen name: transformation	stimuli	response time	energy barrier (Kcal/mol)	Conversion efficiency (%)
This work	Z-CDPM	E-CDPM	145 ℃	24 h	27.87	35
	Z-CDPM	DPXDC	200 ℃	2 h	36.54	87
	Z-CDPM	DPXC	145 ℃	6 h	61.01	82
	Z-CDPM	HBNPMM	365 nm	10 s	88.43	87
	DPXDC	DPPCYM	365 nm	10 s	89.70	88
	E-CDPM	Z-CDPM	145 ℃	24 h	29.78	38
Ref. 18	Z-MPPMNAN	E-MPPMNAN	365 nm	96 h	65.64	76
	Z-MPPMNAN	c-MPPMNAN	365 nm	70 h	N.A.	> 90
	Z-MPPMNAN	d-MPPMNAN	365 nm	48 h	69.89	> 90
	E-MPPMNAN	Z-MPPMNAN	75 ℃	36 h	N.A.	> 90
Ref. 32	TPE-SP1-OF	TPE-SP1-CF	254 nm	6 min	N.A.	> 90
	TPE-SP1-CF	TPE-SP1-OF	500 nm	N.A.	N.A.	N.A.
Ref. 35	1a	1b	366 nm	10 s	N.A.	> 90
	1b	1a	450 nm	40 s	N.A.	> 90
Ref. 37	DHAP-H	VHFP-H	365 nm	15 s	81.40	> 90
	VHFP-H	DHAP-H	60 ℃	2 h	N.A.	> 90
Ref. 41	NS	NSI	365 nm	8 h	60.66	55

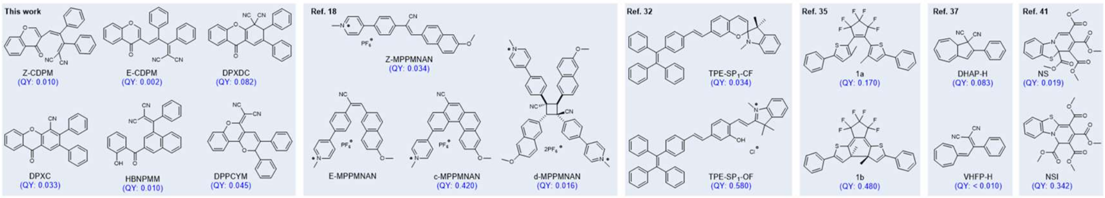

Abbreviation: N.A., not available.

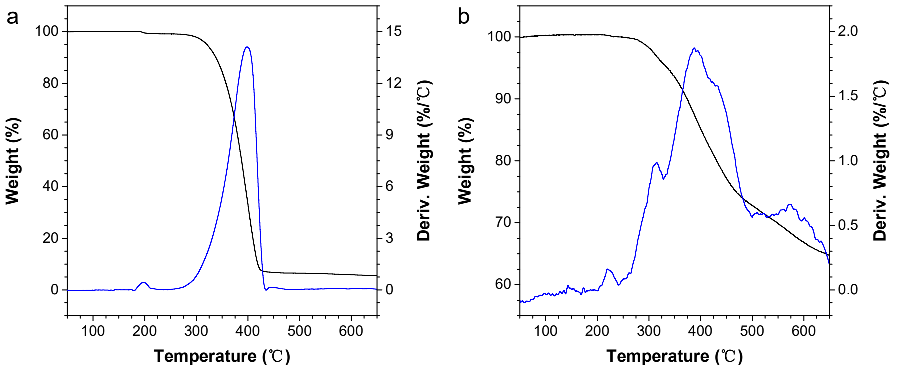

Supplementary Fig. 61 Thermogravimetric analysis diagrams of HBNPMM (a) and DPPCYM (b) recorded under nitrogen at a heating rate of 10 °C/min.

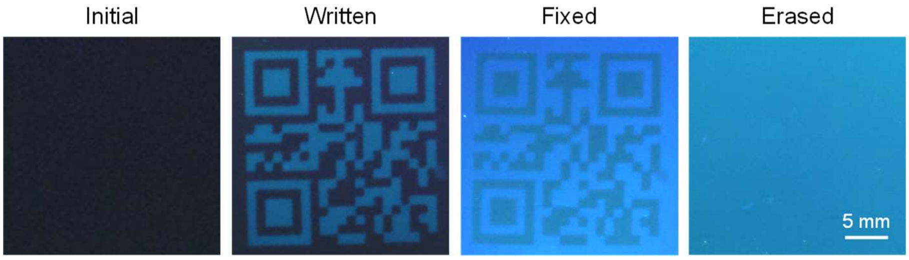

Supplementary Fig. 62 Photographs of the developed colored quick response code of *Z*-CDPM. Scale bar: 5 mm.

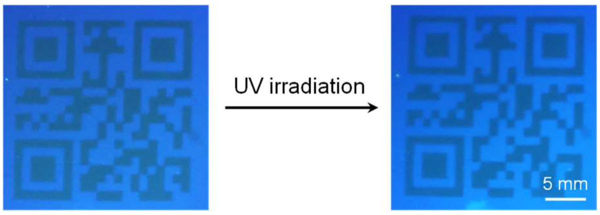

Supplementary Fig. 63 Photographs of the fixed colored quick response code of *Z*-CDPM before and after UV irradiation. Scale bar: 5 mm.

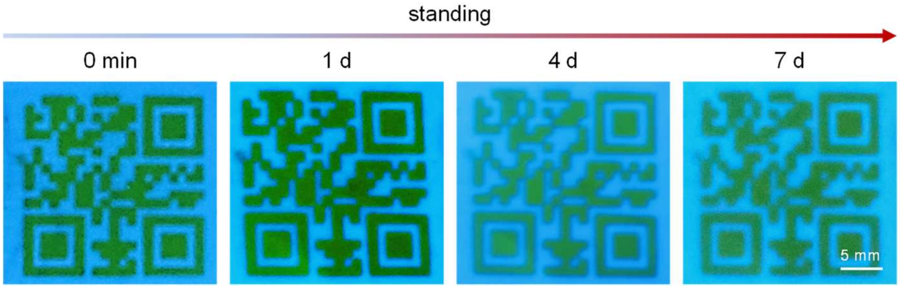

Supplementary Fig. 64 Photographs of the developed colored quick response code of DPXDC after standing at room temperature for different times (0, 1, 4, 7 d). Scale bar: 5 mm.
