# Supplementary material for: A chameleon AIEgen exhibiting six distinct yet tunable thermal and photoswitchable states
Source: Nat Commun. 2025 Jul 9;16:6312. doi: 10.1038/s41467-025-61717-x (PMC12238559; doi:10.1038/s41467-025-61717-x)
Supplement: Supplementary file 4 — Transparent Peer Review file [file 41467_2025_61717_MOESM4_ESM.pdf]

nature portfolio

Peer Review File

A chameleon AIEgen exhibiting six distinct yet tunable thermal and photoswitchable states

Corresponding Author: Professor Ben Zhong Tang

This file contains all reviewer reports in order by version, followed by all author rebuttals in order by version.
Parts of this Peer Review File have been redacted as indicated to remove third-party material.

Version 0:

Reviewer comments:

Reviewer #1

(Remarks to the Author)
This manuscript reports a novel multiresponsive AIEgen (Z-CDPM) with six distinct, tunable thermal and photoswitchable states, and explores its application in information encryption. Notably, the structures of all these six compounds were successfully confirmed by the single-crystal X-ray diffraction. In particular, the successful demonstration of multiple switches between different states originating from a single molecule is both interesting and innovative, opening new avenues for the design of multifunctional luminogens. Given these excellent properties, such as multiple responsiveness and precise controllability, these molecules have been effectively applied to create multicolored images, quick response codes, and an advanced multicolor encryption system.
Overall, the manuscript is well-written and concise, with sufficient characterization and mechanism explanations. Therefore, in my opinion, it can be accepted after minor revisions, provided that the following issues are thoroughly addressed.

1) The authors state that DPXDC exhibits AIE properties. However, in Fig. 3e, as the water fraction increases, the emission peak shifts to longer wavelengths and changes from a single peak to two peaks when the water fraction reaches fw = 90%. Considering that DPXDC can undergo a photoarrangement to DPPCYM, is it possible that the emission spectra at fw = 90% and fw = 99% do not originate from pure DPXDC? The long-wavelength peak around 530 nm may be from DPPCYM, which would be consistent with the images shown in Fig. 7a.
2) In the DFT calculated thermal elimination mechanism shown in Fig. 4e, could INT 1 undergo a direct one-step HCN elimination to generate DPXC? Why is an additional [1,5]-H shift required? A direct HCN elimination seems more reasonable. Have the authors considered or tested this alternative mechanism?
3) Several formatting issues need to be addressed for clarity and uniformity. In lines 142 and 158–159, the first letter of the abbreviation "Z-CDPM" is not italicized. The journal names in References 9 and 22 have not been abbreviated. The dash lines between reference page numbers should be standardized, ensuring the consistent use of en-dashes; for example, a hyphen has been incorrectly used in Reference 24. Additionally, references 18, 32, 37, and 47 vary in whether they include issue numbers, which should be made consistent. Please carefully review and correct any similar formatting errors.
4) The terms "photo-arrangement" and "photoarrangement" are used interchangeably throughout the manuscript. For consistency, it’s recommended to standardize the terminology across the whole manuscript.
5) In Fig. 1, the authors state that DPXDC can be obtained by annealing Z-CDPM at 145°C in C2D2Cl4 for 30 days. However, this is an extremely long duration, making the synthetic route costly and time-consuming. Meanwhile, the main text indicates that DPXDC can also be synthesized in just 2 hours by heating Z-CDPM in diphenyl ether at 200°C. Therefore, it would be more appropriate to avoid emphasizing the inefficient 30-day method in Fig. 1 and instead highlight the more practical and time-efficient synthetic approach.
6) An intriguing question is why annealing Z-CDPM at 145 ℃ in $C_2D_2Cl_4$ for 30 days results in DPXDC, whereas annealing Z-CDPM at the same temperature in silica gel for just one day leads to DPXC. Do the authors have any explanation for this outcome?

Reviewer #2

(Remarks to the Author)
The study presents a novel chromone-based aggregation-induced emission (AIE) luminogen, Z-CDPM, which exhibits five distinct thermally and photochemically induced transformations, leading to six different states. The manuscript aims to

establish this system as a promising candidate for stimuli-responsive materials with potential applications in multicolor imaging and information encryption. While the work is scientifically intriguing and provides valuable insights into multiresponsive luminogens, several critical concerns must be addressed before the manuscript can be considered for publication.
Major Concerns

1. Mechanistic Rigor and Validation

The manuscript proposes that Z-CDPM undergoes five switching processes, including reversible Z/E isomerization, thermal cyclization, thermal elimination, and two photorearrangements. However, the experimental evidence supporting these pathways remains incomplete:
• The Z/E isomerization is described based on NMR studies, but quantitative kinetic data is absent. What is the rate constant for isomerization under different conditions? Are there alternative pathways that could contribute to the observed conversion?
• The thermal cyclization process (Cy-Type A) is proposed to proceed via an intermediate, but this intermediate has not been directly characterized. Have the authors considered in situ spectroscopic monitoring (e.g., time-resolved NMR, UV-Vis absorption changes) to track its formation?
• The thermal elimination mechanism suggests that silica gel facilitates the process, potentially by promoting HCN elimination. However, no experimental evidence (e.g., mass spectrometry to confirm volatile byproducts) is provided. Can the authors explicitly demonstrate HCN evolution through gas chromatography or another method?

2. Insufficient Comparative Analysis with Existing Systems

The authors claim that Z-CDPM represents an exceptional example of a multiresponsive luminogen. However, the lack of direct comparison with previously reported photochromic and thermochromic systems weakens this claim.
• How does the switching efficiency of Z-CDPM compare to stilbene-based, diarylethene, or spiropyran systems, which have well-characterized reversible photochromic behavior?
• The study should quantitatively compare its fluorescence quantum yields and switching kinetics with other AIE-based stimuli-responsive luminogens reported in recent literature.
• A table summarizing existing materials, their response times, energy barriers, and photostability would significantly strengthen the manuscript's argument regarding Z-CDPM's advantages.

3. Data Presentation and Interpretation

Several aspects of data presentation require improvement to enhance the manuscript's clarity and scientific rigor:
• Figures
• Figure 1b: The reaction conditions (temperature, solvent, light intensity) should be explicitly stated within the figure or its caption.
• Figures 3–6: The photophysical spectra are difficult to interpret due to overlapping signals and unclear baseline corrections. Improved annotation and curve fitting would enhance clarity.
• Figure 7 (QR code applications): While visually appealing, the lack of quantitative discussion on long-term stability and reproducibility under real-world conditions is a major limitation.
• Spectroscopic Data
• The NMR spectra provided for structural characterization are not sufficiently detailed. Enlarged views of key chemical shifts and integration values should be included to facilitate independent verification.
• The UV-Vis and PL spectra lack a clear discussion of peak assignments. What electronic transitions correspond to the observed spectral shifts?
• Computational Analysis
• The DFT calculations provide valuable energy profiles but lack solvent effects, which may be significant for photochemical and thermal processes. Have the authors considered solvent-corrected energy calculations?
• The absence of excited-state dynamics simulations raises concerns about the accuracy of the proposed photochemical mechanisms.

Minor Concerns
• Language and Clarity
• Several sections, particularly in the Introduction and Results, contain redundant phrasing and overly long sentences. Improving conciseness would enhance readability.
• The term "quintuple switching" is not commonly used in the field. A clearer phrase, such as "five distinct and controllable transformations," may be more appropriate.
• References
• The manuscript extensively cites recent work in AIE materials, which is commendable. However, some key historical references on photochromism and thermal isomerization (e.g., seminal work on spiropyrans and diarylethenes) are missing and should be incorporated.

Based on the concerns outlined above, I recommend a Major Revision before the manuscript can be considered for publication.

Reviewer #3

(Remarks to the Author)
In this work He et al. describe a luminescent molecular system consisting of six different states derived from a single molecular framework. Upon different heat (temperature) and light stimuli the six different states are selectively accessible when using suitable combinations of the stimuli. One heating conversion is reversible to some degree. All six states show different luminescence and also AIE behavior both in solution and on solid support, which allows the authors to use them in multi-level stimuli responsive applications, for example in encryption, optical memories and information inscription/erasure. The structural identity of the six different isomers is convincingly elucidated by single-crystal X-ray diffraction and the individual conversions between isomers seem to be of very high conversion as well in each case. The authors also deliver a quantum chemical description of the states as well as conversion barriers and thus gained first insights into the isomerization mechanisms.
Overall I believe that this work certainly has the potential to be publishable in a high-impact journal such as Nature Communcations. The molecular system reported is novel and unique and shows very good conversion between the states and correlated significant changes of luminosity. The authors make full use of this complex behavior in a number of elevated photonic applications, so in general novelty and performance really seem to be excellent.
However, there are a number of issues that need to be addressed by the authors in order to accurately describe their system and elucidate the performance properties. With respect to theory, there are also a number of points that need addressing.

Major points:

- First, not all transformations are clearly quantified. Please provide yields for every single isomer transformation, such that these six conversions can actually be compared correctly and also report how these yields were determined (NMR, isolation of products and balancing…). From the NMR traces shown, it seems that these conversions are in fact very high and also the authors say that they prepare individual isomers synthetically using the heat/irradiation steps.
- In addition to that, I miss an overview Figure, which shows a) photographs of all solid materials and solutions to be able to show the oftentimes cited “coloration” or “decoloration” in the manuscript text. The same was already done for fluorescence in Figure 7a. Along with it, molar absorptions (extinction coefficients) for all six isomers need to be shown side by side in one absorption diagram as well as the corresponding fluorescence diagrams taken at the same concentrations of samples.
- Second, there is an issue with the analysis of the thermal reactions and the interpretations thereof. When heating the Z/E CDPM isomers they do interconvert into each other at 145 °C but also further conversion to the DPXDC isomer starts to occur. This is seen in Fig. 3a for example. Kinetic modeling is needed here to distinguish different possible scenarios but also to deliver actual experimental Gibbs energies of activations for these processes to compare with the quantum chemical modelling. Two scenarios are possible at least: it could be 1. that ZCDPM is going to ECDPM first and then ECDPM is going into DPXDC. Or 2. ZCDPM is branching into ECDPM and DPXDC to some degree. However clearly ECDPM is going into DPXDC!
- A related issue is the apparent equilibration between Z and E CDPM. It looks like they do reach almost 1:1 ratio before significant further thermal reactions occur. This is important as it shows that the DFT calculation giving 3.4 kcal/mol energy difference is not correct. The authors say that this is very low energy difference, but at such a value the ratio between E and Z should be around 98%:2% at 145 °C, which is not observed experimentally. This hints already at some problems in the theory.
- Further, saying there is a control for the process of Z to E or E to Z isomerization in CDPM is overstating because that is simply an equilibration and it does apparently not lead to significant accumulation of one isomer – so that is also not really usable to change any luminescent properties.
- On the same note: the equilibrium isomer distribution Z/E needs to be assessed in Figure 2f the isomers are not equilibrated when starting from either Z or E. the equilibrium distribution would directly give the delta G value between the two isomers from experiment to compare to DFT.
- The authors claim an endothermal process when going from Z/E CDPM to DPXDC. but the cyclized product is more stable. So that is certainly not an endergonic process- do the authors suggest an entropy driven reaction or how do they interpret their data correctly?
- I am also not sure that the observe different heating behavior in solution and on silica solid support is really caused by the “morphology changes”. It could well be the acidity of the environment. Please test whether pH changes in solution lead to different thermal and photochemical outcomes of the different isomers. Also test if basic alumina support give the same results.
- On the same note, with proper choice of solvent many thermal processes could be influenced dramatically giving the possibility to obtain better control and introducing reversibility for cyclization reactions. Such effects are well known in photochromes for example see here: doi: 10.1038/s41467-019-12463-4; doi: 10.1021/jacs.1c11183; doi:10.1021/jacs.4c06377 I would urge the authors to assess highly polar versus completely apolar solvents for that purpose.
- With respect to the calculations: DFT values for Gibbs energies of activation also do not align with the experiments, not even qualitatively. In the experiment E to Z and Z to E isomerizations are faster than the follow up cyclization reactions. Thus, the order of barrier heights is upside down in the DFT calculations. Generally the calculated barriers are oftentimes extremely high beyond 45 kcal/mol, which would not allow to do these transformations at reasonable times even at temperatures of 145 -200°C. please calculate via the Eyring equation how long the heating processes would take based on the DFT barriers and compare to the actual experiments. For sake of simplicity you can assume first order rate laws.
- Further what is odd is that the authors use the E-isomer as point 0 and thus obtain negative delta G for the cyclized isomers. Please use the global minimum as 0 to compare all isomers properly. Please also provide a scheme where all minima of the six isomers are compared side by side.
- Generally when talking about AIE, from the alpha-values it is not clear how good the AIE performance is. please show quantitative data- what is the intensity increase factor? How is alpha AIE defined? is the concentration always the same or is

that dilution (by what factor) of THF solution with water?
- Also generally the luminescence data and AIE behaviors are oftentimes presented without numerals on the y-axes of the diagrams in most Figures throughout the manuscript and the SI. Please add the corresponding numbers! Quantify at least relatively the AIE increase in luminosity and also the luminosity changes between isomers in solution and solid states.

- Important literature on multi-stimuli responsive systems is missing, that need to be cited to put this work in proper context. For example, multi-photochromes are a large developed field already and important early work is not cited at all here: a) A. Fihey, A. Perrier, W. R. Browne, D. Jacquemin, Chem. Soc. Rev. 2015, 44, 3719–3759; b) H. Nie, J. L. Self, A. S. Kuenstler, R. C. Hayward, J. Read de Alaniz, Adv. Opt. Mater. 2019, 7; c) D. Gust, J. Andreasson, U. Pischel, T. A. Moore, A. L. Moore, Chem. Commun. 2012, 48, 1947–1957; d) J. Andreasson, U. Pischel, Chem. Soc. Rev. 2018, 47, 2266–2279; e) X. Fu, L. Hosta-Rigau, R. Chandrawati, J. Cui, Chem 2018, 4, 2084–2107; f) M. Wei, Y. Gao, X. Li, M. J. Serpe, Polym. Chem. 2017, 8, 127–143. g) J. Andreasson, U. Pischel, S. D. Straight, T. A. Moore, A. L. Moore, D. Gust, J. Am. Chem. Soc. 2011, 133, 11641–11648; h) F. Tian, D. Jiao, F. Biedermann, O. A. Scherman, Nat. Commun. 2012, 3, 1207; i) M. Herder, M. Utecht, N. Manicke, L. Grubert, M. Pätzel, P. Saalfrank, S. Hecht, Chem. Sci. 2013, 4, 1028–1040; j) M. Li, S. Yang, W. Liang, X. Zhang, D. Qu, Dyes Pigm. 2019, 166, 239–244; e) T. Schmitt, L. Y. Hsu, N. Oberhof, D. Rana, A. Dreuw, E. Blasco, P. Tegeder, Adv. Funct. Mater. 2023; k) J. Andréasson, S. D. Straight, T. A. Moore, A. L. Moore, D. Gust, J. Am. Chem. Soc. 2008, 130, 11122–11128; l) H. Nishioka, X. Liang, T. Kato, H. Asanuma, Angew. Chem. Int. Ed. 2012, 51, 1165–1168; m) H. Dube, J. Rebek Jr., Angew. Chem. Int. Ed. 2012, 51, 3207–3210; n) K. Hiltebrandt, T. Pauloehrl, J. P. Blinco, K. Linkert, H. G. Borner, C. Barner-Kowollik, Angew. Chem. Int. Ed. 2015, 54, 2838–2843; j) M. Skugor, J. Valero, K. Murayama, M. Centola, H. Asanuma, M. Famulok, Angew. Chem. Int. Ed. 2019, 58, 6948–6951; o) V. X. Truong, J. Bachmann, A. N. Unterreiner, J. P. Blinco, C. Barner-Kowollik, Angew. Chem. Int. Ed. 2022, 61, e202113076; p) D. Manna, T. Udayabhaskararao, H. Zhao, R. Klajn, Angew. Chem. Int. Ed. 2015, 54, 12394–12397; q) F. Zhao, L. Grubert, S. Hecht, D. Bleger, Chem. Commun. 2017, 53, 3323–3326; r) M. Drohse Kilde, L. Brolos, M. Manso, J. Mogensen, C. Gregers Tortzen, M. Brondsted Nielsen, Chem. Eur. J. 2020, 26, 13429–13435; s) M. Dowds, S. G. Stenspil, J. H. de Souza, B. W. Laursen, M. Cacciarini, M. B. Nielsen, ChemPhotoChem 2022, 6; t) M. M. Lerch, M. J. Hansen, W. A. Velema, W. Szymanski, B. L. Feringa, Nat. Commun. 2016, 7, 12054; u) N. M. Wu, M. Ng, V. W. Yam, Nat. Commun. 2022, 13, 33; r) A. Gerwien, B. Jehle, M. Irmler, P. Mayer, H. Dube, J. Am. Chem. Soc. 2022, 144, 3029–3038; s) G. R. Wilson, K. C. Park, G. C. Thaggard, C. R. Martin, A. R. Hill, J. Haimerl, J. Lim, B. K. P. Maldeni Kankanamalage, B. J. Yarbrough, K. L. Forrester, R. A. Fischer, P. J. Pellechia, M. D. Smith, S. Garashchuk, N. B. Shustova, Angew. Chem. Int. Ed. 2023, 62, e202308715; v) J. Sheng, J. Perego, S. Bracco, P. Cieciorski, W. Danowski, A. Comotti, B. L. Feringa, Angew. Chem. Int. Ed. 2024, e202404878. w) Köttner, Dube, Angew. Chem. Int. Ed. 2024, 63, e202409214
- When talking about ring-cyclization and in particular ring flipping reactions there also needs mentioning of recent works of aza-diarylethenes with multi-state behavior in such "flipping" manner:
doi:10.1021/jacs.3c11803;doi:10.1002/adma.202411223; doi:10.1002/anie.202415961
- The authors use inflated language quite a bit and I would urge them to tone down their claims, oftentimes the used terms are either not scientific or seem to be used just for buzz-word sake. Examples are: "energy isolation" to describe selectivity in thermal steps that are simply based on sufficiently different Gibbs energies of activation for these processes; "fantastic ring switching"; "morphology tuning" to describe the switching on solid support instead of switching in solution; "advanced artificial intelligence technologies" there is no clear connection to AI with such work…

Minor points:

- "Subsequently, the fantastic ring-switching" please tone down
- "Photophysical properties and Z/E isomerization. Z-CDPM and E-CDPM were simultaneously synthesized via a facile two-step process" please state here whether you have isolated Z and E and studied them separately or if that was done in mixtures… - also make that clear when talking about crystal structures.
- Fig. 2 a and b the actual isomerization is hard to see from the chemdraw structures. Please leave one fragment static and show the isomerization that way.
- "When a poor solvent of water was added…" what does that mean?
- "Z/E isomerization was demonstrated for CDPM, as well as the similar thermodynamic stability of two isomers" again 3.4 kcal/mol is not really similar and would lead to more than 95% isomer accumulation under thermal equilibrium even at very high temperatures.
- "As the reaction energy (ΔG = 8.09 kcal·mol-1) was similar to that of E-CDPM (ΔG = 7.43 kcal·mol-1), the comparable thermodynamic stability of these two isomers was confirmed again." please state here explicitly, that you are talking about Z and E CDPM.
- What is the yield of DPXC formation?
- Please state for all figures where DFT calculations are presented that the energy profiles are theoretical and also state the level of theory in the figure captions and in the manuscript text.
- "Following, the photophysical properties of DPPCYM and the fluorescence response of Z-CDPM to UV irradiation were investigated." I believe the authors meant DPXDC instead of Z-CDPM?
- "Indeed, the specific photophysical properties of DPXDC further confirmed the feasibility of performing discoloration in a single-luminogen system through the ring-flipping process." why discoloration? is not the product DPPCYM orange as stated above?
- "On the other hand, a gradually enhanced emission was detected for the UV irradiated sample of DPXDC in THF/water mixture with a fw of 99% (Fig. 6d)." how much? is not DPXDC also an AIEgen as stated above? Please provide quantitative comparison of the AIE properties of all isomers as requested in the major part above.
- "As the subsequent excitation energy (73.18 kcal/mol) and energy barrier (21.10 kcal/mol) were much lower than that of the former ring-opening process (90.79 kcal/mol for the excitation energy and 35.30 kcal/mol for energy barrier), INT4 would rapidly convert to DPPCYM through cyclization with the carbonyl group." This is not an argument, as photochemical

processes in the excited state are not governed by excitation energy or ground state barriers per se. I would omit that sentence.
- The authors use terms like “AIE plate” or “AIE template” to describe their molecules on silica support. It is not clear if the behavior is really AIE related in these cases because luminescence could also be caused by motion restriction via interactions between solid support and the molecules instead of aggregation effects. Just replace those terms by “on solid support” or likewise.
- Fig. 7 please state in every instance what is the state (starting, after stimuli are given) and also state what conditions were applied where. Either in the caption or in the Figures themselves. Otherwise it is not clear which state transformations are used in each instance.
-

Version 1:

Reviewer comments:

Reviewer #1

(Remarks to the Author)
I believe the manuscript can be published as its.

Reviewer #2

(Remarks to the Author)
The authors have responded to all of my comments point-to-point and revised the manuscript accordingly. The scientific issues I raised have been adequately addressed, and the overall clarity and rigor of the manuscript have been significantly improved. I believe the current version meets the standards for publication, and I recommend that the manuscript be accepted in its current form.

Reviewer #3

(Remarks to the Author)
In their revision the authors have thoroughly answered to all the points raised by this reviewer. They have provided new experimental data as well as computations and have managed to improve the manuscript and the SI significantly compared to their initial submission. The new data show the robustness of the individual transformations and the photochemical and photophysical properties and the agreement between the theoretical description and the experiments is now much improved as well. For these reasons I now support publication of this work in Nature Communcations.

Open Access This Peer Review File is licensed under a Creative Commons Attribution 4.0 International License, which permits use, sharing, adaptation, distribution and reproduction in any medium or format, as long as you give appropriate credit to the original author(s) and the source, provide a link to the Creative Commons license, and indicate if changes were made.

In cases where reviewers are anonymous, credit should be given to 'Anonymous Referee' and the source.
The images or other third party material in this Peer Review File are included in the article's Creative Commons license, unless indicated otherwise in a credit line to the material. If material is not included in the article's Creative Commons license and your intended use is not permitted by statutory regulation or exceeds the permitted use, you will need to obtain permission directly from the copyright holder.
To view a copy of this license, visit https://creativecommons.org/licenses/by/4.0/

REVIEWER COMMENTS

Response to the Comments of Reviewer 1:

This manuscript reports a novel multiresponsive AIEgen (Z-CDPM) with six distinct, tunable thermal and photoswitchable states, and explores its application in information encryption. Notably, the structures of all these six compounds were successfully confirmed by the single-crystal X-ray diffraction. In particular, the successful demonstration of multiple switches between different states originating from a single molecule is both interesting and innovative, opening new avenues for the design of multifunctional luminogens. Given these excellent properties, such as multiple responsiveness and precise controllability, these molecules have been effectively applied to create multicolored images, quick response codes, and an advanced multicolor encryption system.
Overall, the manuscript is well-written and concise, with sufficient characterization and mechanism explanations. Therefore, in my opinion, it can be accepted after minor revisions, provided that the following issues are thoroughly addressed.
Response: We appreciate the positive assessment from the reviewer. We have revised the manuscript according to your valuable suggestions.

1) The authors state that DPXDC exhibits AIE properties. However, in Fig. 3e, as the water fraction increases, the emission peak shifts to longer wavelengths and changes from a single peak to two peaks when the water fraction reaches fw = 90%. Considering that DPXDC can undergo a photoarrangement to DPPCYM, is it possible that the emission spectra at fw = 90% and fw = 99% do not originate from pure DPXDC? The long-wavelength peak around 530 nm may be from DPPCYM, which would be consistent with the images shown in Fig. 7a.
Response: Thanks a lot for your careful review and comment. We fully agree with the reviewer that the emission spectra of DPXDC at high water fraction (f_w) do not originate from pure DPXDC. As depicted in Fig. 6d, DPXDC undergo a fast photoarrangement to form DPPCYM in solution, and it requires about 10 s to reach a plateau. Also, DPPCYM shows strong emission peaked at 550 nm at both f_w = 90% and 99% (Supplementary Fig. 22). Since the PL spectrum of DPXDC is taken under UV irradiation, and the long-wavelength peak at 535 nm in Fig. 3e are very close to that of DPPCYM. Conceivably, this peak should be attributed to the small amount of DPPCYM generated during the excitation process, and it would red-shifted to 550 nm with continuous irradiation to generate large amount of DPPCYM. Moreover, the images shown in Fig. 3c and Fig. 7a confirmed the emission color of blue for DPXDC in solid state as well. Thus, to better convey this point, we have added the sentence "*Previously, a long-wavelength peak at around 535 nm was detected for DPXDC at f_w = 90% and 99% under 365 nm excitation (Fig. 3e), which was similar to that of DPPCYM. Combined with the fast photoarrangement of DPXDC in solution, this signal at 535 nm would be attributed to the generation of DPPCYM upon UV excitation*" in the main text.

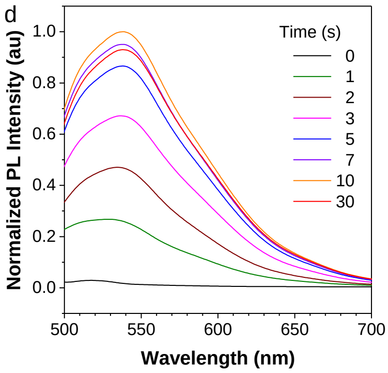

Fig. 6d PL spectral of DPXDC before and after UV irradiation at 365 nm for different times (1, 2, 3, 5, 7,10, and 30 s). λ_{ex} = 440 nm.

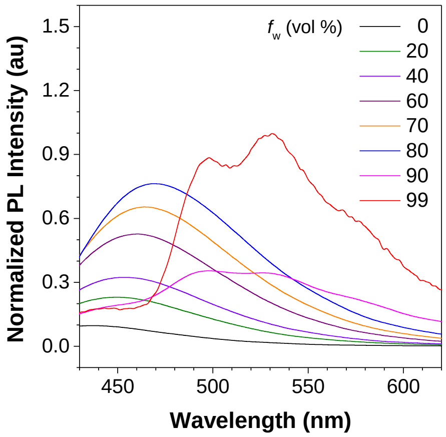

Supplementary Fig. 22 PL spectra of DPXDC (10 μM) in THF/water mixtures. λ_{ex} = 365 nm.

2) In the DFT calculated thermal elimination mechanism shown in Fig. 4e, could INT 1 undergo a direct one-step HCN elimination to generate DPXC? Why is an additional [1,5]-H shift required? A direct HCN elimination seems more reasonable. Have the authors considered or tested this alternative mechanism?

Response: Thanks a lot for this important comment. According to your valuable suggestions, we have evaluated the energy profile of this alternative mechanism and added the results in Supplementary Fig. 36 in the supporting information. Our calculations indicate that INT1 is unlikely to undergo a direct, one-step HCN elimination to generate DPXC, as this pathway involves a prohibitively high energy barrier of 80.56 kcal/mol. In contrast, the [1,5]-H shift from INT1 to form DPXDC requires a significantly lower barrier of 36.01 kcal·mol^{-1}, and the resulting DPXDC intermediate is energetically more stable (–6.54 kcal/mol) than INT1 (2.60 kcal/mol). The subsequent HCN elimination from DPXDC proceeds via a barrier of 61.01 kcal/mol, which is also lower than that of the direct concerted elimination pathway (80.56 kcal/mol). These results suggest that a stepwise mechanism involving an initial [1,5]-H shift followed by HCN elimination is more favorable than a concerted process. To reflect this finding, we have added Supplementary Fig. 36 in the supporting information and also the following sentence in the main text to the revised manuscript: "*Since INT1 needed to overcome a higher energy barrier of 80.56 Kcal/mol than that of DPXDC (61.01 Kcal/mol) to generate DPXC, theoretically it would get over a lower energy barrier of [1,5]-H shift (36.01 Kcal/mol) to form DPXDC first*".

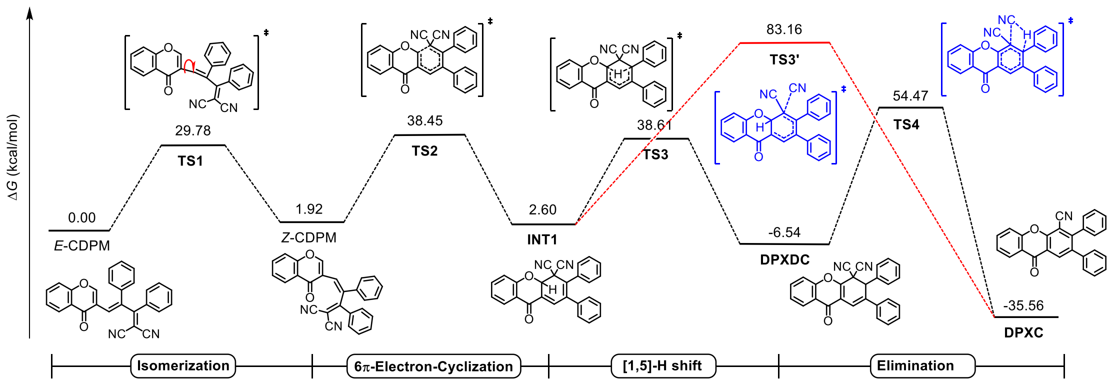

Supplementary Fig. 36 Energy profile for the thermal reactions of *Z*-CDPM calculated at the M06-2X/6-31G(d,p) level with solvent correction.

3) Several formatting issues need to be addressed for clarity and uniformity. In lines 142 and 158–159, the first letter of the abbreviation "Z-CDPM" is not italicized. The journal names in References 9 and 22 have not been abbreviated. The dash lines between reference page numbers should be standardized, ensuring the consistent use of en-dashes; for example, a hyphen has been incorrectly used in Reference 24. Additionally, references 18, 32, 37, and 47 vary in whether they include issue numbers, which should be made consistent. Please carefully review and correct any similar formatting errors.

Response: Thanks a lot for your careful review and comments. We have corrected the corresponding formatting errors and other similar errors in the main text according to your good suggestions. Specifically, the abbreviation "*Z*-CDPM" are all italicized, the journal names are abbreviated, the dash lines have been standardized, and the issue numbers are corrected. We are very grateful for the detailed suggestions of the reviewers.

4) The terms "photo-arrangement" and "photoarrangement" are used interchangeably throughout the manuscript. For consistency, it's recommended to standardize the terminology across the whole manuscript.

Response: Thanks a lot for your careful review and comments. We have replaced "photo-arrangement" with "photoarrangement" in the main text to standardize the terminology according to your valuable suggestion.

5) In Fig. 1, the authors state that DPXDC can be obtained by annealing Z-CDPM at 145°C in $C_2D_2Cl_4$ for 30 days. However, this is an extremely long duration, making the synthetic route costly and time-consuming. Meanwhile, the main text indicates that DPXDC can also be synthesized in just 2 hours by heating Z-CDPM in diphenyl ether at 200°C. Therefore, it would be more appropriate to avoid emphasizing the inefficient 30-day method in Fig. 1 and instead highlight the more practical and time-efficient synthetic approach.

Response: Thanks a lot for your careful review and comment. We fully agree that the generation of DPXDC by annealing *Z*-CDPM at 145° C in $C_2D_2Cl_4$ is an extremely long duration, and the reaction condition of 2h in diphenyl ether at 200° C is more practical. According to your good suggestion, we have changed the description into “annealing at 200°C in diphenyl ether for 2 h” in Fig. 1 to better convey the reaction conditions to reviews.

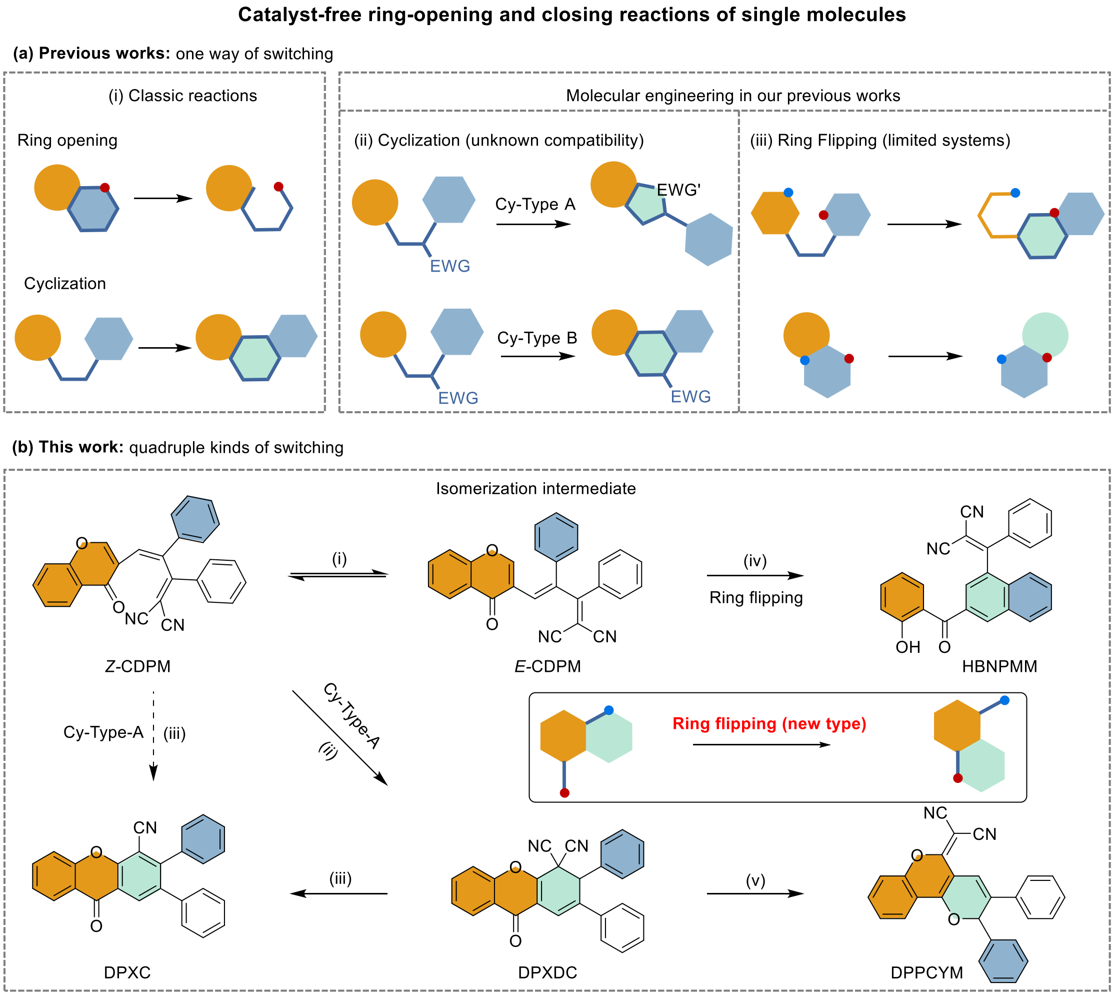

Fig. 1 Design strategy of molecules with multiple responses and controls. (a) Conventional catalyst-free ring-opening and closing reactions in monomolecular systems and their characteristics. (b) Schematic illustration of the thermal and photo reactions of *Z*-CDPM under different conditions: (i) annealing at 145 °C in $C_2D_2Cl_4$ for 1 d; (ii) annealing at 200°C in diphenyl ether for 2 h; (iii) annealing at 145 °C in silica gel for 1 d; (iv, v) UV irradiation under a 365 nm UV lamp (0.12 mW/cm^2) at room temperature in $C_2D_2Cl_4$ for 2 h.

6) An intriguing question is why annealing Z-CDPM at 145 °C in $C_2D_2Cl_4$ for 30 days results in DPXDC, whereas annealing Z-CDPM at the same temperature in silica gel for just one day leads to DPXC. Do the authors have any explanation for this outcome?

Response: We appreciate the reviewer's insightful question regarding the divergent outcomes of annealing *Z*-CDPM under two distinct conditions. While the difference in products (DPXDC in $C_2D_2Cl_4$ vs. DPXC on silica gel) were confirmed through experiments, the detailed mechanism requires further experimental verification. To investigate the underlying cause of this difference, we conducted control experiments using acetic acid to examine potential pH effects, and basic alumina as an alternative solid support to probe the influence of morphology during thermal treatment. According to the data depicted in Supplementary Figs. 32 and 33, the thermal reaction of *Z*-CDPM seems to be related to "morphology changes" rather than pH value. Structurally, the formation of DPXC from DPXDC requires elimination of a volatile molecule, HCN. We propose that the efficient volatilization of HCN in the solid-state environment (e.g., on silica gel) facilitates the elimination reaction and drives the formation of DPXC.

In contrast, in a viscous solution like $C_2D_2Cl_4$, limited HCN diffusion may slow or inhibit this elimination step, allowing DPXDC to accumulate over time. Therefore, the observed divergence likely arises from morphology-dependent HCN removal efficiency, which impacts the balance between cyclization and elimination pathways during the thermal transformation.

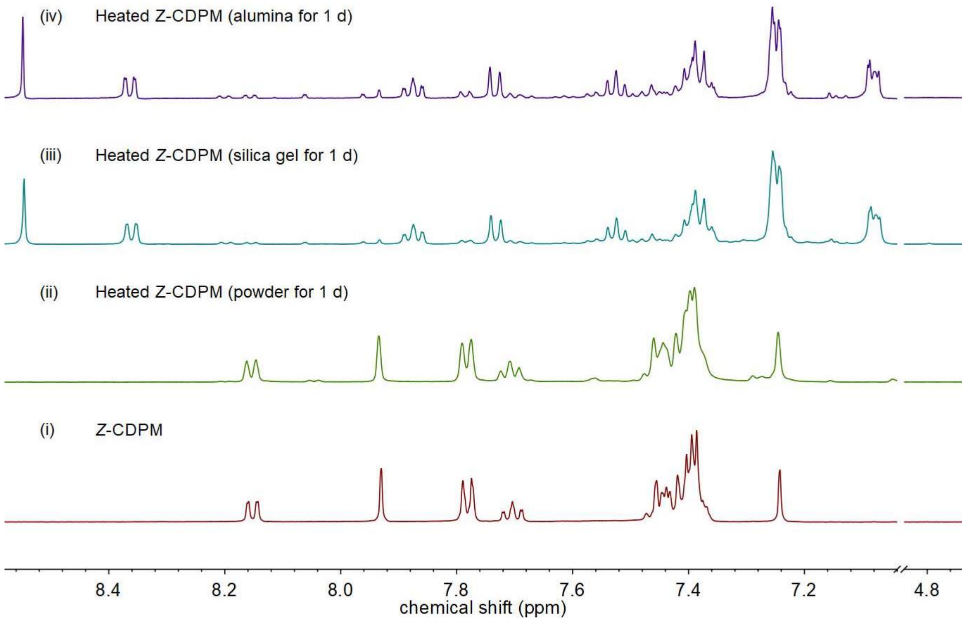

Supplementary Fig. 32 ^{1}H NMR spectra of *Z*-CDPM powder before and after heating at 145 °C in different media for 1 d.

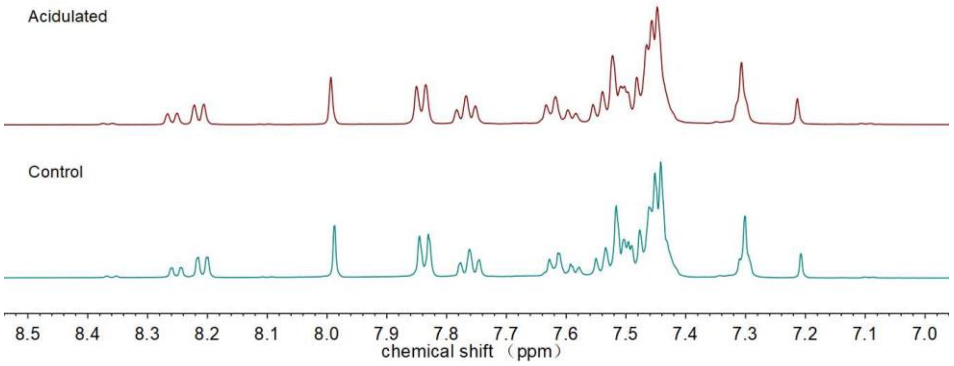

Supplementary Fig. 33. ^{1}H NMR spectra of *Z*-CDPM before and after acetic acid (0.5%, v/v) acidification in $C_2D_2Cl_4$. The sample was heated at 145 °C for 1d.

Response to the Comments of Reviewer 2:

The study presents a novel chromone-based aggregation-induced emission (AIE) luminogen, Z-CDPM, which exhibits five distinct thermally and photochemically induced transformations, leading to six different states. The manuscript aims to establish this system as a promising candidate for stimuli-responsive materials with potential applications in multicolor imaging and information encryption. While the work is scientifically intriguing and provides valuable insights into multiresponsive luminogens, several critical concerns must be addressed before the manuscript can

be considered for publication.

Response: We appreciate the positive assessment from the reviewer. We have revised the manuscript according to your valuable suggestions.

Major Concerns

1. Mechanistic Rigor and Validation

The manuscript proposes that Z-CDPM undergoes five switching processes, including reversible Z/E isomerization, thermal cyclization, thermal elimination, and two photorearrangements. However, the experimental evidence supporting these pathways remains incomplete:

• The Z/E isomerization is described based on NMR studies, but quantitative kinetic data is absent. What is the rate constant for isomerization under different conditions? Are there alternative pathways that could contribute to the observed conversion?

Response: Thanks a lot for these important comments. According to your valuable suggestion, we have evaluated the reaction rate of *Z*/*E* isomerization through dynamic NMR experiment and added the corresponding quantitative kinetic data in Supplementary Fig. 14 in the supporting information. The rate constant for the isomerization of *Z*-CDPM and *E*-CDPM was 0.151 and 0.181 mmol $\cdot L^{-1}\cdot h^{-1}$, respectively. However, although partial interconversion between *Z* and *E* is observed (Figure 2f), the presence of subsequent thermal reactions (e.g., formation of DPXDC) complicates the accurate extraction of the concentration of *Z*/*E*-CDPM.

Considering the photoarrangement pathway shown in Fig. 5e, *Z*-CDPM should also undergo *Z*/*E* isomerization under light irradiation. Thus, the NMR spectra of *Z*-CDPM after UV irradiation for different times was further measured and the results were shown in Supplementary Fig. 47 in the supporting information. Notably, only a weak signal of the specific NMR peaks of *E*-CDPM were detected, illustrating another pathway of *Z*/*E* isomerization and the role of *E*-CDPM as a metastable intermediate in the photoarrangement process. Thus, the *Z*/*E* isomerization of CDPM could be triggered by both annealing and light irradiation, but the rate constant is difficult to determine accurately due to the presence of subsequent thermal/photo reactions.

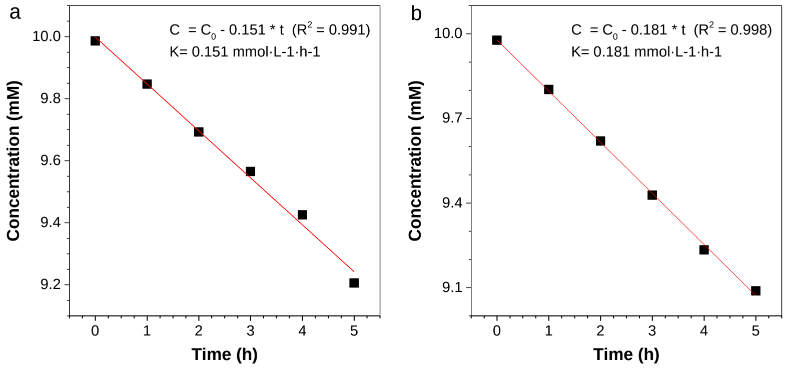

Supplementary Fig. 14 Linear fitting curve of the concentration of *Z*-CDPM (a) and *E*-CDPM (b) towards heating time. The $C_2D_2Cl_4$ solution of *Z*-CDPM (10 mM) and *E*-CDPM (mM) was heat at 145 °C in $C_2D_2Cl_4$.for different time (0, 1, 2, 3, 4, 5 h) and then the concentration was determined by ^{1}H NMR data.

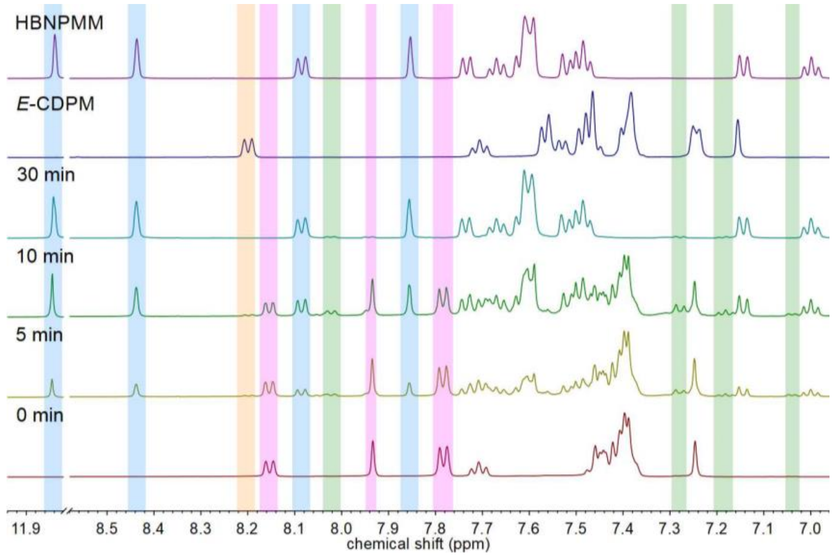

Supplementary Fig. 47 ^{1}H NMR spectra of *Z*-CDPM after UV irradiation in $C_2D_2Cl_4$ for different time (0, 5, 10, 30 min). *Z*-CDPM was highlighted in purple, *E*-CDPM was highlighted in orange, HBNPMM was highlighted in blue, and a possible intermediate(s) was highlighted in green.

• The thermal cyclization process (Cy-Type A) is proposed to proceed via an intermediate, but this intermediate has not been directly characterized. Have the authors considered in situ spectroscopic monitoring (e.g., time-resolved NMR, UV-Vis absorption changes) to track its formation?

Response: We sincerely appreciate the reviewer's insightful suggestion. We acknowledge that the direct monitoring of the proposed intermediate would strengthen our mechanistic proposal. However, the intermediate for the thermal cyclization process (Cy-Type A) of *Z*-CDPM (INT1) is metastable according to its Gibbs free energies of 2.60 Kcal/mol. Also, it would overcome a lower energy barrier of 36.01 Kcal/mol than that of the former *Z*-CDPM (energy barrier of 36.53 Kcal/mol), and subsequently convert to a more stable product of DPXDC (-6.54 Kcal/mol). Theoretically, the concentration of intermediate INT1 would be too low to be monitored. We have conducted the time-resolved NMR experiments and the results is shown in Fig. 3a in the main text, which also shows no obvious signals of any intermediate.

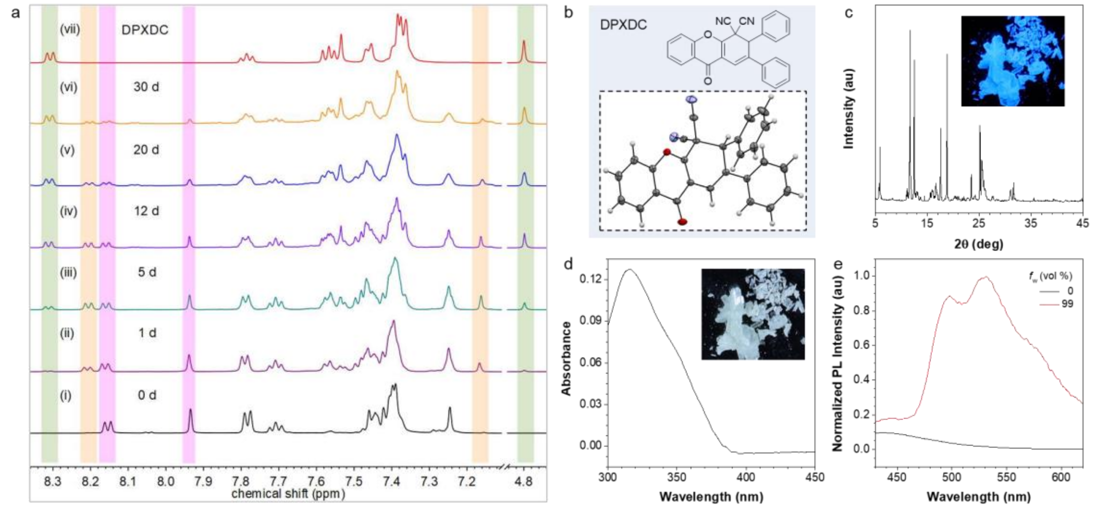

Fig. 3 Thermal cyclization of *Z*-CDPM. (a) ^{1}H NMR spectra of as prepared *Z*-CDPM before and after heating at 145 °C in $C_2D_2Cl_4$ for different times (1, 5, 12, 20, and 30 d). *Z*-CDPM was highlighted in

purple, *E*-CDPM was highlighted in orange, and DPXDC was highlighted in green. (b) Chemical and single-crystal structures of DPXDC. (c) X-ray diffraction (XRD) diffractograms of the crystal powder of DPXDC. Inset: photograph of crystals of DPXDC taken under UV illumination. (d) Absorption spectra of DPXDC (10 μM) in THF. Inset: photograph of crystals of DPXDC taken under day light. (e) PL spectra of DPXDC (10 μM) in THF/water mixtures with different f_w (0 and 99%). λ_{ex} = 365 nm.

• The thermal elimination mechanism suggests that silica gel facilitates the process, potentially by promoting HCN elimination. However, no experimental evidence (e.g., mass spectrometry to confirm volatile byproducts) is provided. Can the authors explicitly demonstrate HCN evolution through gas chromatography or another method?

Response: We appreciate the reviewer's critical assessment and agree that direct experimental validation of HCN evolution would strengthen our mechanistic proposal. According to the difference in molecular structure of DPXDC and DPXC, the generation of volatile HCN during the thermal elimination process is suggested. However, HCN is a reactive substance that are unstable at a high temperature of 145 °C. Even after adding 20 mg of DPXDC for such testing, we ultimately find no signal of HCN in the mass spectrometry. Due to the high toxicity of HCN, further experiments with large amount of DPXDC are not allowed in the lab for safety concerns. To address the reviewer's concerns, we introduce an analogy with dihydroazulene to contextualize the plausibility of HCN release, as both processes involve thermally driven retro-cyclization with elimination of small molecules. Also, we have added the sentence "*According to the difference in molecular structure, a volatile molecule HCN should be generated in the thermal elimination, just as the elimination process of dihydroazulenes*[36]" in the main text for better understanding.

2. Insufficient Comparative Analysis with Existing Systems

The authors claim that Z-CDPM represents an exceptional example of a multiresponsive luminogen. However, the lack of direct comparison with previously reported photochromic and thermochromic systems weakens this claim.

• How does the switching efficiency of Z-CDPM compare to stilbene-based, diarylethene, or spiropyran systems, which have well-characterized reversible photochromic behavior?

Response: Thanks a lot for this insightful comment. We agree that benchmarking against classical photochromic systems such as stilbenes, diarylethenes, and spiropyrans is essential to contextualize the performance of *Z*-CDPM. These classical systems typically exhibit reversible photoisomerization with well-defined switching cycles. However, they generally operate through a single-mode switching mechanism, such as trans-cis isomerization or electrocyclic ring-closing, and often require strict environmental or structural constraints to function efficiently. In contrast, *Z*-CDPM features five distinct, controllable thermal and photochemical switching pathways that lead to six isolable states, which enable information locking rather than merely reversible toggling. This multiresponsive behavior goes beyond the two-state nature of most classical photochromic systems.

In terms of efficiency, we have evaluated the conversion yields of *Z*-CDPM and its derivatives under both thermal and photochemical stimuli based on ^{1}H NMR integration or product isolation. These reactions proceed with high conversion efficiencies, in some cases reaching 80–90%, which are comparable to or higher than those reported for traditional systems. A summary of these data is provided in Supplementary Fig. 59, and several classical systems with detailed conversion efficiency data were also chosen for comparison, as showed in Supplementary Table 6.

To explicitly address the reviewer's suggestion, we have added a discussion in the revised manuscript comparing Z-CDPM with stilbene-, diarylethene-, and spiropyran-type photochromes, highlighting the differences in switching mechanism, reversibility, and functionality. We believe that *Z*-CDPM represents a new class of multistate luminogens that complement classical systems by enabling multi-output logic and high-level data encoding applications.

"*Compared with classic stilbene-based, diarylethene, or spiropyran systems, the high switching efficiency and irreversibility of the photoarrangement and the multiresponsiveness of Z-CDPM would be desirable for functional innovation in photoactivatable systems, which complement classical systems by enabling multi-output logic and high-level data encoding applications.*"

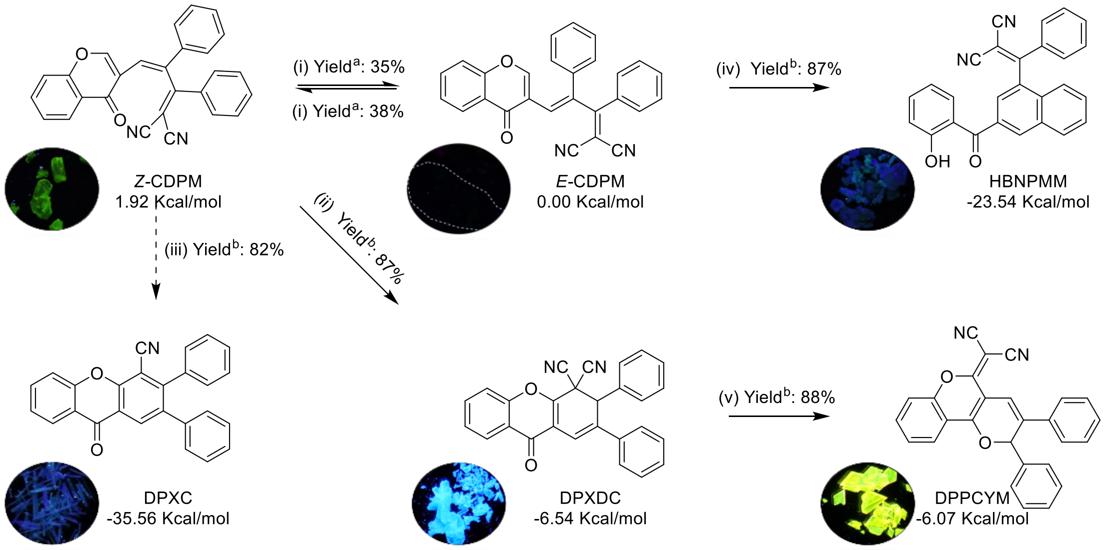

Supplementary Fig. 59 The thermal and photo reactions of *Z*-CDPM under different conditions: (i) annealing at 145 °C in $C_2D_2Cl_4$ for 1 d; (ii) annealing at 200°C in diphenyl ether for 2 h; (iii) annealing at 145 °C in silica gel for 1 d; (iv, v) UV irradiation under a 365 nm UV lamp (0.12 mW/cm^2) at room temperature in $C_2D_2Cl_4$ for 2 h. Insets: photographs of crystals taken under UV irradiation and the relative Gibbs free energies of all six ground-state isomers. [a] Conversion yield determined by ^{1}H NMR data. [b] Conversion yield determined by isolation of products.

Supplementary Table 6. Summary of the photochromic and thermochromic parameters of luminogens reported in this work and other classic systems.

Source	Luminogen name		stimuli	response time	energy barrier (Kcal/mol)	Conversion efficiency (%)
	initial state	transformation				
This work	Z-CDPM	E-CDPM	145 ℃	24 h	27.87	35
	Z-CDPM	DPXDC	200 ℃	2 h	36.54	87
	Z-CDPM	DPXC	145 ℃	6 h	61.01	82
	Z-CDPM	HBNPMM	365 nm	10 s	88.43	87
	DPXDC	DPPCYM	365 nm	10 s	89.70	88
	E-CDPM	Z-CDPM	145 ℃	24 h	29.78	38
Ref. 18	Z-MPPMNAN	E-MPPMNAN	365 nm	96 h	65.64	76
	Z-MPPMNAN	c-MPPMNAN	365 nm	70 h	N.A.	> 90
	Z-MPPMNAN	d-MPPMNAN	365 nm	48 h	69.89	> 90
	E-MPPMNAN	Z-MPPMNAN	75 ℃	36 h	N.A.	> 90
Ref. 32	TPE-SP1-OF	TPE-SP1-CF	254 nm	6 min	N.A.	> 90
	TPE-SP1-CF	TPE-SP1-OF	500 nm	N.A.	N.A.	N.A.
Ref. 35	1a	1b	366 nm	10 s	N.A.	> 90
	1b	1a	450 nm	40 s	N.A.	> 90
Ref. 37	DHAP-H	VHFP-H	365 nm	15 s	81.40	> 90
	VHFP-H	DHAP-H	60 ℃	2 h	N.A.	> 90
Ref. 41	NS	NSI	365 nm	8 h	60.66	55

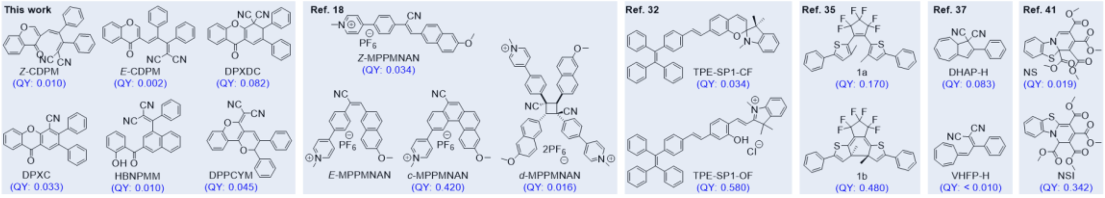

Abbreviation: N.A., not available.

• The study should quantitatively compare its fluorescence quantum yields and switching kinetics with other AIE-based stimuli-responsive luminogens reported in recent literature.

Response: Thanks a lot for your careful review and comments. According to your valuable suggestions, we have measured quantum yields of theses AIEgens and summarized their photophysical properties in Supplementary Fig. 60 in the supporting information. For comparison, several classic AIE-based photo/thermo-responsive luminogens reported in recent studies were chosen, including tetraphenylethylene and cyano-substituted acrylate derivatives, and a summary table contains detailed quantum yields and switching kinetics was given in Supplementary Table S6. The quantum yield of these six compounds was not particularly high as compared with other reported AIEgens. However, our system shows comparable or superior conversion efficiency, along with the unique advantage of offering five switching pathways, which is rarely achieved in existing AIE frameworks. These quantitative comparisons have been incorporated into both the revised Discussion section and Supporting Information, as suggested.

"*It was worth noting that although the quantum yield of these compounds was not particularly high as compared with other reported AIEgens, the high conversion efficience and multiple switchable states would make them ideal candidates for functional systems.*"

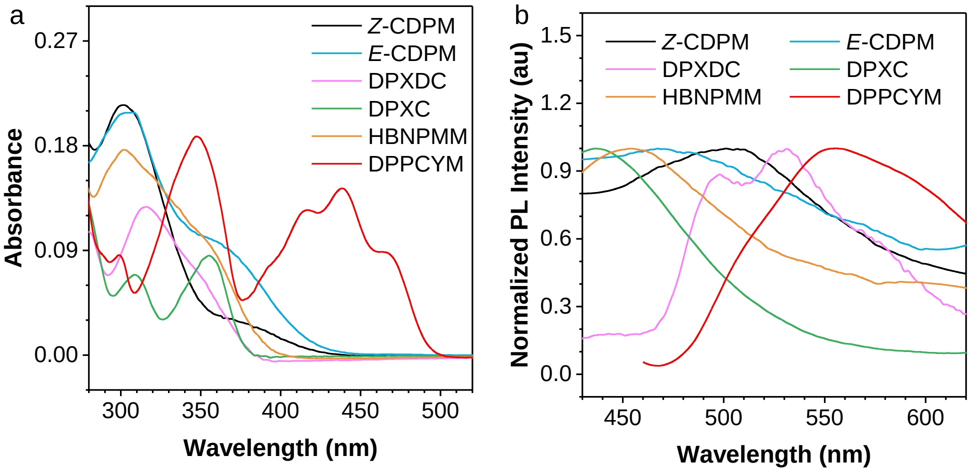

c

Compound	λ_{ab} [nm][a]	ε [$M^{-1}\cdot cm^{-1}$]	λ_{em} [nm][b]	Quantum yield [%]	
				Aggregate[c]	Crystal
Z-CDPM	302 (π→π*)	21497	505	1.0	2.1
E-CDPM	308 (π→π*)	20867	480	0.2	0.5
DPXDC	315 (π→π*)	12730	495	8.2	14.9
DPXC	355 (π→π*)	8560	440	3.3	2.8
HBNPMM	303 (π→π*)	17634	460	1.0	0.9
DPPCYM	440 (π→π*)	14269	550	4.5	37.8

[a] Absorption maximum was measured in THF solution. Concentration= 10 μM. [b] Emission maximum was measured in THF/water mixture (f_w =99%). Concentration= 10 μM. [c] Quantum yield was measured in in THF/water mixture (f_w =99%).

Supplementary Fig. 60 (a) Absorption of AIEgens (10 μM) in THF solution. (b) Normalized PL spectra of AIEgens (10 μM) in THF/water mixtures (f_w = 99%). (c) Summary of photophysical properties of Z-CDPM and its isomers.

Supplementary Table 6. Summary of the photochromic and thermochromic parameters of luminogens reported in this work and other classic systems.

Source	Luminogen name		stimuli	response time	energy barrier (Kcal/mol)	Conversion efficiency (%)
	initial state	transformation				
This work	Z-CDPM	E-CDPM	145 ℃	24 h	27.87	35
	Z-CDPM	DPXDC	200 ℃	2 h	36.54	87
	Z-CDPM	DPXC	145 ℃	6 h	61.01	82
	Z-CDPM	HBNPMM	365 nm	10 s	88.43	87
	DPXDC	DPPCYM	365 nm	10 s	89.70	88
	E-CDPM	Z-CDPM	145 ℃	24 h	29.78	38
Ref. 18	Z-MPPMNAN	E-MPPMNAN	365 nm	96 h	65.64	76
	Z-MPPMNAN	c-MPPMNAN	365 nm	70 h	N.A.	> 90
	Z-MPPMNAN	d-MPPMNAN	365 nm	48 h	69.89	> 90
	E-MPPMNAN	Z-MPPMNAN	75 ℃	36 h	N.A.	> 90
Ref. 32	TPE-SP1-OF	TPE-SP1-CF	254 nm	6 min	N.A.	> 90
	TPE-SP1-CF	TPE-SP1-OF	500 nm	N.A.	N.A.	N.A.
Ref. 35	1a	1b	366 nm	10 s	N.A.	> 90
	1b	1a	450 nm	40 s	N.A.	> 90
Ref. 37	DHAP-H	VHFP-H	365 nm	15 s	81.40	> 90
	VHFP-H	DHAP-H	60 ℃	2 h	N.A.	> 90
Ref. 41	NS	NSI	365 nm	8 h	60.66	55

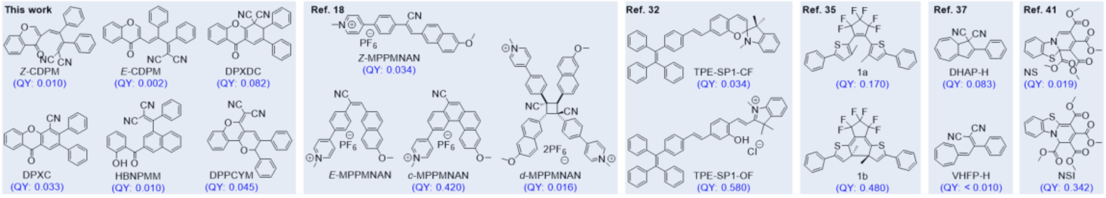

Abbreviation: N.A., not available.

• A table summarizing existing materials, their response times, energy barriers, and photostability would significantly strengthen the manuscript's argument regarding Z-CDPM's advantages.

Response: Thanks a lot for your careful review and comments. We fully agree that a direct comparison between this work and previous reported classic molecules would strengthen the manuscript's argument. Previously, several classic AIE-based photo/thermo-responsive luminogens were chosen for the comparison of quantum yields and switching kinetics, including tetraphenylethylene and cyano-substituted acrylate derivatives. Accordingly, the photochromic and thermochromic parameters of these luminogens were further summarized and added to Supplementary Table 6 in the supporting information for better understanding.

Supplementary Table 6. Summary of the photochromic and thermochromic parameters of luminogens reported in this work and other classic systems.

Source	Luminogen name		stimuli	response time	energy barrier (Kcal/mol)	Conversion efficiency (%)
	initial state	transformation				
This work	Z-CDPM	E-CDPM	145 °C	24 h	27.87	35
	Z-CDPM	DPXDC	200 °C	2 h	36.54	87
	Z-CDPM	DPXC	145 °C	6 h	61.01	82
	Z-CDPM	HBNPMM	365 nm	10 s	88.43	87
	DPXDC	DPPCYM	365 nm	10 s	89.70	88
	E-CDPM	Z-CDPM	145 °C	24 h	29.78	38
Ref. 18	Z-MPPMNAN	E-MPPMNAN	365 nm	96 h	65.64	76
	Z-MPPMNAN	c-MPPMNAN	365 nm	70 h	N.A.	> 90
	Z-MPPMNAN	d-MPPMNAN	365 nm	48 h	69.89	> 90
	E-MPPMNAN	Z-MPPMNAN	75 °C	36 h	N.A.	> 90
Ref. 32	TPE-SP1-OF	TPE-SP1-CF	254 nm	6 min	N.A.	> 90
	TPE-SP1-CF	TPE-SP1-OF	500 nm	N.A.	N.A.	N.A.
Ref. 35	1a	1b	366 nm	10 s	N.A.	> 90
	1b	1a	450 nm	40 s	N.A.	> 90
Ref. 37	DHAP-H	VHFP-H	365 nm	15 s	81.40	> 90
	VHFP-H	DHAP-H	60 °C	2 h	N.A.	> 90
Ref. 41	NS	NSI	365 nm	8 h	60.66	55

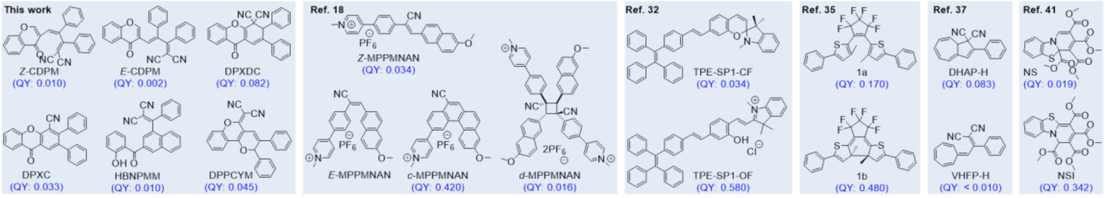

Abbreviation: N.A., not available.

3. Data Presentation and Interpretation

Several aspects of data presentation require improvement to enhance the manuscript's clarity and scientific rigor:

• Figures

• Figure 1b: The reaction conditions (temperature, solvent, light intensity) should be explicitly stated within the figure or its caption.

Response: Thanks a lot for your careful review and comments. According to your valuable suggestion, we have added these reaction conditions in the caption of Figure 1b in the main text.

• Figures 3–6: The photophysical spectra are difficult to interpret due to overlapping signals and unclear baseline corrections. Improved annotation and curve fitting would enhance clarity.

Response: Thanks a lot for your careful review and comments. According to your valuable suggestion, we have clarified the photophysical spectra in Figures 3-6 in the main text.

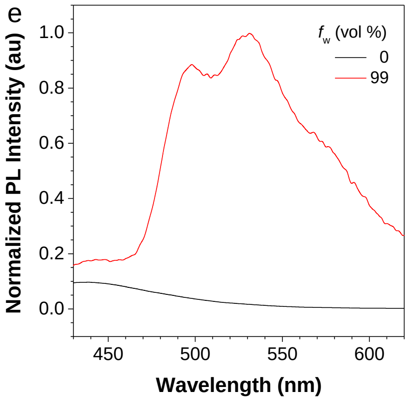

Fig. 3e PL spectra of DPXDC (10 μM) in THF/water mixtures. λ_{ex} = 365 nm.

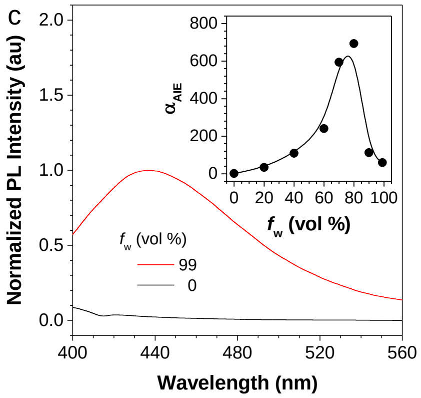

Fig. 4c PL spectra of *Z*-DPXC in THF/water mixtures (10 μM). Inset: plots of I/I_0 value versus the f_w of THF/water mixtures of DPXC, where I_0 = PL intensity in pure THF solution. $\lambda_{ex/em}$ = 355/460 nm.

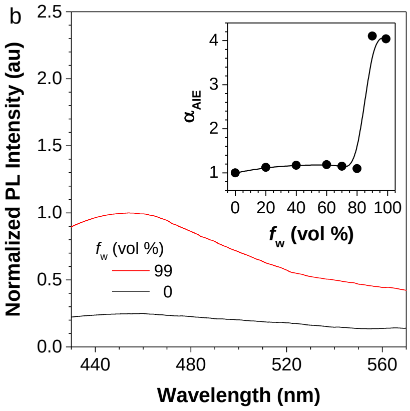

Fig. 5b PL spectra of HBNPMM (10 μM) in THF/water mixtures. Inset: plots of I/I_0 value versus f_w of THF/water mixtures of DPXC, where I_0 = PL intensity in pure THF solution. $\lambda_{ex/em}$ = 355/460 nm.

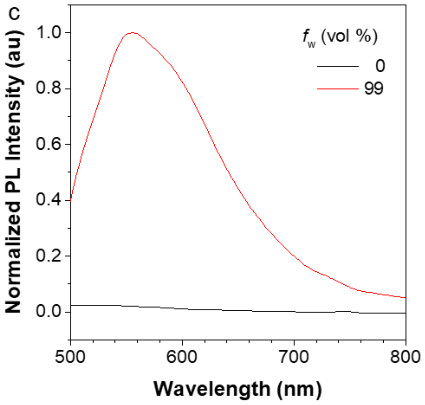

Fig. 6c PL spectra of HBNPMM (10 μM) in THF/water mixtures with different water fractions (f_w). λ_{ex} = 440 nm.

• Figure 7 (QR code applications): While visually appealing, the lack of quantitative discussion on long-term stability and reproducibility under real-world conditions is a major limitation.

Response: Thanks a lot for your careful review and comments. We have evaluated the long-term stability of the manufactured QR code and added the results in Supplementary Fig. 64 in the supporting

information according to your valuable suggestion. Since the QR code could be detected even after 7 d of standing, its long-term stability is illustrated. In addition, due to the irreversibility of the thermal elimination and photoarrangement reaction, the AIE plate cannot be reused. To better convey this point, we have added the following discussion on this issue in our revised manuscript.

"Because of the high conversion efficiency and irreversibility of both the thermal and photo switching process, DPXDC could conduct one-way discoloration in information encryption systems with long-term stability (Supplementary Fig. 45)."

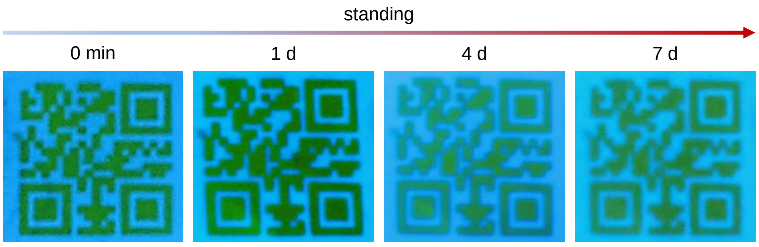

Supplementary Fig. 64 Photographs of the developed colored "QR code" of DPXDC after standing at room temperature for different times (0, 1, 4, 7 d).

• Spectroscopic Data

• The NMR spectra provided for structural characterization are not sufficiently detailed. Enlarged views of key chemical shifts and integration values should be included to facilitate independent verification.

Response: Thanks a lot for the important comment. We have added enlarged views of key chemical shifts and integration values of the NMR spectra in the supporting information according to your valuable suggestion.

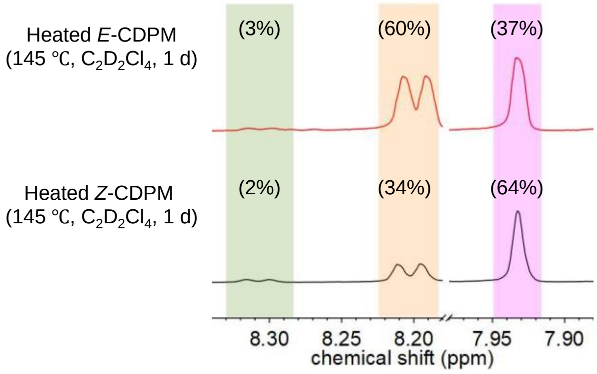

Supplementary Fig. 13 Indicative sections of ^{1}H NMR spectra of *Z*-CDPM and *E*-CDPM after heating at 145 °C in $C_2D_2Cl_4$ for 1 d. *Z*-CDPM was highlighted in purple, *E*-CDPM was highlighted in orange, and DPXDC was highlighted in green.

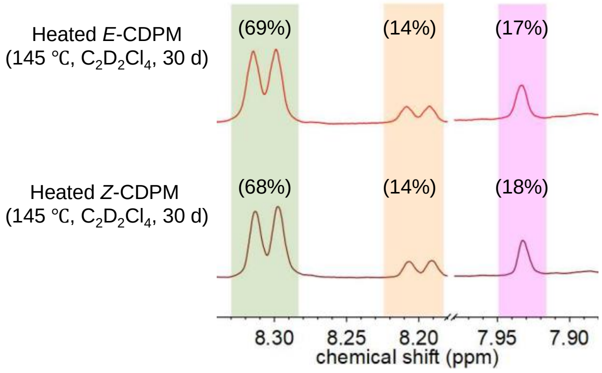

Supplementary Fig. 21 Indicative sections of ^{1}H NMR spectra of *Z*-CDPM and *E*-CDPM after heating at 145 °C in $C_2D_2Cl_4$ for 30 d. *Z*-CDPM was highlighted in purple, *E*-CDPM was highlighted in orange, and DPXDC was highlighted in green.

• The UV-Vis and PL spectra lack a clear discussion of peak assignments. What electronic transitions correspond to the observed spectral shifts?

Response: Thanks a lot for your careful review and comments. According to your valuable suggestions, we have summarized the photophysical properties of these AIEgens and added Supplementary Fig. 45 in the supporting information, which contains peak assignments and the corresponding electronic transitions. We are extremely grateful for the detailed and insightful suggestions of the reviewer.

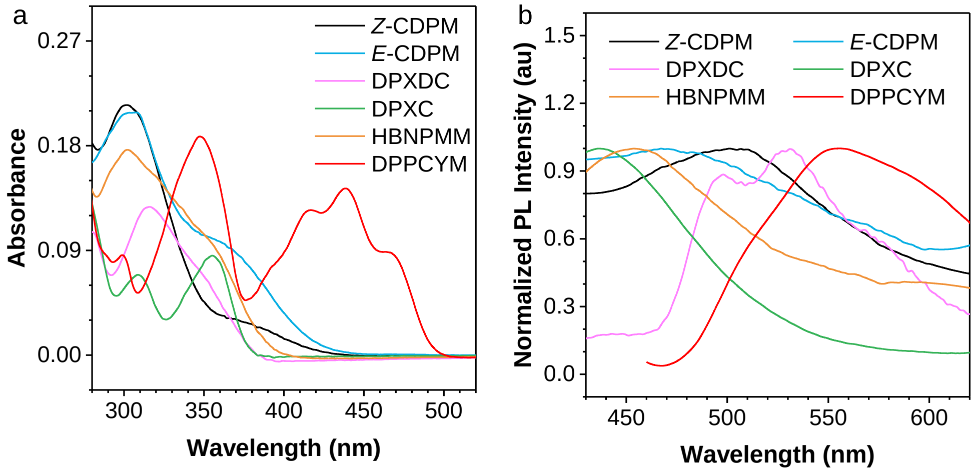

c

Compound	λ_{ab} [nm][a]	ε [$M^{-1}\cdot cm^{-1}$]	λ_{em} [nm][b]	Quantum yield [%]	
				Aggregate[c]	Crystal
Z-CDPM	302 (π→π*)	21497	505	1.0	2.1
E-CDPM	308 (π→π*)	20867	480	0.2	0.5
DPXDC	315 (π→π*)	12730	495	8.2	14.9
DPXC	355 (π→π*)	8560	440	3.3	2.8
HBNPMM	303 (π→π*)	17634	460	1.0	0.9
DPPCYM	440 (π→π*)	14269	550	4.5	37.8

[a] Absorption maximum was measured in THF solution. Concentration= 10 μM. [b] Emission maximum was measured in THF/water mixture (f_w =99%). Concentration= 10 μM. [c] Quantum yield was measured in in THF/water mixture (f_w =99%).

Supplementary Fig. 60 (a) Absorption of AIEgens (10 μM) in THF solution. (b) Normalized PL spectra of AIEgens (10 μM) in THF/water mixtures (f_w = 99%). (c) Summary of photophysical properties of Z-CDPM and its isomers.

• Computational Analysis

• The DFT calculations provide valuable energy profiles but lack solvent effects, which may be significant for photochemical and thermal processes. Have the authors considered solvent-corrected energy calculations?

Response: We appreciate the reviewer's insightful suggestion. Indeed, solvent effects can significantly influence reaction energetics, particularly in photochemical and thermal transformations. In our original study, the thermal elimination of *Z*-CDPM was carried out in the solid state on silica gel (Fig. 4e), where the solvent environment plays a minimal role. Therefore, solvent corrections were not applied for this process, and gas-phase DFT calculations were used to extract qualitative mechanistic trends and relative energy barriers. However, based on further experimental observations, we recognize that most steps leading to the formation of DPXDC—including *Z*/*E* isomerization, 6 π -electron cyclization, and the [1,5]-H shift—can also proceed in solution. Therefore, solvent effects are indeed relevant for this part of the transformation. Only the final step—from DPXDC to DPXC—requires silica gel for effective HCN

elimination and is best modeled under solid-state conditions.

To address this concern, we have now performed additional single-point energy calculations using the SMD implicit solvation model (solvent: $C_2H_4Cl_2$) at the M06-2X/6-31G(d,p) level. The solvent-corrected energy profiles show consistent trends with the original gas-phase results, thereby reinforcing the robustness of the proposed mechanisms.

As a result, we have updated Fig. 4e, Fig. 5e, and Fig. 6e in the main text to reflect the solvent-corrected energy profiles. The original gas-phase energy profiles have been retained in the Supporting Information as Supplementary Figs. 35, 49, and 58 for reference and direct comparison. For the reviewer's convenience, both the original and solvent-corrected energy profiles are provided below for direct comparison.

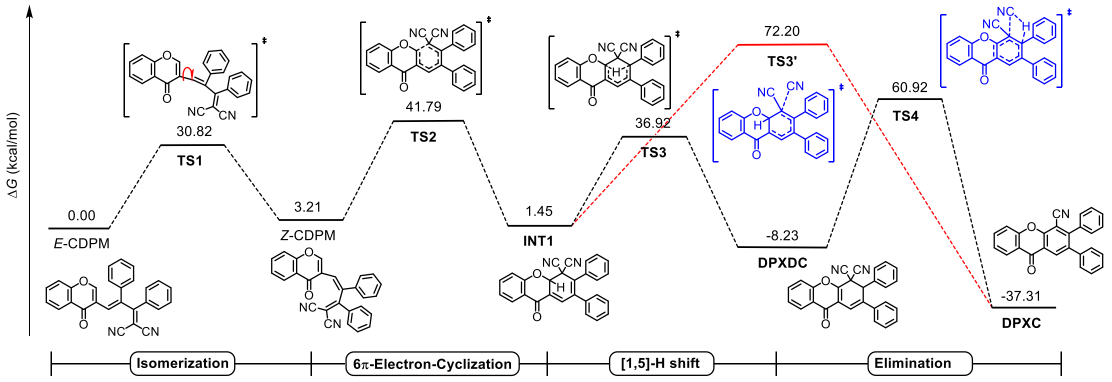

Supplementary Fig. 35 Energy profile for the thermal reactions of *Z*-CDPM calculated at the M06-2X/6-31G(d,p) level without solvent correction.

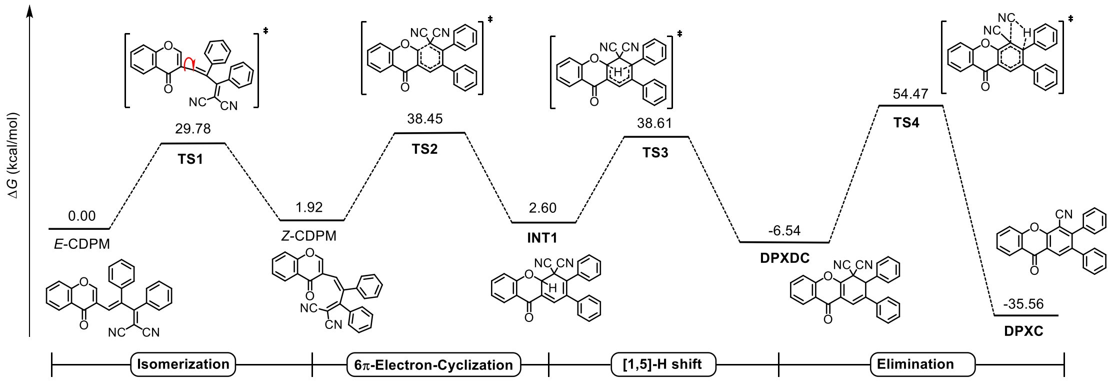

Fig. 4e Energy profile for the thermal reactions of *Z*-CDPM calculated at the M06-2X/6-31G(d,p) level with solvent correction.

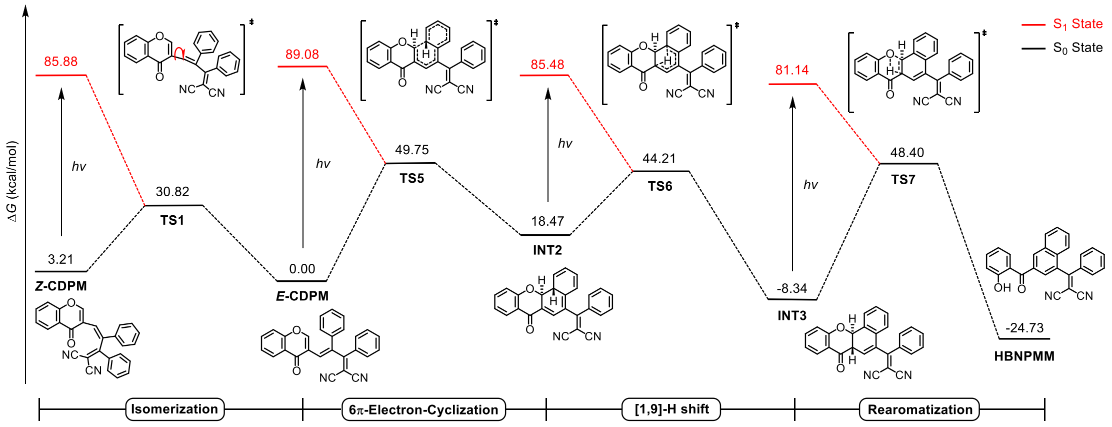

Supplementary Fig. 49 Energy profile for the photoarrangement of *Z*-CDPM calculated at the M06-2X/6-31G(d,p) level without solvent correction.

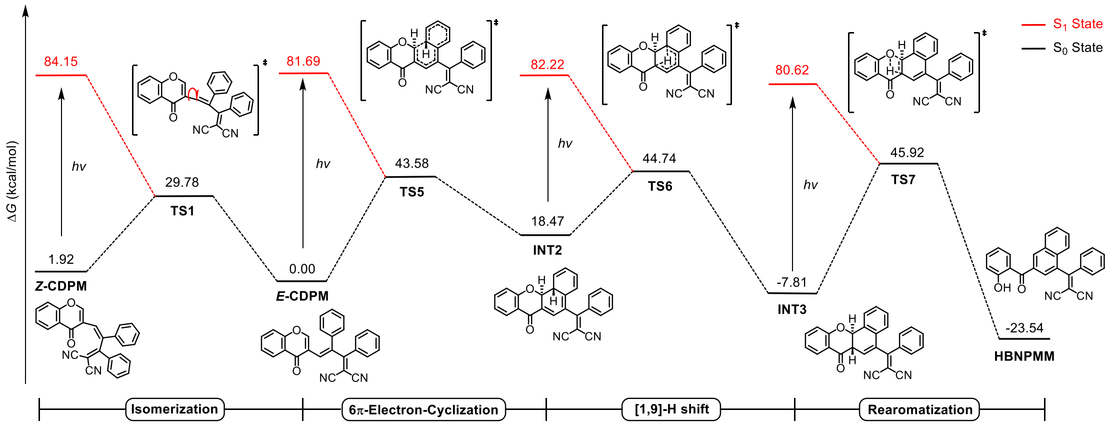

Fig. 5e Energy profile for the photoarrangement of *Z*-CDPM calculated at the M06-2X/6-31G(d,p) level with solvent correction.

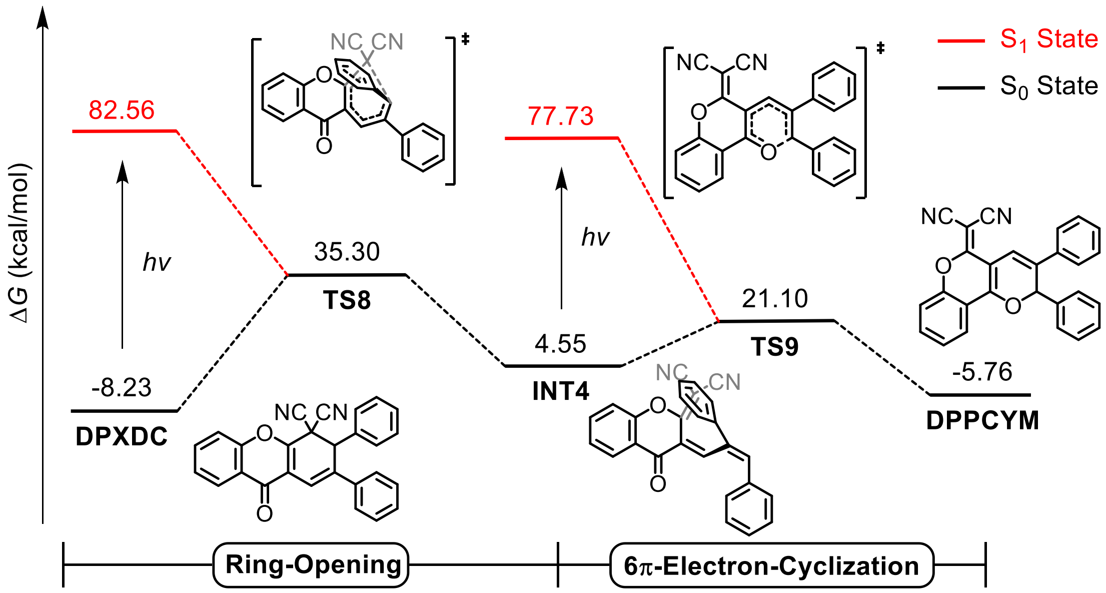

Supplementary Fig. 58 Energy profile for the photoarrangement of DPXDC calculated at the M06-2X/6-31G(d,p) level without solvent correction.

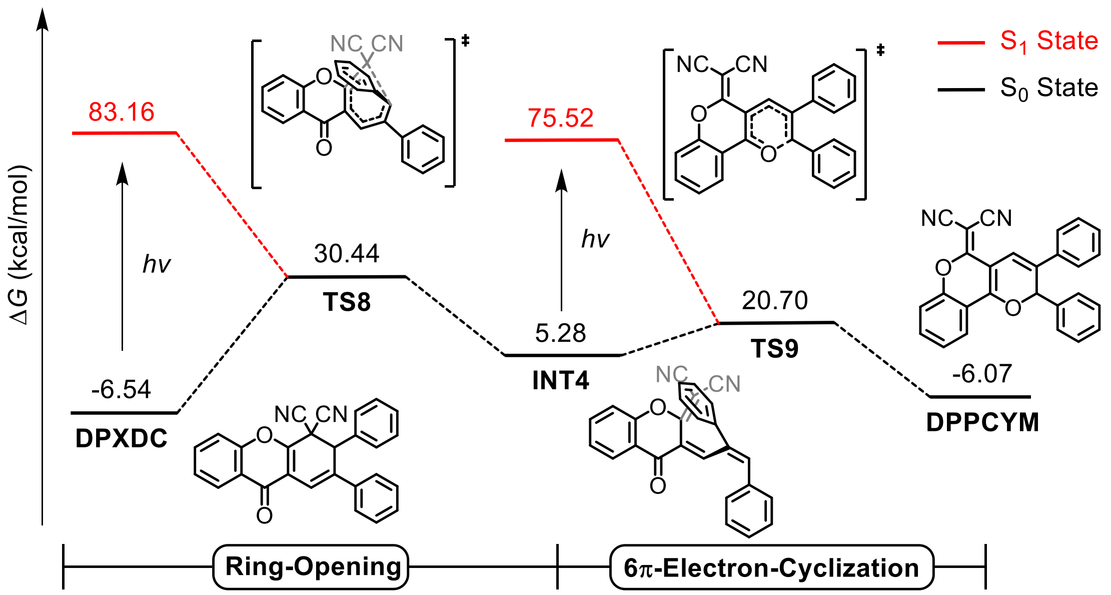

Fig. S6e Energy profile for the photoarrangement of DPXDC calculated at the M06-2X/6-31G(d,p) level with solvent correction.

• The absence of excited-state dynamics simulations raises concerns about the accuracy of the proposed photochemical mechanisms.

Response: Thank you for this important comment. We fully agree that excited-state dynamics simulations—such as surface hopping or nonadiabatic molecular dynamics (NAMD)—can offer valuable insights into photochemical mechanisms by capturing ultrafast transitions and nonadiabatic effects. However, the *Z*-CDPM system investigated here features multiple conjugated aromatic units, resulting in a dense manifold of excited states and a relatively large molecular size. These factors render full excited-state trajectory simulations computationally prohibitive using currently available resources.

Furthermore, this system exhibits complex multi-state switching behavior, including irreversible photoarrangements and solid-state transformations, which further complicate excited-state modeling.

It is also important to emphasize the disparity in timescales: even the fastest photoreaction in our system—the photoarrangement of *Z*-CDPM—requires on the order of 2 hours to reach completion under experimental conditions. This is several orders of magnitude longer than the femtosecond-to-picosecond timescales accessible to excited-state molecular dynamics simulations, which are typically suited for ultrafast internal conversions or intersystem crossings. As such, direct simulation of the full photochemical transformations observed in this work is not only infeasible but also likely to miss the key long-timescale processes governing the observed reactivity.

Given these constraints, we have instead used a combination of TD-DFT vertical excitation analysis and energy profile to rationalize the photochemical pathways. These static calculations already reveal energetically accessible transitions and support the feasibility of the experimentally observed photoinduced structural changes. To clarify this limitation and approach, we have added a statement in the revised manuscript (Discussion section):

"*While direct excited-state dynamics simulations were not performed due to system size and computational cost, the mechanistic plausibility was assessed via TD-DFT and potential energy surface analysis, which captured the key trends and enabled qualitative rationalization of the photoresponses.*"

Minor Concerns

• Language and Clarity

• Several sections, particularly in the Introduction and Results, contain redundant phrasing and overly long sentences. Improving conciseness would enhance readability.

Response: Thanks a lot for your careful review and comments. We fully agree that redundant phrasing and overly long sentences would weaken readability of this paper. According to your valuable suggestion, we have carefully checked redundant phrasing and overly long sentences in the introduction and results, and concise them in the main text.

• The term “quintuple switching” is not commonly used in the field. A clearer phrase, such as “five distinct and controllable transformations,” may be more appropriate.

Response: Thanks a lot for your careful review and comments. We have replaced the term “quintuple switching” by “five distinct and controllable transformations”in the main text according to your valuable suggestion.

• References

• The manuscript extensively cites recent work in AIE materials, which is commendable. However, some key historical references on photochromism and thermal isomerization (e.g., seminal work on spiropyrans and diarylethenes) are missing and should be incorporated.

Response: Thanks a lot for your careful review and comments. We have added numerous key historical references on photochromism and thermal isomerization in the main text according to your valuable suggestion.

Based on the concerns outlined above, I recommend a Major Revision before the manuscript can be considered for publication.

Response to the Comments of Reviewer 3:

In this work He et al. describe a luminescent molecular system consisting of six different states derived from a single molecular framework. Upon different heat (temperature) and light stimuli the six different states are selectively accessible when using suitable combinations of the stimuli. One heating conversion is reversible to some degree. All six states show different luminescence and also AIE behavior both in solution and on solid support, which allows the authors to use them in multi-level stimuli responsive applications, for example in encryption, optical memories and information inscription/erasure.

The structural identity of the six different isomers is convincingly elucidated by single-crystal X-ray diffraction and the individual conversions between isomers seem to be of very high conversion as well in each case. The authors also deliver a quantum chemical description of the states as well as conversion barriers and thus gained first insights into the isomerization mechanisms.

Overall I believe that this work certainly has the potential to be publishable in a high-impact journal such as Nature Communcations. The molecular system reported is novel and unique and shows very good conversion between the states and correlated significant changes of luminosity. The authors make full use of this complex behavior in a number of elevated photonic applications, so in general novelty and performance really seem to be excellent.

However, there are a number of issues that need to be addressed by the authors in order to accurately describe their system and elucidate the performance properties. With respect to theory, there are also a number of points that need addressing.

Response: We appreciate the positive assessment from the reviewer. We have revised the manuscript according to your valuable suggestions.

Major points:

- First, not all transformations are clearly quantified. Please provide yields for every single isomer transformation, such that these six conversions can actually be compared correctly and also report how these yields were determined (NMR, isolation of products and balancing…). From the NMR traces shown, it seems that these conversions are in fact very high and also the authors say that they prepare individual isomers synthetically using the heat/irradiation steps.

Response: Thanks a lot for your careful review and comments. According to your valuable suggestions, we have summarized the yields for every isomer transformation and added the corresponding data in Supplementary Fig. 59 in the supporting information. Specifically, the conversion yield of *Z*/*E* isomerization determined by ^{1}H NMR data, while the conversion yields of other reactions are determined by isolation of products. Also, the results revealed the high conversion efficiency of the thermal cyclization, thermal elimination and phtoarrangement reactions.

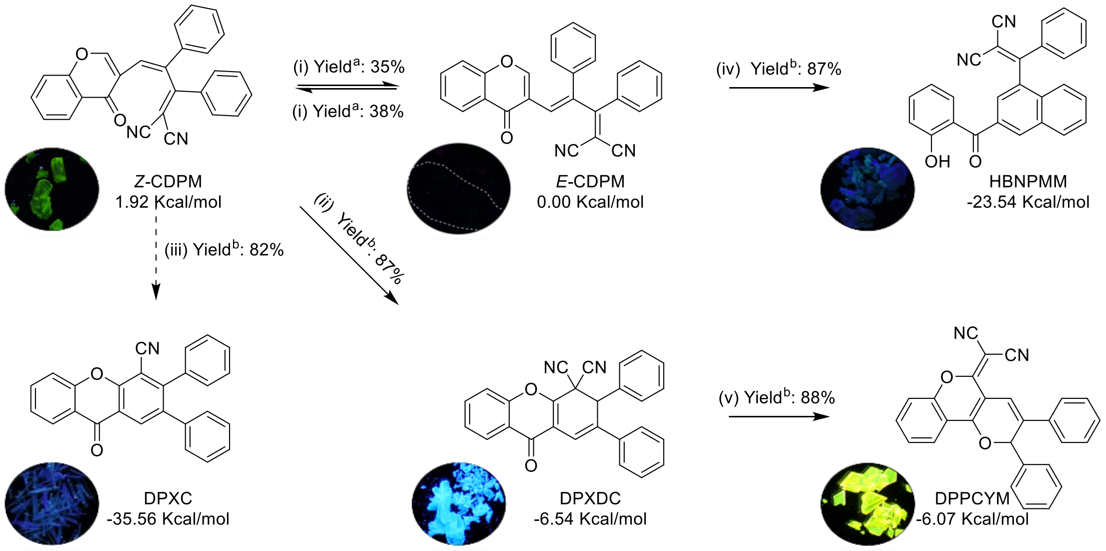

Supplementary Fig. 59 The thermal and photo reactions of *Z*-CDPM under different conditions: (i) annealing at 145 °C in $C_2D_2Cl_4$ for 1 d; (ii) annealing at 200°C in diphenyl ether for 2 h; (iii) annealing at 145 °C in silica gel for 1 d; (iv, v) UV irradiation under a 365 nm UV lamp (0.12 mW/cm^2) at room temperature in $C_2D_2Cl_4$ for 2 h. Insets: photographs of crystals taken under UV irradiation and the relative Gibbs free energies of all six ground-state isomers. [a] Conversion yield determined by ^{1}H NMR data. [b] Conversion yield determined by isolation of products.

- In addition to that, I miss an overview Figure, which shows a) photographs of all solid materials and solutions to be able to show the oftentimes cited "coloration" or "decoloration" in the manuscript text. The same was already done for fluorescence in Figure 7a. Along with it, molar absorptions (extinction coefficients) for all six isomers need to be shown side by side in one absorption diagram as well as the corresponding fluorescence diagrams taken at the same

concentrations of samples.

Response: Thanks a lot for your careful review and comments. We fully agree that a comprehensive overview figure is necessary to visually consolidate the coloration/decoloration phenomena and spectroscopic data for all six isomers. According to your valuable suggestion, we have summarized the photophysical properties of these AIEgens and added the corresponding overview Figures in the supporting information. The fluorescent images of all solid materials are shown in Supplementary Fig. 59. Also, their absorption and PL spectrum measured at a concentration of 10 μM, along with the corresponding extinction coefficients are depicted in Supplementary Fig. 60.

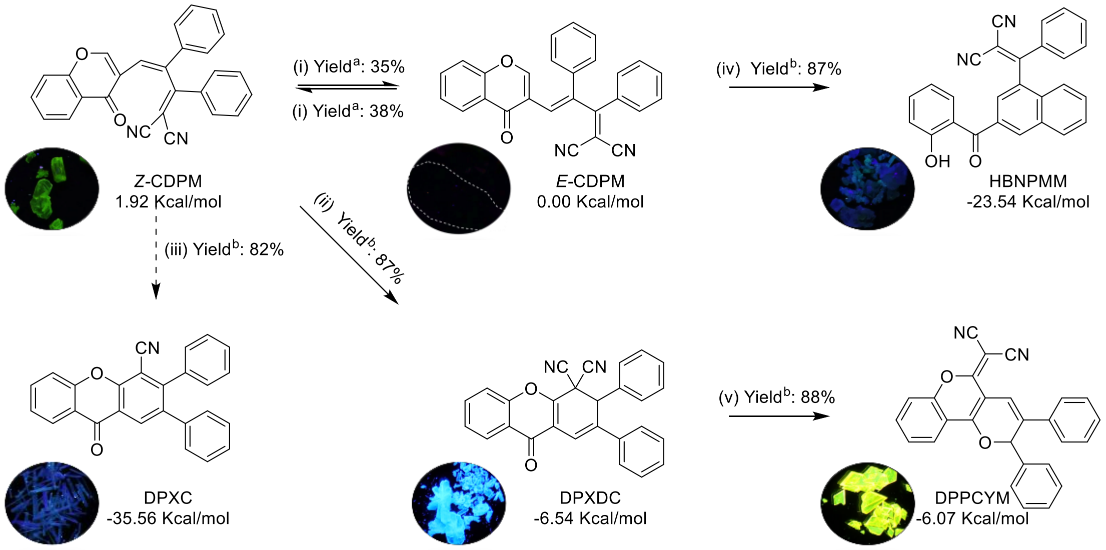

Supplementary Fig. 59 The thermal and photo reactions of *Z*-CDPM under different conditions: (i) annealing at 145 °C in $C_2D_2Cl_4$ for 1 d; (ii) annealing at 200°C in diphenyl ether for 2 h; (iii) annealing at 145 °C in silica gel for 1 d; (iv, v) UV irradiation under a 365 nm UV lamp (0.12 mW/cm^2) at room temperature in $C_2D_2Cl_4$ for 2 h. Insets: photographs of crystals taken under UV irradiation and the relative Gibbs free energies of all six ground-state isomers. [a] Conversion yield determined by ^{1}H NMR data. [b] Conversion yield determined by isolation of products.

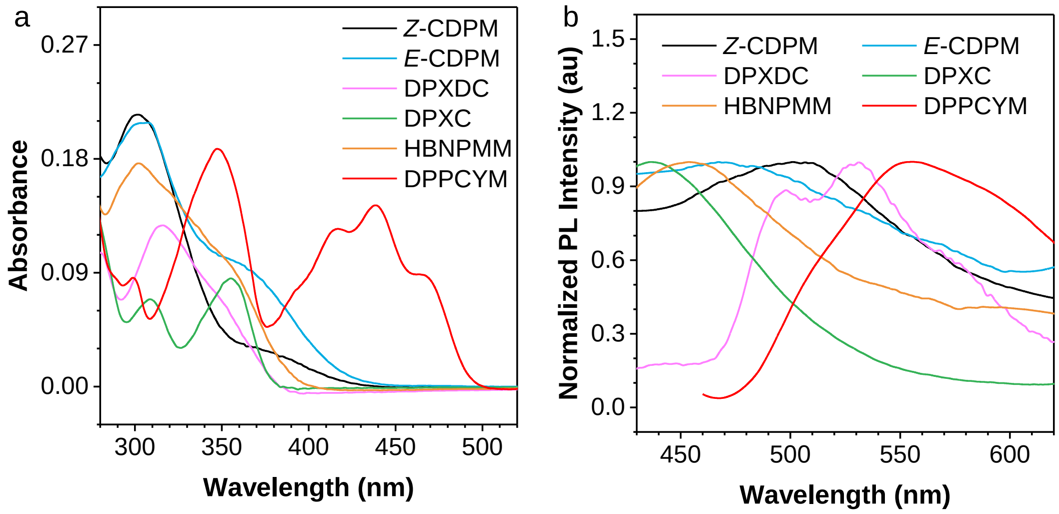

c

Compound	λ_{ab} [nm][a]	ε [M^{-1}·cm^{-1}]	λ_{em} [nm][b]	Quantum yield [%]	
				Aggregate[c]	Crystal
Z-CDPM	302	21497	505	1.0	2.1
E-CDPM	308	20867	480	0.2	0.5
DPXDC	315	12730	495	8.2	14.9
DPXC	355	8560	440	3.3	2.8
HBNPMM	303	17634	460	1.0	0.9
DPPCYM	440	14269	550	4.5	37.8

[a] Absorption maximum was measured in THF solution. Concentration= 10 μM. [b] Emission maximum was measured in THF/water mixture (f_w =99%). Concentration= 10 μM. [c] Quantum yield was measured in in THF/water mixture (f_w =99%).

Supplementary Fig. 60 (a) Absorption of AIEgens (10 μM) in THF solution. (b) Normalized PL spectra of AIEgens (10 μM) in THF/water mixtures (f_w = 99%). (c) Summary of photophysical properties of Z-CDPM and its isomers.

- Second, there is an issue with the analysis of the thermal reactions and the interpretations thereof. When heating the Z/E CDPM isomers they do interconvert into each other at 145 °C but also further conversion to the DPXDC isomer starts to occur. This is seen in Fig. 3a for example. Kinetic modeling is needed here to distinguish different possible scenarios but also to deliver actual experimental Gibbs energies of activations for these processes to compare with the quantum chemical modelling. Two scenarios are possible at least: it could be 1. that ZCDPM is going to ECDPM first and then ECDPM is going into DPXDC. Or 2. ZCDPM is branching into ECDPM and DPXDC to some degree. However clearly ECDPM is going into DPXDC!

Response: Thanks a lot for your careful review and comments. We fully agree that the thermal behavior of *Z*/*E*-CDPM isomers involves multiple competing pathways, and that a more rigorous kinetic treatment

would help distinguish between sequential and branching reaction mechanisms. In our study, the thermal conversion experiments (e.g., Fig. 2f and Fig. 3a) indeed show simultaneous $Z \leftrightarrow E$ isomerization and gradual formation of DPXDC over time. As correctly pointed out, at least two mechanistic scenarios are plausible:

(1) Z-CDPM isomerizes to E-CDPM, which then thermally converts to DPXDC (sequential mechanism), or

(2) Z-CDPM can undergo parallel pathways—direct conversion to DPXDC or isomerization to *E*-CDPM (branching mechanism).

Our time-course NMR experiments (Supplementary Figs. 13–14, 18, 20–21, 27, 31) reveal that *E*-CDPM gradually decreases with concurrent DPXDC formation, supporting that *E*-CDPM → DPXDC is indeed operative and likely a dominant pathway under thermal conditions. However, *Z*-CDPM can also convert to DPXDC under certain conditions (e.g., prolonged heating, Supplementary Fig. 18), indicating that the *Z*-CDPM → DPXDC pathway is also accessible, and may occur independently of prior isomerization to *E*-CDPM.

To further support this mechanistic picture, we have included a revised energy profile (see revised Fig. 4e and Supplementary Fig. S35), which illustrates both the isomerization and cyclization pathways. As shown in the updated figure (Supplementary Fig. S35), the energy barrier for $Z \rightarrow E$ isomerization (TS1) is relatively low (29.78 kcal/mol), and both *Z*- and *E*-CDPM converge onto a common reaction coordinate leading to the cyclized intermediate INT1 via TS2 (38.45 kcal/mol). From INT1, a [1,5]-hydride shift leads to DPXDC via TS3 (38.61 kcal/mol).

While the current data suggest that isomerization and cyclization are both thermally accessible and may occur concurrently, precise resolution of the pathway branching and rate-limiting steps would indeed benefit from kinetic modeling and temperature-dependent expriments. Such studies would allow experimental estimation of activation parameters (e.g., $\Delta G^{\ddagger}$) for comparison with our DFT predictions. We attempted to experimentally determine the activation barrier for $Z \leftrightarrow E$ isomerization, but no meaningful kinetic data could be extracted. This limitation likely arises from the fact that the energy barrier (~30 kcal/mol) lies near the edge of reliable detection under our current setup, where slow isomerization overlaps with concurrent downstream reactions, such as cyclization or elimination. Under such conditions, rate deconvolution becomes nontrivial, and Arrhenius- or Eyring-based analysis is compromised by overlapping signals and low time resolution. Therefore, obtaining reliable experimental Gibbs activation energies for direct comparison with theoretical models is not currently feasible. Nevertheless, a more comprehensive kinetic investigation remains a valuable direction for future work.

To clarify the stereochemical connectivity of the cyclization pathway, we also performed an intrinsic reaction coordinate (IRC) analysis on TS2. The resulting structure, TS2-IRC-Reactant, lies in a shallow conformational region and can interconvert with both *Z*-CDPM and *E*-CDPM via single-bond rotation, as shown in Supplementary Fig. 38. This result indicates that the cyclization process from either isomer proceeds through a shared conformational space, and the corresponding transition state is not stereochemically restricted. This conformational flexibility explains why both *Z*-CDPM → INT1 and *E*-CDPM → INT1 pathways are thermally accessible, consistent with both our experimental observations and computed energy landscape.

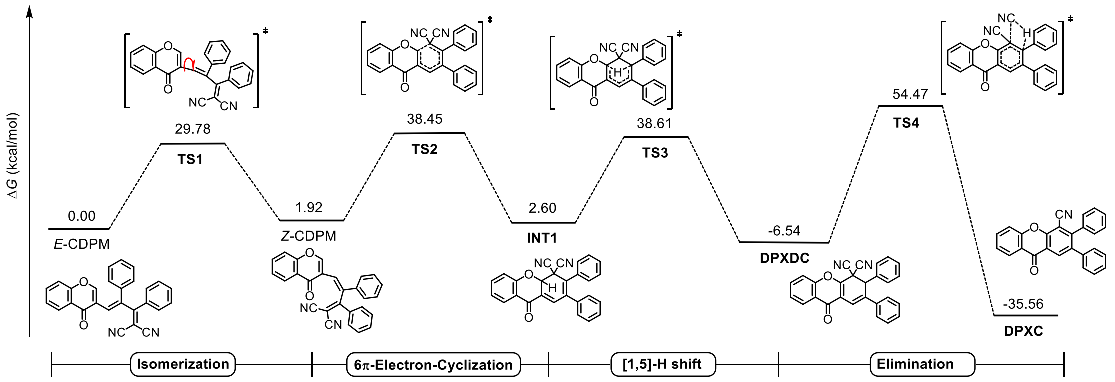

Fig. 4e Energy profile for the thermal reactions of *Z*-CDPM calculated at the M06-2X/6-31G(d,p) level with solvent correction.

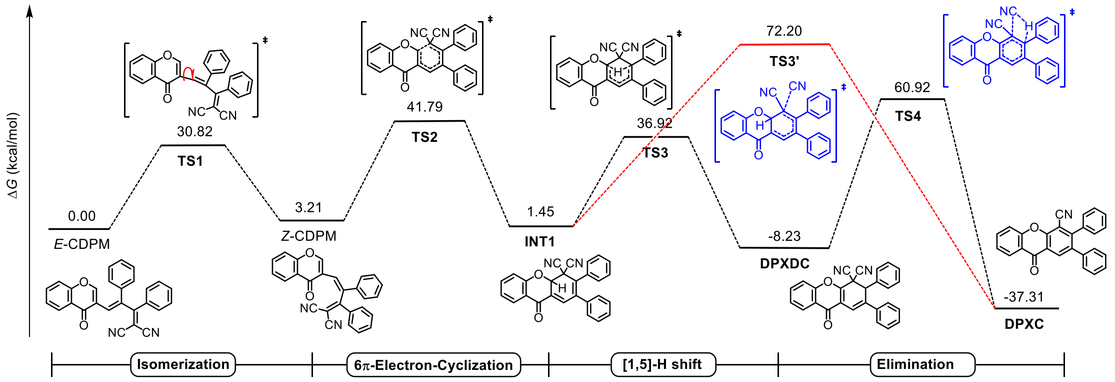

Supplementary Fig. 35 Energy profile for the thermal reactions of *Z*-CDPM calculated at the M06-2X/6-31G(d,p) level without solvent correction.

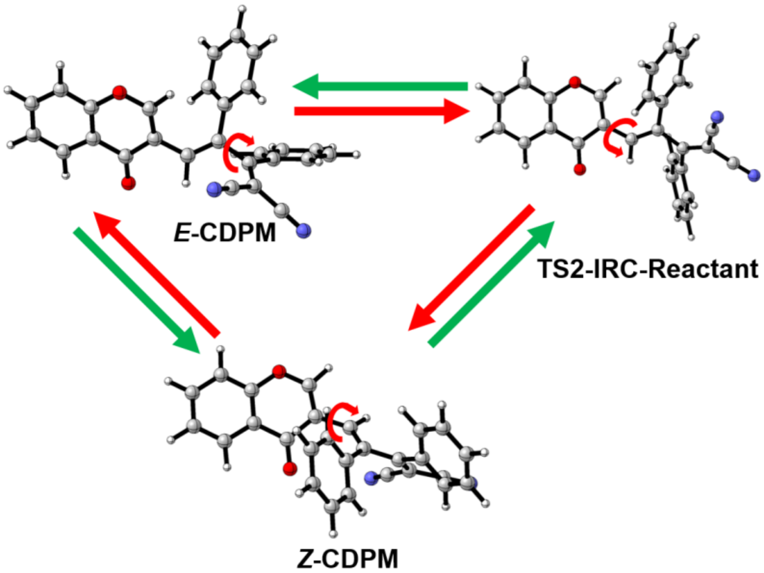

Supplementary Fig. 38 Comparison of the optimized structures of *E*-CDPM and *Z*-CDPM with the reactant-side structure obtained from the IRC pathway of TS2. The TS2-IRC-Reactant can interconvert with both *E*-CDPM and *Z*-CDPM via single-bond rotations, indicating that these species share a flexible conformational landscape.

To reflect this in the manuscript, we have added the following statement in the revised Discussion section:

"*While our data suggest that E-CDPM is a key intermediate in route to DPXDC, the thermal conversion of Z-CDPM may proceed via both sequential and branching pathways.*"

- A related issue is the apparent equilibration between Z and E CDPM. It looks like they do reach almost 1:1 ratio before significant further thermal reactions occur. This is important as it shows that the DFT calculation giving 3.4 kcal/mol energy difference is not correct. The authors say that this is very low energy difference, but at such a value the ratio between E and Z should be around 98%:2% at 145 °C, which is not observed experimentally. This hints already at some problems in the theory.

Response: Thanks a lot for your careful review and comments. We agree that the experimentally observed near 1:1 equilibrium ratio between *Z*- and *E*-CDPM under heating conditions does not align with the DFT-predicted free energy difference. In fact, our calculations yield a ΔG = 3.21 kcal·mol^{-1} (rather than 3.4 kcal·mol^{-1}), which would still theoretically favor one isomer overwhelmingly (97.94% vs. 2.06%, based on the Boltzmann distribution) at 145 °C under ideal equilibrium conditions.

This discrepancy likely reflects the limitations of static DFT calculations in capturing the full thermodynamic landscape of real systems. Several factors may contribute:

1. DFT Method Error Range:

DFT inherently involves approximations, and the error in computed free energy differences can be up to ±2–4 kcal·mol^{-1} depending on the functional and the system. The 3.21 kcal·mol^{-1} value lies within this uncertainty range and may not reliably reflect small thermodynamic preferences. As shown below, after change the DFT functional from M062X to TPSS, the energy difference change from 3.21 kcal·mol^{-1} to 3.04 kcal·mol^{-1}.

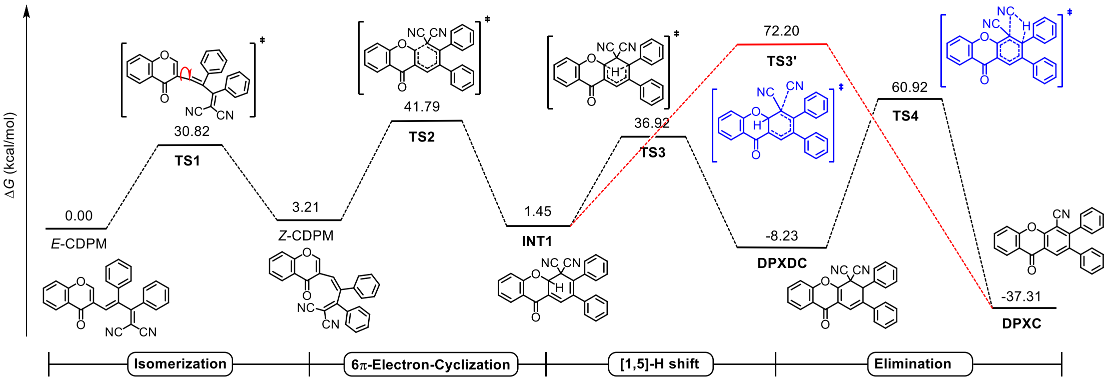

Supplementary Fig. 35 Energy profile for the thermal reactions of *Z*-CDPM calculated at the M06-2X/6-31G(d,p) level without solvent correction.

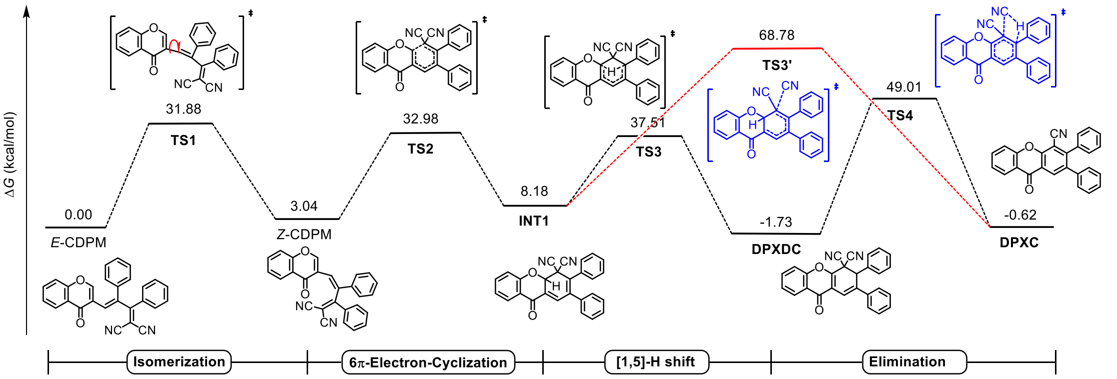

Supplementary Fig. 37 Energy profile for the thermal reactions of *Z*-CDPM calculated at the TPSS/6-31G(d,p) level without solvent correction.

2. Solvation Effects:

The *Z* and *E* isomers exhibit subtle steric and π-conjugation differences that may be sensitive to specific solvent interactions. It is important to note that the conversion from *Z*-CDPM to DPXC occurs under extended heating while adsorbed on silica gel. For simplicity, our initial calculations of the key points on the potential energy surface were performed in the gas phase, without accounting for solvation effects. When implicit solvation was included, the Gibbs free energy difference between *Z*- and *E*-CDPM was reduced to ΔG = 1.92 kcal·mol^{-1}, corresponding to an equilibrium distribution of 90.98% vs. 9.02% (based on the Boltzmann distribution). However, it should be emphasized that such effects are challenging to fully capture using standard implicit solvation models.

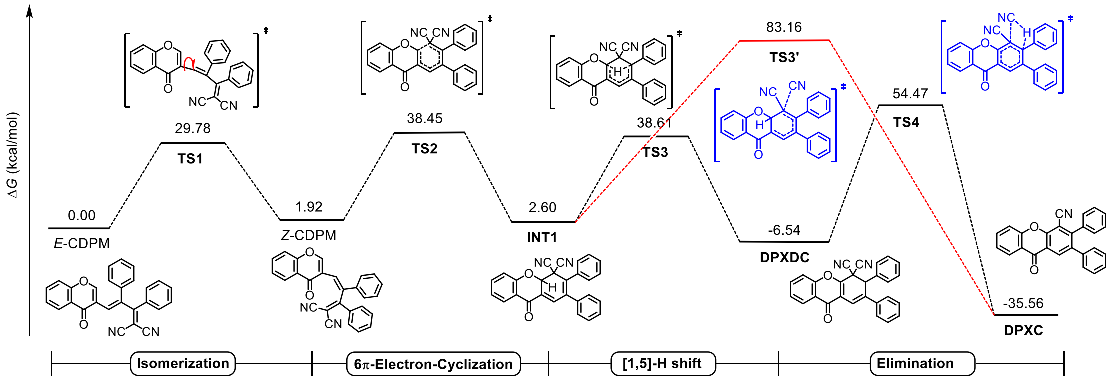

Supplementary Fig. 36 Energy profile for the thermal reactions of *Z*-CDPM calculated at the M06-2X/6-31G(d,p) level with solvent correction.

3. Experimental Conditions Are Not Ideal Equilibrium Systems:

The isomerization process in our system occurs under thermal conditions where continuous conversion to downstream products such as DPXDC takes place. This ongoing transformation likely prevents the establishment of true thermodynamic equilibrium between the *Z*- and *E*-isomers. Furthermore, the observed isomer distribution is strongly influenced by kinetic factors and the non-equilibrium nature of the experimental conditions, such as photochemical excitation and solid-state heating. These factors collectively limit the system's ability to reach and maintain a stable equilibrium state.

We have revised the manuscript to explicitly acknowledge this mismatch and added the following clarification:

"*Although DFT calculations suggest a 1.92 kcal·mol^{-1} free energy difference between Z- and E-CDPM, this value falls within the typical error margin of DFT methods and may not reliably reflect the experimental isomeric distribution, especially under non-equilibrium conditions.*"

- Further, saying there is a control for the process of Z to E or E to Z isomerization in CDPM is overstating because that is simply an equilibration and it does apparently not lead to significant accumulation of one isomer – so that is also not really usable to change any luminescent properties.

Response: Thank you for this thoughtful comment. We agree with the reviewer that the thermal *Z*/*E* isomerization of CDPM occurs as a dynamic equilibrium process under heating, and does apparently not lead to the accumulation of a single isomer. Therefore, the ability to precisely enrich one isomer over the other via thermal treatment is indeed limited.

Our original use of the term "controllable isomerization" was intended to describe the fact that thermal stimulation reproducibly induces *Z*/*E* interconversion, and that this process could be consistently

triggered and monitored under defined thermal conditions. However, we acknowledge that this does not imply selective or directional control over the final isomer ratio, nor does it yield a functionally distinct photoluminescence output between the equilibrated states.

To avoid overstatement, we have revised the corresponding description in the manuscript to clarify that: *"the similar thermodynamic stability of two isomers induced a thermal Z/E isomerization process and leads to a near-equilibrium distribution for CDPM, while its utility in modulating luminescent output is limited because of the inability to significantly accumulate one isomer."*

We thank the reviewer for pointing out this important distinction, and we believe the revised wording more accurately reflects the observed behavior.

- On the same note: the equilibrium isomer distribution Z/E needs to be assessed in Figure 2f the isomers are not equilibrated when starting from either Z or E. the equilibrium distribution would directly give the delta G value between the two isomers from experiment to compare to DFT.

Response: Thanks a lot for your careful review and comments. We appreciate the reviewer's insightful suggestion. Indeed, directly determining the equilibrium distribution of *Z*/*E*-CDPM from independent starting points would allow extraction of the experimental Gibbs free energy difference (ΔG) for comparison with DFT predictions.

However, in the current study, the isomerization process is often accompanied by secondary thermal reactions (e.g., formation of DPXDC), as observed in prolonged heating experiments. This competing conversion introduces a significant complication: the *Z*/*E* ratio measured at extended time points may not reflect a true thermodynamic equilibrium between *Z*- and *E*-CDPM alone, but rather a dynamic system where multiple processes (isomerization, decomposition, or rearrangement) occur simultaneously or sequentially.

In Figure 2f, although partial interconversion between *Z* and *E* is observed, the system does not fully equilibrate before further transformation begins. Therefore, deriving an accurate ΔG value from this data would require kinetic separation of the isomerization and decomposition steps, which is beyond the scope of the current dataset.

We have revised the manuscript to acknowledge this limitation and added the following sentence:

"*While the partial interconversion between Z- and E-CDPM is observed experimentally, the presence of subsequent thermal reactions (e.g., formation of DPXDC) complicates the accurate extraction of an equilibrium Z/E ratio. Therefore, a reliable experimental estimation of ΔG is currently not feasible without kinetic deconvolution.*"

- The authors claim an endothermal process when going from Z/E CDPM to DPXDC. but the cyclized product is more stable. So that is certainly not an endergonic process- do the authors suggest an entropy driven reaction or how do they interpret their data correctly?

Response: Thanks a lot for your careful review and comments. Both the DSC data in Supplementary Fig. 17 and the Energy profile in Fig. 4e suggested an exothermic process, we have revised this claim and changed the description into "The DSC curve of *Z*-CDPM exhibited a remarkable peak at 178 ℃, suggesting an exothermic thermal cyclization process" in the main text. We thank the reviewer for pointing out this mistake.

- I am also not sure that the observe different heating behavior in solution and on silica solid support is really caused by the "morphology changes". It could well be the acidity of the environment. Please

test whether pH changes in solution lead to different thermal and photochemical outcomes of the different isomers. Also test if basic alumina support give the same results.

Response: Thanks a lot for your careful review and comments. According to your valuable suggestions, we have added acetic acid to the $C_2D_2Cl_4$ solution of *Z*-CDPM and then heated it at 145 °C for 1 d. The results are added in Supplementary Fig. 33 in the supporting information, which indicate that pH changes in solution does not accelerate the generation of DPXC. Also, we have tested the thermal switching efficiency of *Z*-CDPM in basic alumina support, and the results in Supplementary Fig. 32 in the supporting information are comparable with that of silica gel support, validating the efficient formation of DPXC in solid support. Likely, these results supported the different heating behavior of *Z*-CDPM through "morphology changes".

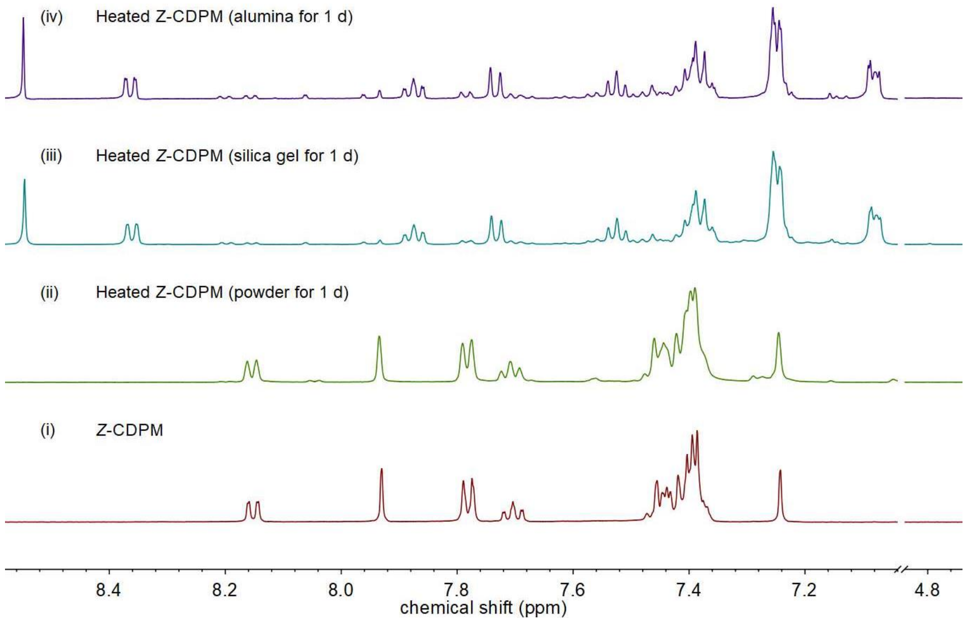

Supplementary Fig. 32 ^{1}H NMR spectra of *Z*-CDPM powder before and after heating at 145 °C in different media for 1 d.

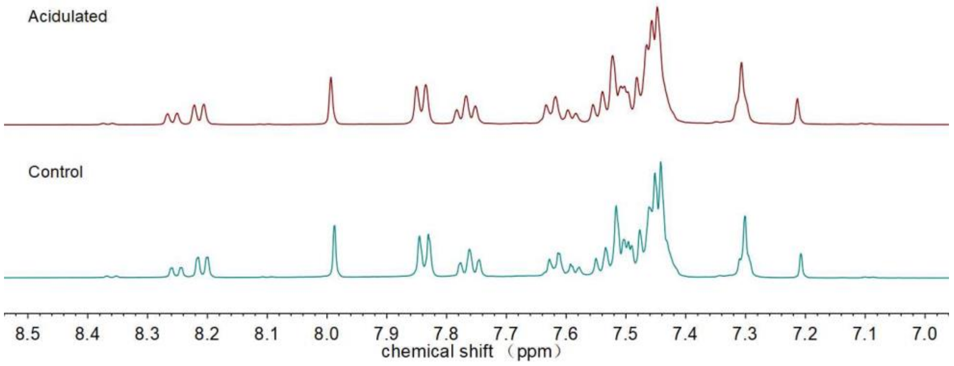

Supplementary Fig. 33. ^{1}H NMR spectra of *Z*-CDPM before and after acetic acid (0.5%, v/v) acidification in $C_2D_2Cl_4$. The sample was heated at 145 °C for 1d.

- On the same note, with proper choice of solvent many thermal processes could be influenced dramatically giving the possibility to obtain better control and introducing reversibility for

cyclization reactions. Such effects are well known in photochromes for example see here: doi: 10.1038/s41467-019-12463-4; doi: 10.1021/jacs.1c11183; doi:10.1021/jacs.4c06377 I would urge the authors to assess highly polar versus completely apolar solvents for that purpose.

Response: We sincerely appreciate the reviewer's insightful suggestion regarding the exploration of solvent effects on the thermal processes in our study. The potential impact of solvent polarity on reaction control and reversibility is intriguing, as exemplified by the cited references. In our original experiments, solvent with low polarity ($C_2D_2Cl_4$ and diphenyl ether) was utilized for the NMR experiment and the synthetic procedure in this work. Thus, high polar solvent like DMSO and 1,2-propanediol was further chosen and applied for the synthesize of the thermal cyclization product DPXDC through annealing. However, the reaction solution turned out to be a messy and impossible to analyze under similar reaction conditions. Notably, when synthesizing *Z*/*E*-CDPM in pyridine (a moderately polar solvent) under annealing, the reaction time was prolonged from 2 h to overnight. However, the reaction yields of *Z*/*E*-CDPM were both dramatically dropped down, and no obvious signal of DPXDC was found as well. According to previous reports (*Chem. Heterocycl. Compd.* 1971, 7, 675; *J. Org. Chem.* 1981, 46, 4971; *Aggregate* 2022, 4, e239, *Angew. Chem. Int. Ed.* 2023, 62, e202300353), chromone derivatives are electrophilic, especially when substituted with electron-withdrawing groups, and are known to undergo undesired side reactions or degradation in highly polar media. Therefore, consistent with prior findings, our results indicate that *Z*/*E*-CDPM is not well-suited for thermal treatment in highly polar solvents, likely due to instability or uncontrolled reactivity under such conditions.

- With respect to the calculations: DFT values for Gibbs energies of activation also do not align with the experiments, not even qualitatively. In the experiment E to Z and Z to E isomerizations are faster than the follow up cyclization reactions. Thus, the order of barrier heights is upside down in the DFT calculations. Generally the calculated barriers are oftentimes extremely high beyond 45 kcal/mol, which would not allow to do these transformations at reasonable times even at temperatures of 145 -200°C. please calculate via the Eyring equation how long the heating processes would take based on the DFT barriers and compare to the actual experiments. For sake of simplicity you can assume first order rate laws.

Response: Thank you for this valuable comment. We fully agree that in our original DFT results (see original Fig. 4e), the calculated activation barriers appeared too high to account for the experimentally observed thermal transformations occurring within hours at 145–200 °C. In particular, the isomerization step (*E* ↔ *Z*) showed a significantly higher barrier than the cyclization step, which is inconsistent with the experimental observation that *E*/*Z* isomerization occurs faster than subsequent cyclization.

To address this discrepancy, we carefully re-examined the reaction coordinate associated with the *E* ↔ *Z* isomerization, and identified a new transition state (TS1) involving rotation around the central C–C single bond, which is more consistent with the expected pathway. Furthermore, we incorporated solvent effects ($C_2H_4Cl_2$) via the SMD model at the M06-2X/6-31G(d,p) level to better reflect the experimental conditions.

As shown in the updated energy profile (Supplementary Fig. 36 in the Supporting Information), the revised barrier for *E* → *Z* isomerization is now 29.78 kcal·mol^{-1}, significantly lower than the original value of 46.62 kcal·mol^{-1}. This adjustment brings the relative barrier heights into much better agreement with experimental kinetics: isomerization is now thermally accessible and occurs faster than the subsequent 6π-electron cyclization (TS2 = 38.45 kcal·mol^{-1}).

To further evaluate the timescale of these transformations, we applied the Eyring equation under the

assumption of first-order kinetics to estimate the reaction half-life. For the updated TS1 barrier (29.78 kcal·mol^{-1}), the calculated half-life at 145 °C is approximately 4.86 minutes, which is consistent with an extremely rapid isomerization process. Moreover, a reaction with an activation barrier of 38.61 kcal·mol^{-1} at the same temperature yields an estimated half-life of approximately 139.19 days. While this value is longer than the experimentally observed transformation time of ~30 days for the conversion of *E*-CDPM to DPXDC, the result is reasonably consistent when considering the assumptions of first-order kinetics and the inherent uncertainties in DFT-based barrier calculations. Although slow, such a transformation remains thermally accessible and may proceed gradually but measurably over extended periods. This supports the plausibility of the revised energy profile and validates our updated computational model in capturing the experimentally observed reactivity trends. It is also important to note that the final transformation step from DPXDC to DPXC, which occurs under silica-gel-assisted heating, involves a calculated barrier as high as 54.47 kcal·mol^{-1}. While this value appears unreasonably high under conventional conditions, it likely reflects the limitations of our simplified model in capturing the complex surface-assisted processes that occur upon immobilization on silica. Therefore, some deviation from experimental behavior in this step is understandable and expected.

We have now updated the manuscript to reflect these corrections and added a comparison of the original and revised energy profiles (see Supplementary Figs. 35 and 36 in the Supporting Information). We believe this revision significantly improves the reliability and consistency of the theoretical analysis.

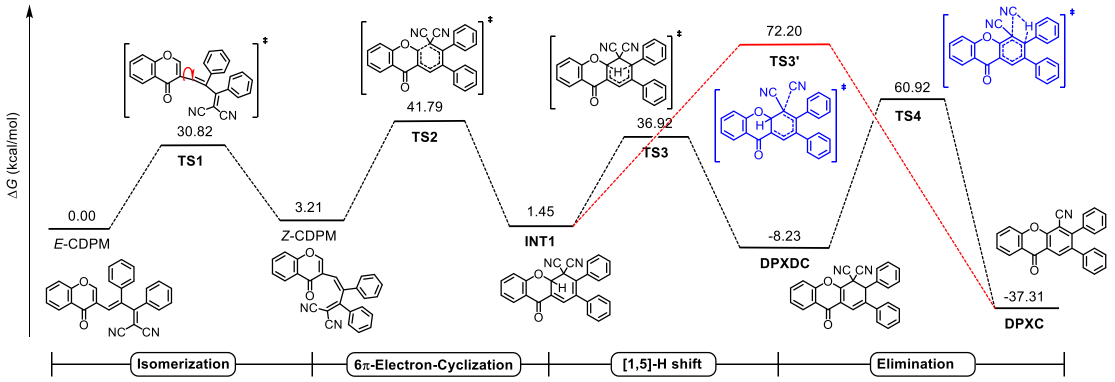

Supplementary Fig. 35 Energy profile for the thermal reactions of *Z*-CDPM calculated at the M06-2X/6-31G(d,p) level without solvent correction.

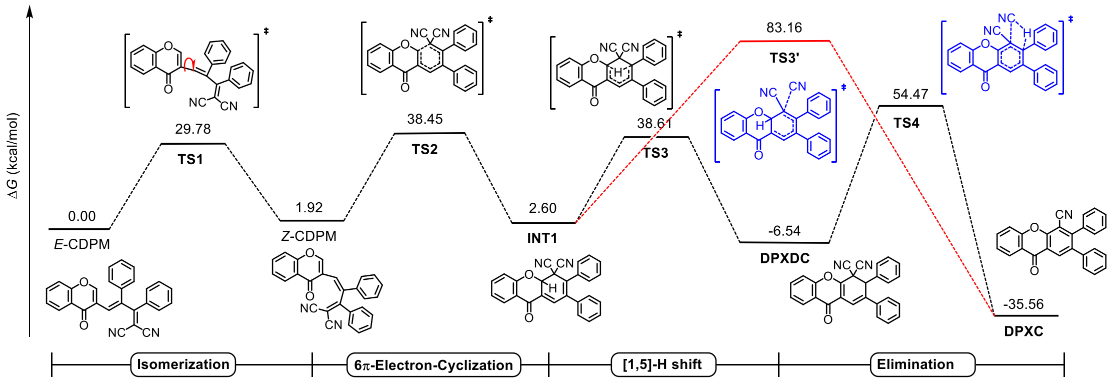

Supplementary Fig. 36 Energy profile for the thermal reactions of *Z*-CDPM calculated at the M06-2X/6-31G(d,p) level with solvent correction.

- Further what is odd is that the authors use the E-isomer as point 0 and thus obtain negative delta

G for the cyclized isomers. Please use the global minimum as 0 to compare all isomers properly. Please also provide a scheme where all minima of the six isomers are compared side by side.

Response: Thanks a lot for your careful review and comments. We understand the reviewer's concern regarding the choice of the *E*-isomer as the reference point (0 kcal·mol^{-1}) in our energy diagrams, which may affect direct comparison of the relative stabilities of all isomers.

However, our rationale for selecting *E*-CDPM as the energy reference is that it serves as the starting reactant in the reaction pathways discussed throughout the main text (Fig. 1b and Fig. 4e). The mechanistic analysis, particularly for the photochemical transformation, is logically constructed beginning from *E*-CDPM as the experimentally used precursor. Changing the zero-point reference to a later-stage product (e.g., DPXDC or DPXC), although thermodynamically more stable, would disrupt the flow of the reaction energy landscape and complicate the interpretation of uphill/downhill processes in the early-stage transformations.

For clarity and completeness, we have now provided Supplementary Fig. 59 that presents the relative Gibbs free energies of all six ground-state isomers (*Z*-CDPM, *E*-CDPM, DPXDC, DPXC, HBNPMM, and DPPCYM), aligned to a common reference point (DPXC as the global minimum), allowing side-by-side comparison as requested. Nonetheless, in the main mechanistic discussions, we have retained *E*-CDPM as the energy reference point to preserve internal consistency with the narrative structure of the manuscript.

We believe this revision improves the clarity and consistency of our thermodynamic analysis and ensures a fair comparison across all isomers. The manuscript and Supporting Information have been updated accordingly.

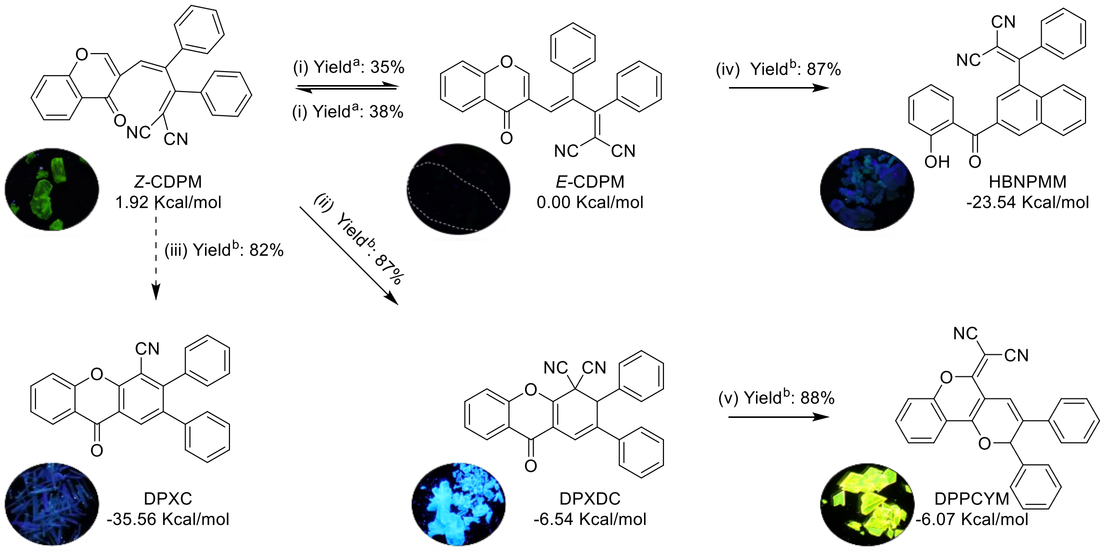

Supplementary Fig. 59 The thermal and photo reactions of *Z*-CDPM under different conditions: (i) annealing at 145 °C in $C_2D_2Cl_4$ for 1 d; (ii) annealing at 200°C in diphenyl ether for 2 h; (iii) annealing at 145 °C in silica gel for 1 d; (iv, v) UV irradiation under a 365 nm UV lamp (0.12 mW/cm^2) at room temperature in $C_2D_2Cl_4$ for 2 h. Insets: photographs of crystals taken under UV irradiation and the relative Gibbs free energies of all six ground-state isomers. [a] Conversion yield determined by ^{1}H NMR data. [b] Conversion yield determined by isolation of products.

- Generally when talking about AIE, from the alpha-values it is not clear how good the AIE performance is. please show quantitative data- what is the intensity increase factor? How is alpha

AIE defined? is the concentration always the same or is that dilution (by what factor) of THF solution with water?

Response: Thanks a lot for your careful review and comments. We fully agree that quantitative data is necessary to illustrate the AIE performance of luminogens. According to your valuable suggestions, we have added the definition of α_{AIE}, the concentration and the intensity increase factor in the main text. Specifically, the emission peak at a water fraction (f_w) of 99% is chosen as the reference and then the corresponding PL intensity (I) is measured. Since the molecular aggregation behavior is positively correlated with PL intensity, it is set as the intensity increase factor. Accordingly, the PL intensity in pure THF solution is selected as the denominator (I_0), and I/I_0 values is defined as α_{AIE} in this work. Meanwhile, the concentrations of all samples were fixed at 10 μM for quantitative comparison.

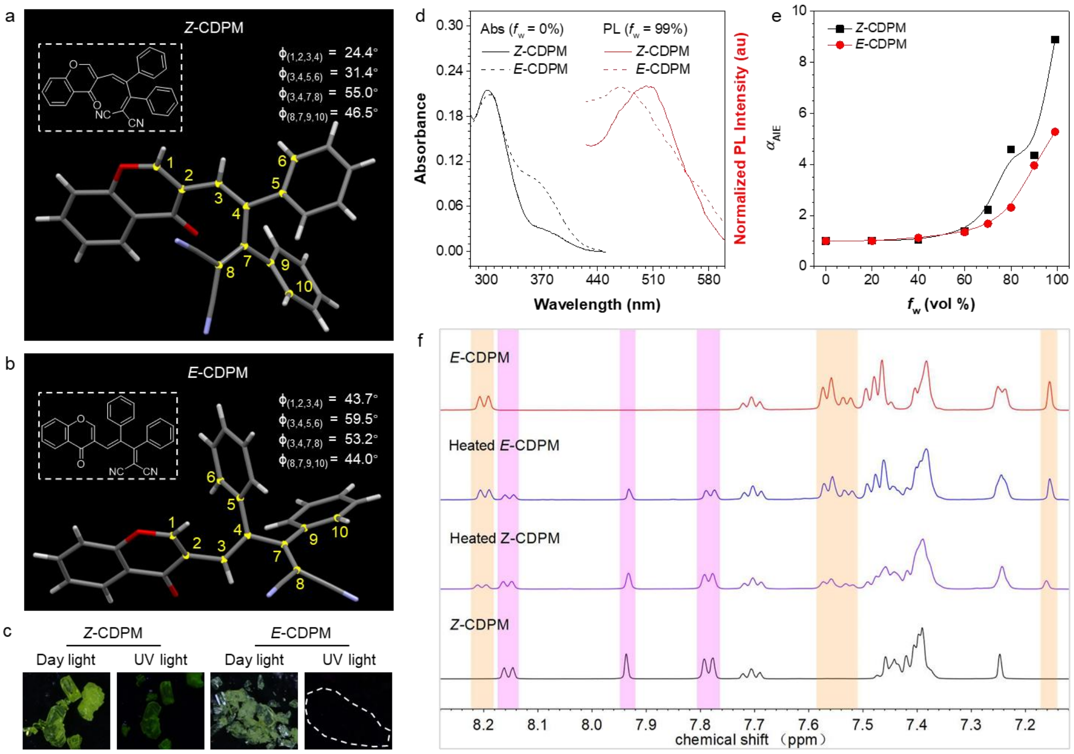

Fig. 2 Photophysical properties and *Z*/*E* isomerization. Single crystal structures of (a) *Z*-CDPM and (b) *E*-CDPM. Insets: chemical structures and dihedral angles in the crystal structures. (c) Photographs of crystals of *Z*-CDPM and *E*-CDPM. (d) Absorption and normalized photoluminescence (PL) spectra of *Z*-CDPM (solid line, 10 μM) and *E*-CDPM (dash line, 10 μM) in THF/water mixtures with different water fractions (f_w). (e) Plots of I/I_0 values (defined as α_{AIE}) versus the f_w of *Z*-CDPM (black, 10 μM, $\lambda_{ex/em}$ = 365/505 nm) and *E*-CDPM (red, 10 μM, $\lambda_{ex/em}$ = 350/480 nm), where I_0 = PL intensity in pure THF solution at the emission maximum wavelength. (f) ^{1}H NMR spectra of *Z*-CDPM and *E*-CDPM before and after heating at 145 °C in $C_2D_2Cl_4$ for 1d. *Z*-CDPM was highlighted in purple and *E*-CDPM was highlighted in orange.

- Also generally the luminescence data and AIE behaviors are oftentimes presented without numerals on the y-axes of the diagrams in most Figures throughout the manuscript and the SI. Please add the corresponding numbers! Quantify at least relatively the AIE increase in luminosity and also the luminosity changes between isomers in solution and solid states.

Response: Thanks a lot for your careful review and comments. We have added the corresponding

numbers on the y-axes of the diagrams in the main text and the supporting information according to your valuable suggestions. Also, we acknowledge that quantitative data is beneficial for studying the luminescence and AIE behaviors of luminogens. Thus, we have summarized the photophysical properties of these six AIEgens and added the corresponding overview Figures in the supporting information for better understanding.

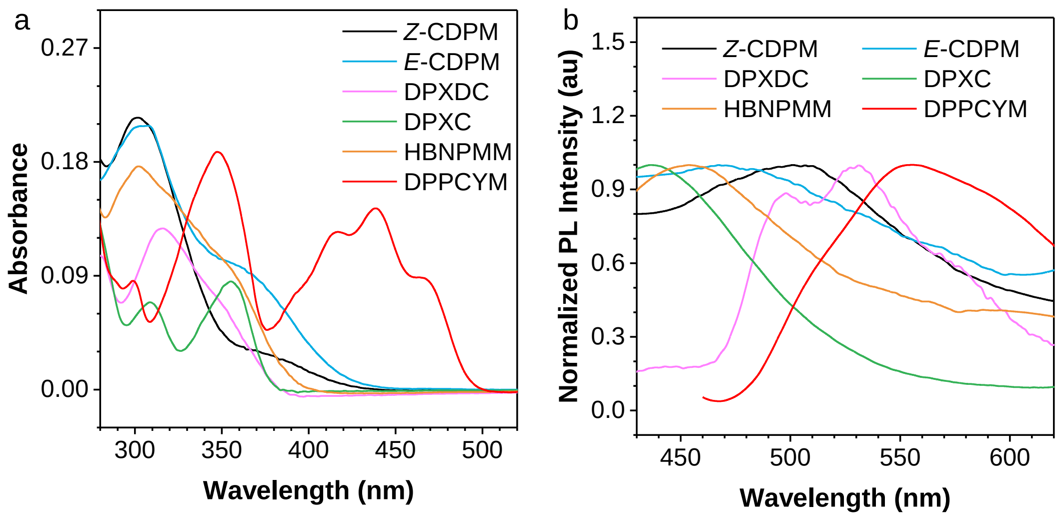

c

Compound	λ_{ab} [nm][a]	ε [$M^{-1}\cdot cm^{-1}$]	λ_{em} [nm][b]	Quantum yield [%]	
				Aggregate[c]	Crystal
Z-CDPM	302	21497	505	1.0	2.1
E-CDPM	308	20867	480	0.2	0.5
DPXDC	315	12730	495	8.2	14.9
DPXC	355	8560	440	3.3	2.8
HBNPMM	303	17634	460	1.0	0.9
DPPCYM	440	14269	550	4.5	37.8

[a] Absorption maximum was measured in THF solution. Concentration= 10 μM. [b] Emission maximum was measured in THF/water mixture (f_w =99%). Concentration= 10 μM. [c] Quantum yield was measured in in THF/water mixture (f_w =99%).

Figure S39. (a) Absorption of AIEgens (10 μM) in THF solution. (b) Normalized PL spectra of AIEgens (10 μM) in THF/water mixtures (f_w = 99%). (c) Summary of photophysical properties of Z-CDPM and its isomers.

- Important literature on multi-stimuli responsive systems is missing, that need to be cited to put this work in proper context. For example, multi-photochromes are a large developed field already and important early work is not cited at all here: a) A. Fihey, A. Perrier, W. R. Browne, D. Jacquemin, Chem. Soc. Rev. 2015, 44, 3719–3759; b) H. Nie, J. L. Self, A. S. Kuenstler, R. C. Hayward, J. Read de Alaniz, Adv. Opt. Mater. 2019, 7; c) D. Gust, J. Andreasson, U. Pischel, T. A. Moore, A. L. Moore,

Chem. Commun. 2012, 48, 1947–1957; d) J. Andreasson, U. Pischel, Chem. Soc. Rev. 2018, 47, 2266–2279; e) X. Fu, L. Hosta-Rigau, R. Chandrawati, J. Cui, Chem 2018, 4, 2084–2107; f) M. Wei, Y. Gao, X. Li, M. J. Serpe, Polym. Chem. 2017, 8, 127–143. g) J. Andreasson, U. Pischel, S. D. Straight, T. A. Moore, A. L. Moore, D. Gust, J. Am. Chem. Soc. 2011, 133, 11641–11648; h) F. Tian, D. Jiao, F. Biedermann, O. A. Scherman, Nat. Commun. 2012, 3, 1207; i) M. Herder, M. Utecht, N. Manicke, L. Grubert, M. Pätzel, P. Saalfrank, S. Hecht, Chem. Sci. 2013, 4, 1028–1040; j) M. Li, S. Yang, W. Liang, X. Zhang, D. Qu, Dyes Pigm. 2019, 166, 239–244; e) T. Schmitt, L. Y. Hsu, N. Oberhof, D. Rana, A. Dreuw, E. Blasco, P. Tegeder, Adv. Funct. Mater. 2023; k) J. Andréasson, S. D. Straight, T. A. Moore, A. L. Moore, D. Gust, J. Am. Chem. Soc. 2008, 130, 11122–11128; l) H. Nishioka, X. Liang, T. Kato, H. Asanuma, Angew. Chem. Int. Ed. 2012, 51, 1165–1168; m) H. Dube, J. Rebek Jr., Angew. Chem. Int. Ed. 2012, 51, 3207–3210; n) K. Hiltebrandt, T. Pauloehrl, J. P. Blinco, K. Linkert, H. G. Borner, C. Barner-Kowollik, Angew. Chem. Int. Ed. 2015, 54, 2838–2843; j) M. Skugor, J. Valero, K. Murayama, M. Centola, H. Asanuma, M. Famulok, Angew. Chem. Int. Ed. 2019, 58, 6948–6951; o) V. X. Truong, J. Bachmann, A. N. Unterreiner, J. P. Blinco, C. Barner-Kowollik, Angew. Chem. Int. Ed. 2022, 61, e202113076; p) D. Manna, T. Udayabhaskararao, H. Zhao, R. Klajn, Angew. Chem. Int. Ed. 2015, 54, 12394–12397; q) F. Zhao, L. Grubert, S. Hecht, D. Bleger, Chem. Commun. 2017, 53, 3323–3326; r) M. Drohse Kilde, L. Brolos, M. Manso, J. Mogensen, C. Gregers Tortzen, M. Brondsted Nielsen, Chem. Eur. J. 2020, 26, 13429–13435; s) M. Dowds, S. G. Stenspil, J. H. de Souza, B. W. Laursen, M. Cacciarini, M. B. Nielsen, ChemPhotoChem 2022, 6; t) M. M. Lerch, M. J. Hansen, W. A. Velema, W. Szymanski, B. L. Feringa, Nat. Commun. 2016, 7, 12054; u) N. M. Wu, M. Ng, V. W. Yam, Nat. Commun. 2022, 13, 33; r) A. Gerwien, B. Jehle, M. Irmler, P. Mayer, H. Dube, J. Am. Chem. Soc. 2022, 144, 3029–3038; s) G. R. Wilson, K. C. Park, G. C. Thaggard, C. R. Martin, A. R. Hill, J. Haimerl, J. Lim, B. K. P. Maldeni Kankanamalage, B. J. Yarbrough, K. L. Forrester, R. A. Fischer, P. J. Pellechia, M. D. Smith, S. Garashchuk, N. B. Shustova, Angew. Chem. Int. Ed. 2023, 62, e202308715; v) J. Sheng, J. Perego, S. Bracco, P. Cieciorski, W. Danowski, A. Comotti, B. L. Feringa, Angew. Chem. Int. Ed. 2024, e202404878. w) Köttner, Dube, Angew. Chem. Int. Ed. 2024, 63, e202409214

- When talking about ring-cyclization and in particular ring flipping reactions there also needs mentioning of recent works of aza-diarylethenes with multi-state behavior in such “flipping” manner: doi:10.1021/jacs.3c11803;doi:10.1002/adma.202411223; doi:10.1002/anie.202415961

Response: Thanks a lot for your careful review and comments. We have appropriately added these references in the main text according to your valuable suggestions.

- The authors use inflated language quite a bit and I would urge them to tone down their claims, oftentimes the used terms are either not scientific or seem to be used just for buzz-word sake. Examples are: “energy isolation” to describe selectivity in thermal steps that are simply based on sufficiently different Gibbs energies of activation for these processes; “fantastic ring switching”; “morphology tuning” to describe the switching on solid support instead of switching in solution; “advanced artificial intelligence technologies” there is no clear connection to AI with such work…

Response: We sincerely appreciate the reviewer's constructive feedback regarding the tone and language used in our manuscript. We acknowledge that certain phrases may have inadvertently overstated our findings or relied on non-scientific terminology. To address these concerns and ensure clarity, precision, and scientific rigor, we have deleted the description of “energy isolation” and “morphology tuning” in

the main text, and deleted the word "fantastic" from the sentence "fantastic ring switching". Also, the sentence " advanced artificial intelligence technologies " is replaced by "advance information encryption technology" in the main text to better describe its application scope.

Minor points:

- "Subsequently, the fantastic ring-switching" please tone down.

Response: Thanks a lot for your careful review and comments. We have revised such statement into "the ring-switching" in the main text according to your valuable suggestion.

- "Photophysical properties and Z/E isomerization. Z-CDPM and E-CDPM were simultaneously synthesized via a facile two-step process" please state here whether you have isolated Z and E and studied them separately or if that was done in mixtures… - also make that clear when talking about crystal structures.

Response: Thanks a lot for your careful review and comments. *Z*-CDPM and *E*-CDPM were successfully isolated in our work. To solve this concern, we have revised such statement and added the sentence "*Z-CDPM and E-CDPM were simultaneously synthesized via a facile two-step process (Supplementary Fig. 1), and successfully separated and purified one by one by column chromatography.*" in the main text to accurately convey the detailed experimental information.

- Fig. 2 a and b the actual isomerization is hard to see from the chemdraw structures. Please leave one fragment static and show the isomerization that way.

Response: Thanks a lot for your careful review and comments. According to your valuable suggestions, we have listed the isomerization reactions separately for the convenience of observation, as depicted in Fig. 2 in the main text.

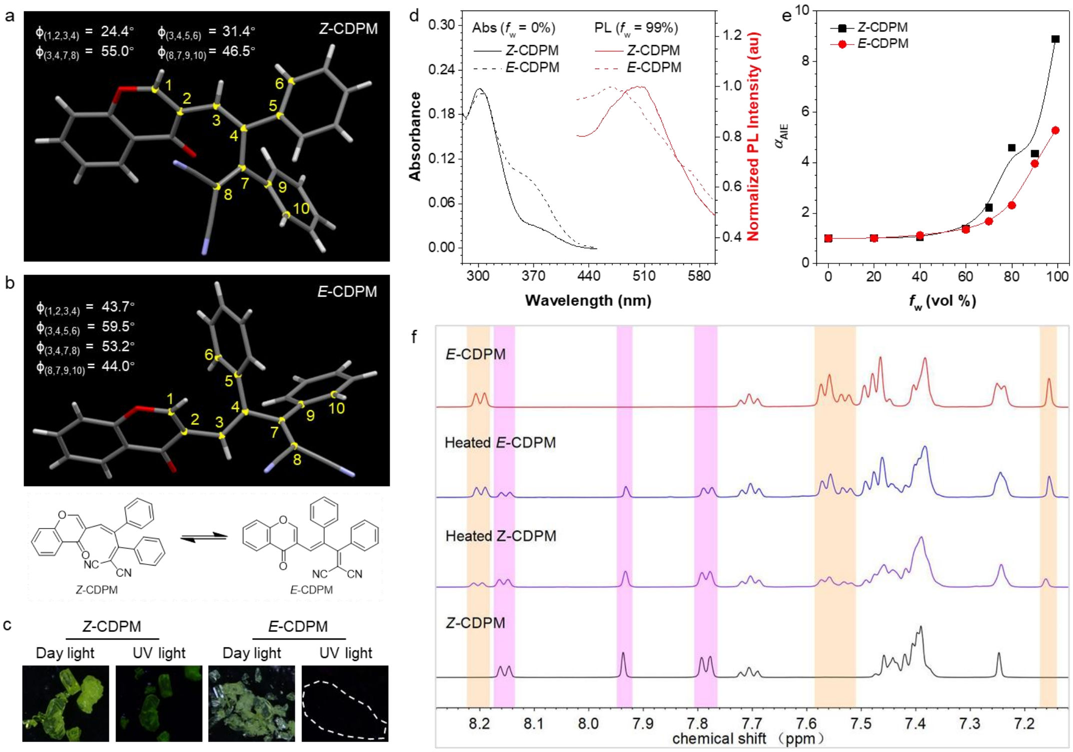

Fig. 2 Photophysical properties and *Z*/*E* isomerization. Single crystal structures of (a) *Z*-CDPM and (b) *E*-CDPM. Insets: chemical structures and dihedral angles in the crystal structures. (c) Photographs of crystals of *Z*-CDPM and *E*-CDPM. (d) Absorption and normalized photoluminescence (PL) spectra of *Z*-CDPM (solid line, 10 μM) and *E*-CDPM (dash line, 10 μM) in THF/water mixtures with different water fractions (f_w). (e) Plots of I/I_0 values (defined as α_{AIE}) versus the f_w of *Z*-CDPM (black, 10 μM, $\lambda_{ex/em}$ = 365/505 nm) and *E*-CDPM (red, 10 μM, $\lambda_{ex/em}$ = 350/480 nm), where I_0 = PL intensity in pure THF solution at the corresponding emission peak. (f) ^{1}H NMR spectra of *Z*-CDPM and *E*-CDPM before and after heating at 145 °C in $C_2D_2Cl_4$ for 1d. *Z*-CDPM was highlighted in purple and *E*-CDPM was highlighted in orange.

- "When a poor solvent of water was added…" what does that mean?

Response: Thank you very much for your thoughtful comment. We agree that the original phrase "a poor solvent of water" was imprecise and potentially misleading. This has been revised in the manuscript for clarity. In the context of AIE studies, water is commonly referred to as a "poor solvent" because most organic luminogens, including our AIEgens, exhibit very low solubility in aqueous environments. Typically, a stock solution of the AIEgen (1.0 mM) is first prepared in pure THF (a good solvent), and then gradually mixed with increasing amounts of water to form THF/water mixtures (10 μM) with varying water fractions (f_w). As the water fraction increases, the reduced solubility of the luminogens promotes molecular aggregation, thereby activating aggregation-induced emission.

Therefore, the term "poor solvent" in this context specifically refers to the limited solubility of the AIEgens in this solvent.

- "Z/E isomerization was demonstrated for CDPM, as well as the similar thermodynamic stability of two isomers" again 3.4 kcal/mol is not really similar and would lead to more than 95% isomer accumulation under thermal equilibrium even at very high temperatures.

Response: Thank you very much for this critical observation. We agree that a free energy difference of 3.4 kcal·mol^{-1} would, under ideal thermal equilibrium conditions, result in a significant bias toward one isomer. In fact, our original calculations yield a ΔG = 3.21 kcal·mol^{-1} (rather than 3.4 kcal·mol^{-1}), which would still theoretically favor one isomer overwhelmingly (97.94% vs. 2.06%, based on the Boltzmann distribution) at 145 °C under ideal equilibrium conditions.

This discrepancy likely reflects the limitations of static DFT calculations in capturing the full thermodynamic landscape of real systems. Several factors may contribute:

1. DFT Method Error Range:

DFT inherently involves approximations, and the error in computed free energy differences can be up to ±2–4 kcal·mol^{-1} depending on the functional and the system. The 3.21 kcal·mol^{-1} value lies within this uncertainty range and may not reliably reflect small thermodynamic preferences. As shown below, after change the DFT functional from M062X to TPSS, the energy difference change from 3.21 kcal·mol^{-1} to 3.04 kcal·mol^{-1}.

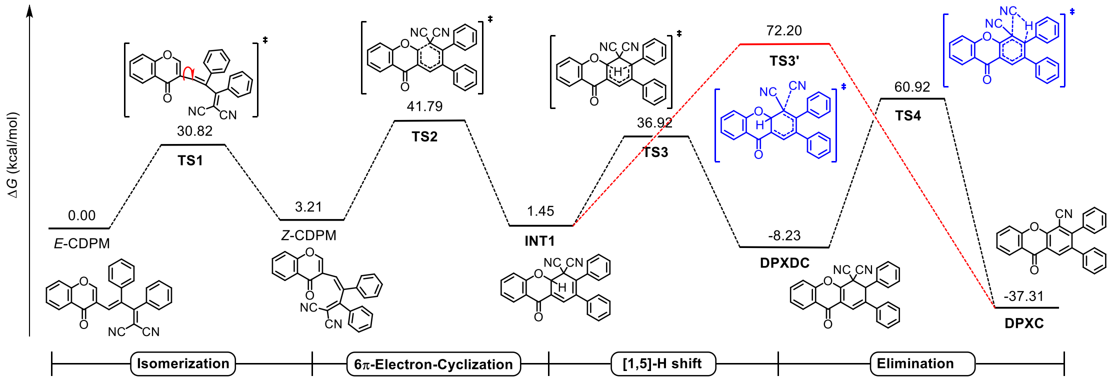

Supplementary Fig. 35 Energy profile for the thermal reactions of *Z*-CDPM calculated at the M06-2X/6-31G(d,p) level without solvent correction.

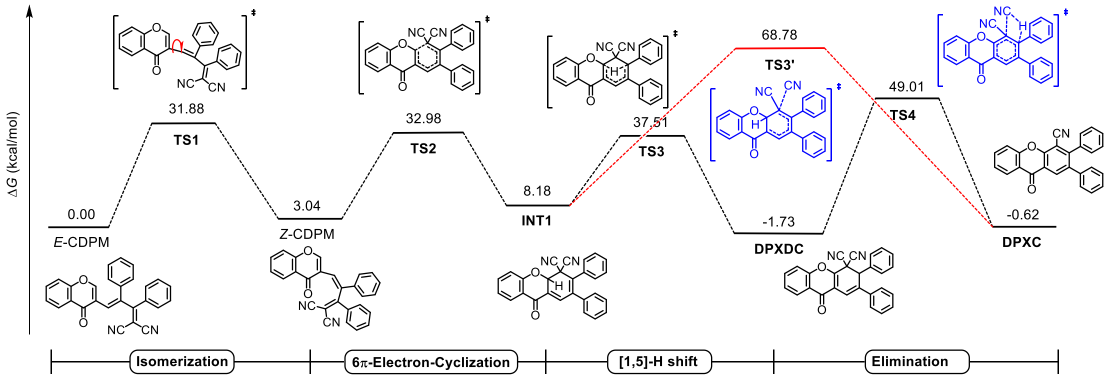

Supplementary Fig. 37 Energy profile for the thermal reactions of *Z*-CDPM calculated at the TPSS/6-31G(d,p) level without solvent correction.

2. Solvation Effects:

The *Z* and *E* isomers exhibit subtle steric and π-conjugation differences that may be sensitive to specific solvent interactions. It is important to note that the conversion from *Z*-CDPM to DPXDC occurs under extended heating while adsorbed on silica gel. For simplicity, our initial calculations of the key points on the potential energy surface were performed in the gas phase, without accounting for solvation effects. When implicit solvation was included, the Gibbs free energy difference between *Z*- and *E*-CDPM was reduced to ΔG = 1.92 kcal·mol^{-1}, corresponding to an equilibrium distribution of 90.98% vs. 9.02% (based on the Boltzmann distribution). However, it should be emphasized that such effects are challenging to fully capture using standard implicit solvation models.

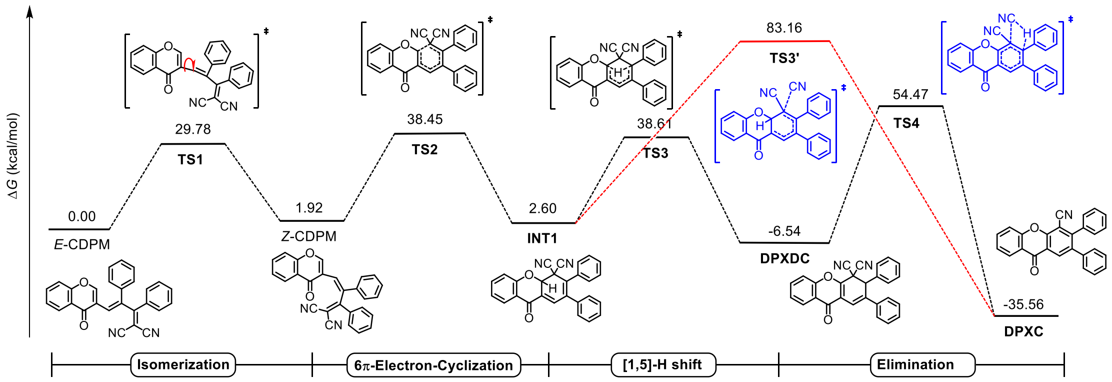

Supplementary Fig. 36 Energy profile for the thermal reactions of *Z*-CDPM calculated at the M06-2X/6-31G(d,p) level with solvent correction.

3. Experimental Conditions Are Not Ideal Equilibrium Systems:

The isomerization process in our system occurs under thermal conditions where continuous conversion to downstream products such as DPXDC takes place. This ongoing transformation likely prevents the establishment of true thermodynamic equilibrium between the *Z*- and *E*-isomers. Furthermore, the observed isomer distribution is strongly influenced by kinetic factors and the non-equilibrium nature of the experimental conditions, such as photochemical excitation and solid-state heating. These factors collectively limit the system's ability to reach and maintain a stable equilibrium state.

We have revised the manuscript to explicitly acknowledge this mismatch and added the following clarification:

"*Although DFT calculations suggest a 1.92 kcal·mol^{-1} free energy difference between Z- and E-CDPM, this value falls within the typical error margin of DFT methods and may not reliably reflect the experimental isomeric distribution, especially under non-equilibrium conditions.*"

- "As the reaction energy (ΔG = 8.09 kcal·mol-1) was similar to that of E-CDPM (ΔG = 7.43 kcal·mol-1), the comparable thermodynamic stability of these two isomers was confirmed again." please state here explicitly, that you are talking about Z and E CDPM.

Response: Thanks a lot for your careful review and comments. We have revised the sentence in the manuscript to explicitly clarify that the comparison is between Z-CDPM and E-CDPM. The revised sentence now reads: "*As the reaction energy (ΔG = 8.09 kcal·mol^{-1}) of Z-CDPM was similar to that of E-CDPM (ΔG = 7.43 kcal·mol^{-1}), the comparable thermodynamic stability of these two isomers, Z- and E-CDPM, was confirmed again.*" This change has been made in the revised manuscript to ensure clarity for readers.

- What is the yield of DPXC formation?

Response: Thanks a lot for your careful review and comments. According to your valuable suggestion, we have added the reaction yield of DPXC formation (82%) in Supplementary Fig. 59 in the supporting information.

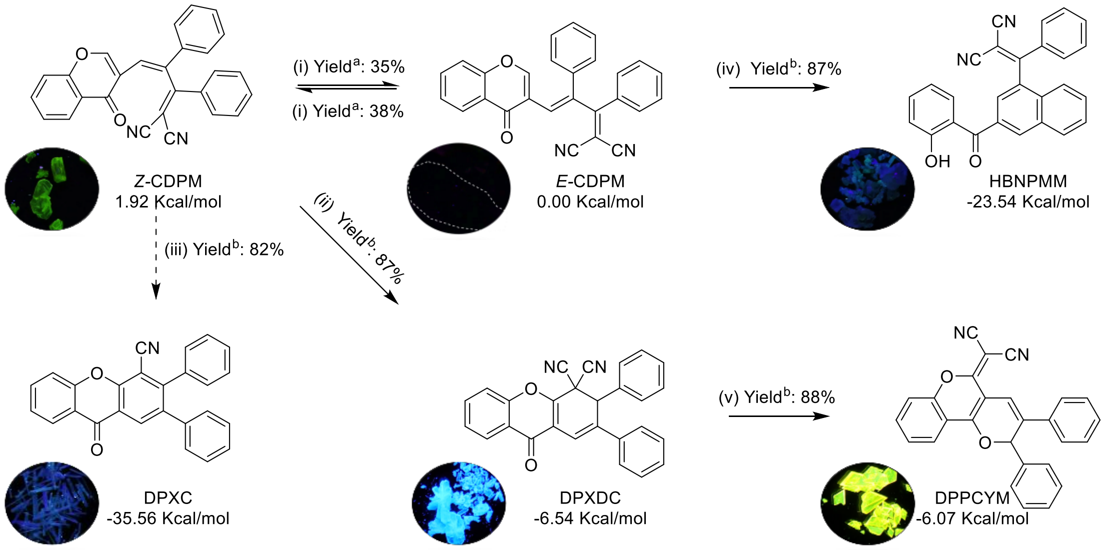

Supplementary Fig. 59 The thermal and photo reactions of *Z*-CDPM under different conditions: (i) annealing at 145 °C in $C_2D_2Cl_4$ for 1 d; (ii) annealing at 200°C in diphenyl ether for 2 h; (iii) annealing at 145 °C in silica gel for 1 d; (iv, v) UV irradiation under a 365 nm UV lamp (0.12 mW/cm^2) at room temperature in $C_2D_2Cl_4$ for 2 h. Insets:

photographs of crystals taken under UV irradiation. [a] Conversion yield determined by ^1H NMR data. [b] Conversion yield determined by isolation of products.

- Please state for all figures where DFT calculations are presented that the energy profiles are theoretical and also state the level of theory in the figure captions and in the manuscript text.

Response: Thanks a lot for your careful review and comments. We have now carefully revised the manuscript and figure captions accordingly. Specifically, we have clearly indicated in both the main text and the figure captions that the energy profiles were obtained from theoretical calculations. In addition, the level of theory used for all DFT calculations (M06-2X/6-31G(d,p) or TPSS/6-31G(d,p)) is now explicitly stated where appropriate. These clarifications are intended to enhance clarity and ensure transparency and reproducibility of the computational results.

- "Following, the photophysical properties of DPPCYM and the fluorescence response of Z-CDPM to UV irradiation were investigated." I believe the authors meant DPXDC instead of Z-CDPM?

Response: We sincerely thank the reviewer for the careful review and highlighting this mistake. We have revised the word "Z-CDPM" into "DPXDC" in the main text according to your valuable suggestions.

- "Indeed, the specific photophysical properties of DPXDC further confirmed the feasibility of performing discoloration in a single-luminogen system through the ring-flipping process." why discoloration? is not the product DPPCYM orange as stated above?

Response: We sincerely thank the reviewer for the careful review and highlighting this ambiguity. We acknowledge that using the term "discoloration" to describe the photoarrangement of DPXDC and the formation of DPPCYM would cause misunderstandings. Also, DPPCYM is orange as stated above. To solve this concern, we have revised this sentence into "*Considering the different photophysical properties of DPXDC and DPPCYM, the feasibility of performing visualized ring-flipping process in a single-luminogen system was demonstrated*" in the main text.

- "On the other hand, a gradually enhanced emission was detected for the UV irradiated sample of DPXDC in THF/water mixture with a fw of 99% (Fig. 6d)." how much? is not DPXDC also an AIEgen as stated above? Please provide quantitative comparison of the AIE properties of all isomers as requested in the major part above.

Response: Thanks a lot for your careful review and comments. This sentence discussed the discoloration behavior of DPXDC under UV irradiation in solution, including a comparison of its luminescence behavior with that of DPPCYM and the corresponding 68-fold increase in fluorescence intensity at 550 nm. While DPXDC is also an AIEgen, its photoarrangement product DPPCYM showed a stronger PL intensity in the THF/water mixture (f_w = 99%). To solve this concern, we have added the sentence "*While DPXDC is AIE-active, the fluorescence intensity increased 68 times at 550 nm and almost reached a plateau after 10 s of irradiation*" in the main text. Also, we have summarized the photophysical properties of these AIEgens and added the corresponding overview Figures in the supporting information to avoid misunderstanding. Moreover, quantitative comparison of the AIE properties of all isomers were added in the main text.

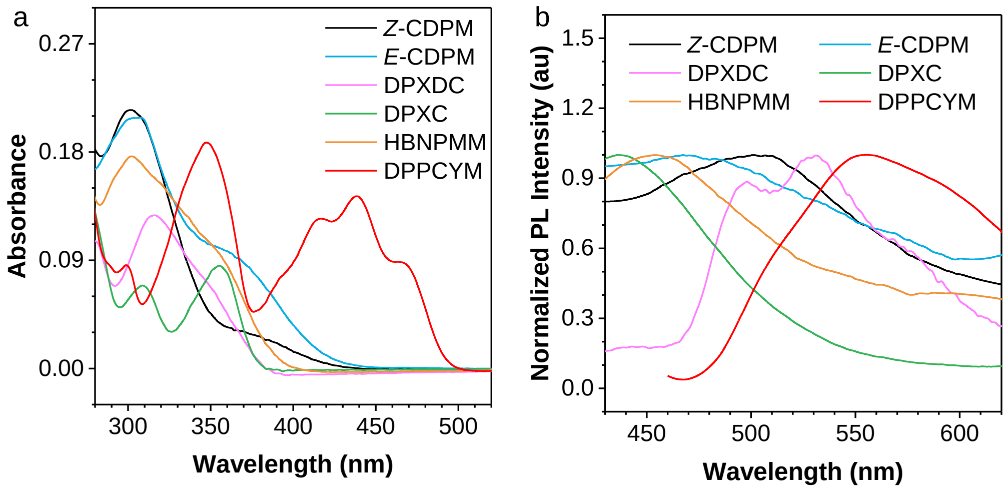

c

Compound	λ_{ab} [nm][a]	ε [$M^{-1}\cdot cm^{-1}$]	λ_{em} [nm][b]	Quantum yield [%]	
				Aggregate[c]	Crystal
Z-CDPM	302 (π→π*)	21497	505	1.0	2.1
E-CDPM	308 (π→π*)	20867	480	0.2	0.5
DPXDC	315 (π→π*)	12730	495	8.2	14.9
DPXC	355 (π→π*)	8560	440	3.3	2.8
HBNPMM	303 (π→π*)	17634	460	1.0	0.9
DPPCYM	440 (π→π*)	14269	550	4.5	37.8

[a] Absorption maximum was measured in THF solution. Concentration= 10 μM. [b] Emission maximum was measured in THF/water mixture (f_w =99%). Concentration= 10 μM. [c] Quantum yield was measured in in THF/water mixture (f_w =99%).

Supplementary Fig. 60 (a) Absorption of AIEgens (10 μM) in THF solution. (b) Normalized PL spectra of AIEgens (10 μM) in THF/water mixtures (f_w = 99%). (c) Summary of photophysical properties of Z-CDPM and its isomers.

- "As the subsequent excitation energy (73.18 kcal/mol) and energy barrier (21.10 kcal/mol) were much lower than that of the former ring-opening process (90.79 kcal/mol for the excitation energy and 35.30 kcal/mol for energy barrier), INT4 would rapidly convert to DPPCYM through cyclization with the carbonyl group." This is not an argument, as photochemical processes in the excited state are not governed by excitation energy or ground state barriers per se. I would omit that sentence.

Response: Thanks a lot for your careful review and comments. We have omitted that sentence in the main text according to your valuable suggestion.

- The authors use terms like "AIE plate" or "AIE template" to describe their molecules on silica support. It is not clear if the behavior is really AIE related in these cases because luminescence could also be caused by motion restriction via interactions between solid support and the molecules instead of aggregation effects. Just replace those terms by "on solid support" or likewise.

Response: Thanks a lot for your careful review and comments. We have replaced those terms by "on solid support" in the main text according to your valuable suggestions.

- Fig. 7 please state in every instance what is the state (starting, after stimuli are given) and also state what conditions were applied where. Either in the caption or in the Figures themselves. Otherwise it is not clear which state transformations are used in each instance.

Response: We sincerely thank the reviewers for their careful examination and emphasize this issue. We acknowledge that detailed information, including the state of molecules and the type of stimulus, is conducive to understanding the color-changing process and the encryption program. Accordingly, we have added these information in the caption of Fig. 7 in the main text according to your valuable suggestions.

[*REDACTED*]

Fig. 7 Applications for information encryption. (a) Fluorescent images of different AIEgens on a TLC plate. Scale bar: 1 cm. (b) Schematic illustration of the dip-coating of *Z*-CDPM on a TLC plate and the following writing, fixing, and erasing process through UV irradiation (10 s) or heating (200 ℃ for 10 s), and the corresponding fluorescent images. Scale bar: 5 mm. (c) Photographs of the developed colored "QR code" of DPXDC. Scale bar: 5 mm. (d) Demonstration of a multicolor encryption system dual encryption models. The light irradiation was conducted under a UV lamp (0.12 mW/cm^2) at room temperature to trigger the photoarrangement of *Z*-CDPM and/or DPXDC, and the annealing procedure was performed to generate DPXC from *Z*-CDPM and/or DPXDC.

REVIEWER COMMENTS

Response to the Comments of Reviewer 1:

I believe the manuscript can be published as its.

Response: We appreciate the positive assessment from the reviewer. Thanks again for their professional comments and suggestions to improve the quality of our work.

Response to the Comments of Reviewer 2:

The authors have responded to all of my comments point-to-point and revised the manuscript accordingly. The scientific issues I raised have been adequately addressed, and the overall clarity and rigor of the manuscript have been significantly improved. I believe the current version meets the standards for publication, and I recommend that the manuscript be accepted in its current form.

Response: We appreciate the positive assessment from the reviewer. Thanks again for their professional comments and suggestions to improve the quality of our work.

Response to the Comments of Reviewer 3:

In their revision the authors have thoroughly answered to all the points raised by this reviewer. They have provided new experimental data as well as computations and have managed to improve the manuscript and the SI significantly compared to their initial submission. The new data show the robustness of the individual transformations and the photochemical and photophysical properties and the agreement between the theoretical description and the experiments is now much improved as well. For these reasons I now support publication of this work in Nature Communcations.

Response: We appreciate the positive assessment from the reviewer. Thanks again for their professional comments and suggestions to improve the quality of our work.
